# Multi-Photon-Sensitive Chromophore for the Photorelease of Biologically Active Phenols

**DOI:** 10.1021/acschemneuro.3c00552

**Published:** 2023-11-21

**Authors:** Naeem Asad, Davide Deodato, Nadeem Asad, Sangram Gore, Timothy M. Dore

**Affiliations:** † New York University Abu Dhabi, Abu Dhabi 129188, United Arab Emirates; ‡ Department of Chemistry, University of Georgia, Athens, Georgia 30602, United States

**Keywords:** photoremovable protecting groups (PPGs), photochemistry, spatiotemporal resolution, two-photon excitation, bioactive phenols

## Abstract

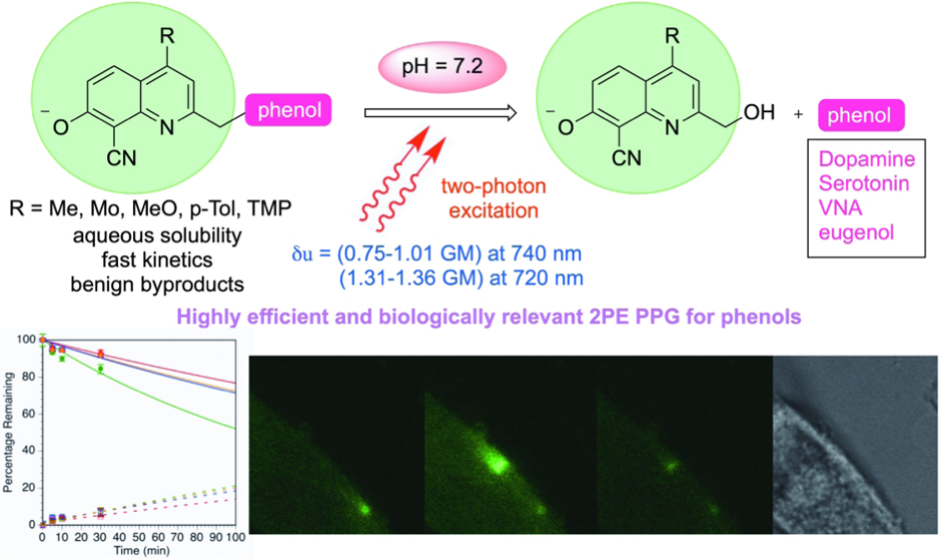

Phenols confer bioactivity to a plethora of organic compounds. Protecting the phenolic functionality with photoremovable protecting groups (PPGs) sensitive to two-photon excitation (2PE) can block the bioactivity and provide controlled release of these compounds in a spatially and temporally restricted manner by photoactivation with IR light. To develop an efficient 2PE-sensitive PPG for releasing phenols, the (8-cyano-7-hydroxyquinolin-2-yl)methyl (CyHQ) chromophore was functionalized at the C4 position with methyl, morpholine, methoxy, para-tolyl, and 3,4,5-trimethoxyphenyl groups to provide 4-methyl-CyHQ (Me-CyHQ), 4-morpholino-CyHQ (Mor-CyHQ), 4-methoxy-CyHQ (MeO-CyHQ), 4-(*p*-tolyl)-CyHQ (*p*Tol-CyHQ), and 4-(3,4,5-trimethoxyphenyl)-CyHQ (TMP-CyHQ) PPGs. The probes possess attributes useful for biological use, including high quantum yield (Φ_u_), hydrolytic stability, and good aqueous solubility in physiological conditions. The MeO-CyHQ PPG enhanced the two-photon uncaging action cross section (δ_u_) of dopamine 3.5-fold (0.85 GM) compared to CyHQ (0.24 GM) at 740 nm and 1.49 GM at 720 nm. MeO-CyHQ was used to mediate photoactivation via 2PE of serotonin, rotigotine, *N*-vanillyl-nonanoylamide (VNA) (a capsaicin analogue), and eugenol. The constructs except rotigotine showed excellent efficiency in 2PE with δ_u_ ranging from 0.75 to 1.01 GM at 740 nm and from 1.31 to 1.36 GM at 720 nm high yielding release of the payloads. These probes also performed well by using conventional single photon excitation (1PE). The spatially and temporally controlled release of dopamine from CyHQ-DA and MeO-CyHQ-DA and serotonin (5-HT) from MeO-CyHQ-5HT was quantified in cell culture by using genetically encoded sensors for dopamine and serotonin, respectively. Calcium imaging was employed to quantify the release of VNA and eugenol (EG) from MeO-CyHQ-VNA and MeO-CyHQ-EG, respectively. These tools will enable experiments to understand the intricate mechanisms involved in neurological signaling and the roles played by neurotransmitters, such as dopamine and serotonin, in the activation of their respective receptors.

## Introduction

**Figure 1:**
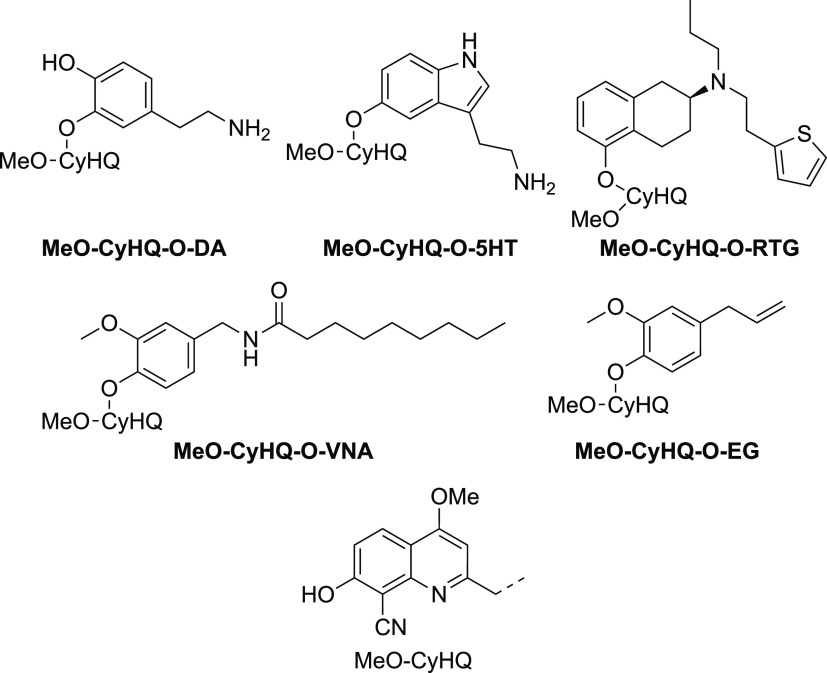
Structures of the probes developed in this work.

Phenols are a ubiquitous functional group occurring in a number of biologically active natural and synthetic compounds, such as neurotransmitters and neuromodulators, synthetically prepared agonists and antagonists of receptors and enzymes, steroids and other hormones, and amino acids. Arguably, the two most important endogenous neurotransmitters possessing phenolic functionality are dopamine (DA) and serotonin (5-HT) (Figure 1). Dopamine is the primary agonist of the dopamine receptors,^1,2^ which are divided into two subclasses, the D1- and D2-like receptors. Dopamine signaling plays an important role in the regulation of movement as well as reward-driven learning,^3,4^ and abnormal dopaminergic function has been associated with diseases such as Parkinson’s,^5^ Alzheimer’s,^6^ schizophrenia,^7^ bipolar disorder,^8^ and attention-deficit hyperactivity disorder (ADHD).^9^ During the last 20 years, a number of synthetic agonists of dopamine receptors have been developed and used as probes to investigate dopaminergic pathways or as drugs for neurological conditions. A potent synthetic agonist possessing a phenol functionality is rotigotine (RTG), an approved medication for the treatment of Parkinson’s disease and restless legs syndrome.^10,11^ Additionally, rotigotine has been shown to possess antidepressant activity, a common property among dopamine agonists.^12^

Serotonin is an aminophenol neurotransmitter that acts as a hormone in diverse tissues in the central and peripheral nervous systems.^13^ Its function is regulated by a large number of different receptors that are grouped into seven major families of receptors designated as 5-HT1–7.^14^ Serotonin plays a role in regulating mood, appetite, memory, learning, and many other cognitive functions.^13,15−17^ Furthermore, it plays a complex role in mediating pain in the CNS and periphery.^18,19^

Two other neurologically relevant phenols are *N*-vanillyl-nonanoylamide (VNA) and eugenol (EG), natural products derived from chili pepper^20^ and clove oil,^21^ respectively. They both activate ion channels belonging to the TRP family, a class of nociceptors highly expressed in the peripheral nervous system and involved in the regulation of pain.^22^ VNA is an equipotent analogue of capsaicin.^23^

Substantial efforts have been directed toward understanding the intricate mechanisms involved in neurological signaling and the roles played by neurotransmitters like dopamine and serotonin, as well as other agonists, in the activation of their respective receptors.^24−26^ A challenge in these studies is that neurotransmitters simultaneously evoke the activation of a large number of receptors such that it becomes impossible to study the outcome of these agonists on a particular receptor. A key advantage of photoactivation is that photoremovable protecting groups (PPGs) can spatially target different populations of receptors, as well as receptors with different subcellular localizations. In this regard, developing a mechanism of delivery in which neurotransmitters can be made inactive during delivery and then activating them in a spatially and temporally controlled manner when required is highly desirable. Protecting catecholamines on the phenol, as opposed to on the amine, prevents oxidation and yields faster/more efficient photorelease. The existing coumarin PPGs work poorly for phenols, so reliable options for 2PE-mediated release of this functional group are needed.^27,28^ PPGs for phenols can also be used to protect and release tyrosine on peptides, thereby substantially expanding the scope beyond drugs and catecholamines. This would eliminate off-target effects and help to develop a deeper understanding of the mechanisms involved in the functioning of these complex neurological systems. A successful technique to control receptor activation is the “photocage” strategy.^29−31^ This approach masks the activity of the neurotransmitter with a covalently bound photoremovable protecting group (PPG) that can be easily removed, restoring the biological effect, by light irradiation, an exogenous, noninvasive, and traceless stimulus. Since it was first described by Hoffman in his seminal paper on the photorelease of adenosine triphosphate (ATP),^32^ the photocage strategy has been an important technique in neuroscience and chemical biology, and hundreds of different PPGs have been developed for this use.^29−31^ Nevertheless, most of these PPGs are photolyzed only by UV illumination, and just a handful of them can be cleaved with infrared (IR) light. Radiation in the IR region is particularly useful due to its longer wavelength; it can penetrate deeper into the tissue compared to UV light, and its lower energy minimizes damage to the cells. Developing biologically useful PPGs that absorb in the near-IR region, however, requires overcoming some barriers, including inefficient photolysis, lower excited state energy, and limited solubility.^24,33^

Two-photon excitation (2PE) is an excellent alternative for releasing PPGs in the near-IR region. This photophysical phenomenon relies on the absorption of two photons simultaneously by the chromophore, thus promoting the molecule to an excited state and triggering a photolysis reaction.^34^ The efficiency of a PPG toward 2PE-mediated photolysis is defined by the two-photon uncaging action cross section (δ_u_), expressed in Göppert-Mayer (GM), which depends upon the two-photon absorbance cross section (δ_a_) and the quantum yield of the photochemical reaction (Φ_u_). Even though a number of biologically useful PPGs have been designed and synthesized, there is still a significant void in the development of PPGs that can efficiently release neurotransmitters with 2PE. Many existing chromophores exhibit one or more disadvantages: low sensitivity to 2PE, slow photorelease kinetics, or biological incompatibility (low aqueous solubility, poor cellular uptake, and toxicity).^35−40^ Coumarin-based PPGs have been among the most successful probes, possessing high δ_u_ values (>1 GM) and fast release kinetics, but their bright fluorescence upon excitation can limit their applicability in conjunction with fluorescent indicators.^41,42^ To overcome these limitations, our group has developed a class of quinoline-based PPGs, specifically (8-cyano-7-hydroxyquinolin-2-yl)methyl (CyHQ), which exhibits low levels of fluorescence while maintaining high photolysis efficiencies, extremely fast release kinetics, excellent aqueous solubilities, and an ability to protect a variety of functional groups.^43−49^

Previously, we applied this strategy to the direct release of biologically relevant phenols, although low δ_u_ values (<0.36 GM) limited the utility of these probes.^46,50^ In this work, we carried out a functionalization of the CyHQ core aimed at producing a PPG to release phenols with 2PE cross section values adequate for complex biological studies. Installing a methoxy group at position 4 of the PPG (MeO-CyHQ), significantly improved the photolysis efficiency, resulting in higher δ_u_ values (1.31–1.61 GM) than the parent CyHQ (<0.36 GM). Using this strategy, we were able to “cage” a series of phenolic bioeffectors (dopamine, serotonin, rotigotine, *N*-vanillyl-nonanoylamide, and eugenol) (Figure 1) and successfully release them with 2PE. In addition, proof-of-concept experiments in biological environments demonstrated the effectiveness of these probes for the study of receptor activation with spatiotemporal control using IR light.

## Results and Discussion

**Scheme 1:**
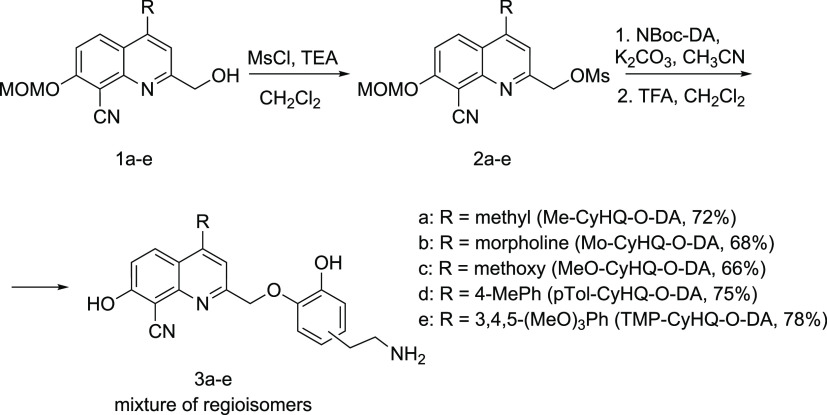
Preparation of Caged Dopamine Analogues **3a**–**e**

There are two critical requirements that a two-photon PPG must meet in order to be useful in a biological setting: (1) it must have a large δ_u_ (to be efficiently activated under 2PE) and (2) release kinetics faster than the rate of diffusion (to avoid diffusion of the neurotransmitter outside the small volume of irradiation). The CyHQ PPG has extremely fast release kinetics on the nanosecond time-scale,^44^ but possesses modest δ_u_ values, making it inadequate for applications that demand 2PE-mediated photolysis. We recently discovered that the C4 functionalization with electron-donating groups (EDGs) significantly enhances the photochemical properties of CyHQ, including the δ_u_.^45,47^ Using dopamine as a model substrate, we employed some of the best C4-substituted PPGs previously identified in our research to protect its phenolic function, with the aim to identify an efficient system for the photoactivation of phenols under 2PE. Five PPGs were selected: 4-methyl-CyHQ (Me-CyHQ), 4-morpholino-CyHQ (Mor-CyHQ), 4-methoxy-CyHQ (MeO-CyHQ), 4-(*p*-tolyl)-CyHQ (*p*Tol-CyHQ), and 4-(3,4,5-trimethoxyphenyl)-CyHQ (TMP-CyHQ). The synthetic pathway for the preparation of PPG-dopamine conjugates **3a**–**e** commenced with the activation of the hydroxyl group of **1a**–**e** (prepared as previously described)^45,47^ as a methanesulfonate, furnishing **2a**–**e**. These latter were coupled with N-Boc-dopamine using cesium carbonate as base and subsequently deprotected from the acid-labile groups using trifluoroacetic acid (TFA), affording the protected dopamine analogues **3a**–**e** (Scheme 1). All derivatives were isolated as trifluoroacetic acid salts and a mixture of regioisomers.

The photochemical and photophysical properties were investigated in a simulated physiological buffer (KMOPS, pH 7.2) and compared with the literature data for CyHQ-O-DA (Table 1). A 0.1 mM solution was easily prepared for most compounds with the exception of the *p*Tol-CyHQ-based conjugate which showed slightly lower solubility in aqueous buffer. For this derivative, 10% CH_3_CN in the buffer was used to ensure complete solubilization for the photochemistry experiments. The spontaneous hydrolysis in the dark was monitored over a period of 7 days and no significant hydrolysis was detected for any of the constructs. The molar absorptivity at the wavelength used for 1PE photolysis (ε_365_) ranged between 4800 and 5670 M^–1^ cm^–1^, in line with the values of most CyHQ-based PPGs. The UV–vis spectra showed a tail of absorption that extends above 400 nm (Supporting Information, UV–vis spectra), enabling the photolysis reaction at 405 nm (a standard wavelength on modern laser confocal microscopes), and a slight blue shift of the maximum absorption band (λ_max_) was observed for the MeO-CyHQ- and Mor-CyHQ-based constructs, compared to CyHQ.

**Table 1:**
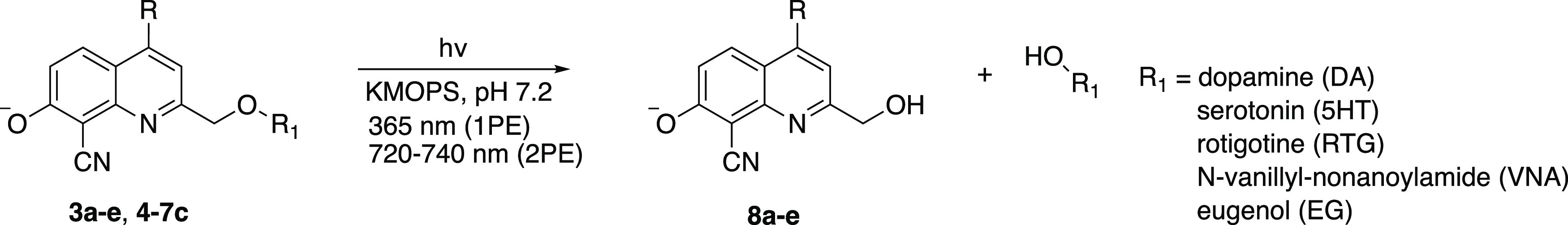
Photophysical and Photochemical Data for Photoactivatable Phenols^a^

compound	λ_max_ (nm)	ε_365_ (M^–1^ cm^–1^)	yield (%)^b^	Φ_u_	sensitivity (ε Φ_u_)	δ_u 720_ (GM)^c^	δ_u 740_ (GM)^c^	τ_d_ (h)^d^	solubility (μM)
CyHQ-O-DA^e^	365	5300	67	0.19	990	nd^f^	0.24	nh^g^	>100
Me-CyHQ-O-DA (**3a**)	365	5000	46	0.25	1235	nd	0.50	nh	>100
Mor-CyHQ-O-DA (**3b**)	358	4900	54	0.35	1728	nd	0.44	nh	>100
MeO-CyHQ-O-DA (**3c**)	354	5670	56	0.36	2045	1.49	0.85	nh	>100
*p*Tol-CyHQ-O-DA (**3d**)^h^	372	4800	54	0.07	337	nd	0.53	nh	85
TMP-CyHQ-O-DA (**3e**)	372	5210	40	0.03	142	nd	np^i^	nh	>100
MeO-CyHQ-O-5HT (**4c**)	355	5400	31	0.40	2168	1.61	1.01	nh	>100
MeO-CyHQ-O-RTG (**5c**)	343	2300	13	0.12	278	np^i^	np^i^	nh	>100
MeO-CyHQ-O-VNA (**6c**)^h^	341	3375	63	0.51	1731	1.36	0.85	nh	77
MeO-CyHQ-O-EG (**7c**)	351	7540	44	0.27	2047	1.31	0.75	nh	>100

a 0.1 mM solution in KMOPS buffer, pH 7.2.

b Chemical yield of released compounds.

c GM = 10^–50^ cm^4^ s/photon.

d Time constant of spontaneous hydrolysis in buffer in the dark at room temperature.

e Taken from the literature.^46^

f Not determined.

g No hydrolysis (<5% detected after 7 days).

h 10 or 20% of CH_3_CN added for solubilization.

i No photolysis.

**Figure 2:**
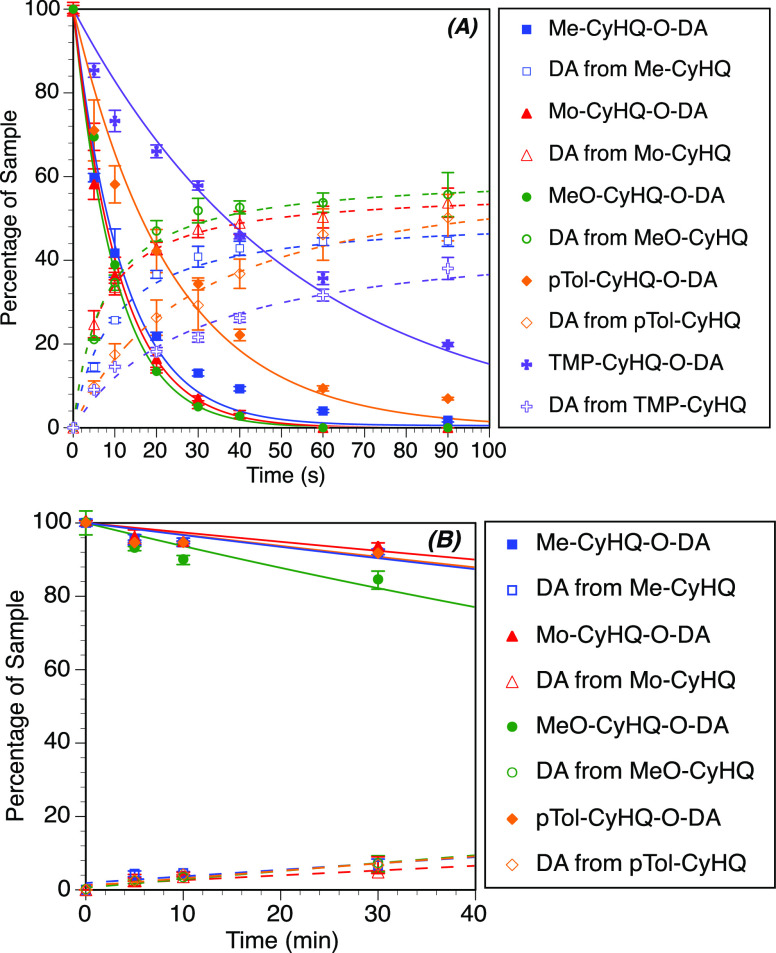
Photolysis reaction time courses of PPG-dopamine conjugates. (A) Time courses of the photolysis reactions upon 1PE with a 365 nm (5 mW power) light emitting diode (LED). Percent remaining was determined by high-performance liquid chromatography (HPLC) analysis and reported as an average of three runs (error bars represent the standard deviation). Lines are least-squares fits of a simple exponential decay (solid lines) and an exponential rise to max (dotted lines). (B) Time courses for the photolysis reactions upon 2PE (720 or 740 nm, 400–500 mW average power).

Photolysis reactions driven by 1PE were carried out using 365 nm light, and the resulting time courses are given in Figure 2A. All compounds successfully released their payload with moderate to good yields. The reaction mechanism of the photolysis follows the well-known pathway of hydroxyquinoline PPGs:^49^ after light absorption, the anionic species of **3a**–**e** is promoted to the excited state where it undergoes bond cleavage and hydrolysis, leading to the release of the phenol and formation of the benzylic alcohol remnant **4a**–**e**. Intriguingly, major differences were observed in the photolysis efficiency based on the PPG employed. With C4 aromatic substituents, the rate of the photolysis reaction was significantly slower and the quantum yields decreased by 3- to 6-fold in comparison to CyHQ-O-DA (Φ_u_ = 0.07 and 0.03 for *p*Tol-CyHQ-O-DA and TMP-CyHQ-O-DA, respectively). This observation contradicts what we previously observed for the photorelease of acetates,^47^ where the *p*Tol and TMP cages were among the most efficient PPGs, suggesting that the nature of the cleaved bond (phenol vs acetate) is a critical factor during the photochemical process. We speculate that in the case of phenols, the lower efficiency is due to a photoinduced electron transfer process between the catechol ring and the aromatic C4 substituent, which competes with the photochemical reaction in the excited state. Conversely, the introduction of heteroatom-based C4 substituents (methoxy and morpholino) had a positive effect on the photochemical properties. In this case, the reaction was completed in less than 1 min of irradiation with a 2-fold increase of quantum yield and sensitivity (Φ_u_ = 0.36 and 0.35, sensitivity (ε Φ_u_) = 2045 and 1728 for MeO-CyHQ-O-DA and Mor-CyHQ-O-DA, respectively). No significant improvement in the photochemical properties was noted with the introduction of a 4-methyl group (Me-CyHQ-O-DA).

We next investigated the ability of the photolysis reactions to be driven by 2PE using a Ti:sapphire laser as a light source. Most of the PPG-dopamine conjugates were efficiently photolyzed by 2PE (Figure 2B), releasing their payload with superior δ_u_ values than CyHQ-O-DA (Table 1). The only exception was TMP-CyHQ-O-DA (**3e**), whose photolysis could not be executed through 2PE, consistent with what was observed during the 1PE experiments. Intriguingly, MeO-CyHQ-O-DA (**3c**) emerged as the best photoactivatable dopamine derivative (δ_u_ = 1.49 GM), which was 6-fold higher than that of CyHQ-O-DA (0.24 GM). The photolysis of MeO-CyHQ-O-DA was carried out at 720 nm since its λ_max_ is blue-shifted (354 nm) compared to the rest of the tested molecules. Under these conditions, up to 28% photolysis was achieved within 30 min of irradiation (Figure 2B) and a significant amount of released dopamine was detected in solution.

**Scheme 2:**
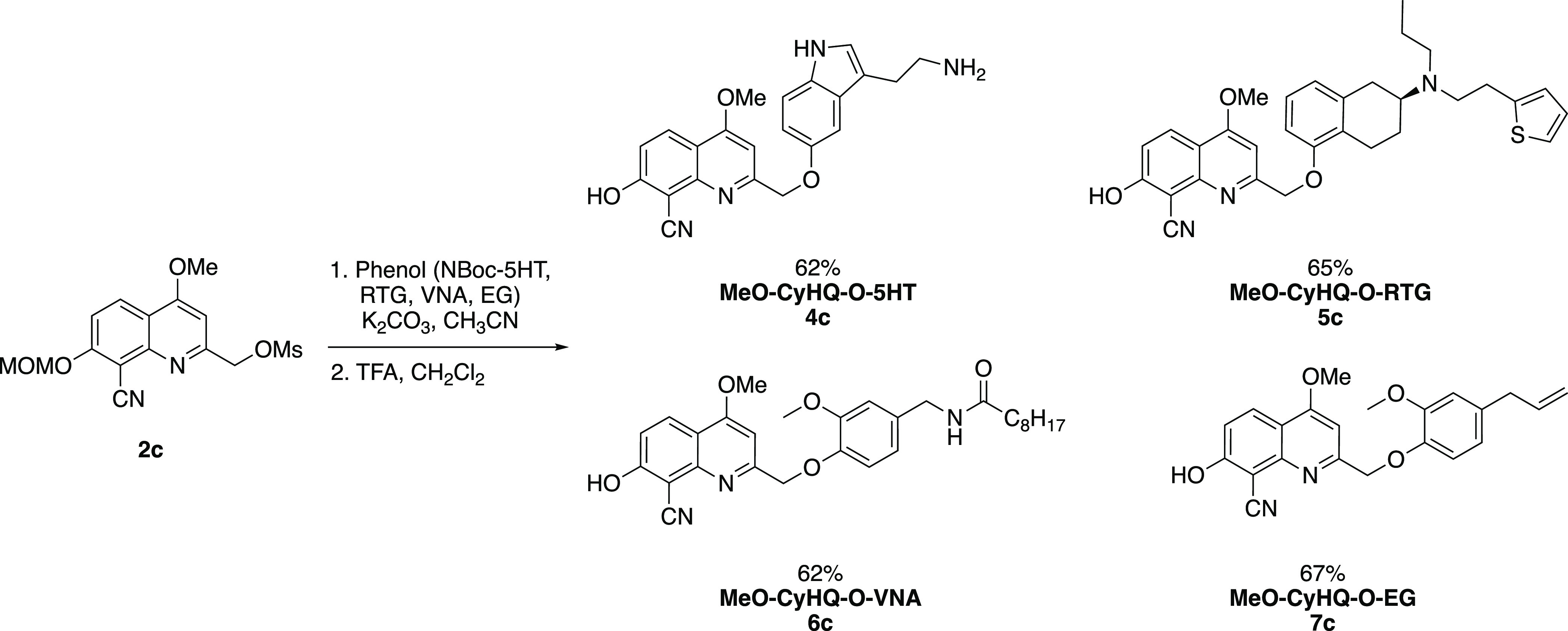
Preparation of Caged Phenolic Bioeffectors **4**–**7c**

From the proof-of-concept investigation using dopamine as a model substrate, MeO-CyHQ emerged as the most efficient PPG. We used this PPG for the photoactivation of other biologically relevant phenols: serotonin, rotigotine, *N*-vanillyl-nonanoylamide, and eugenol. For the preparation of these probes, we followed the same procedure used for the PPG-dopamine conjugates, starting from mesylate **2c**. The two-step sequence, involving coupling with the appropriate phenol in basic media followed by deprotection with trifluoroacetic acid, afforded MeO-CyHQ-protected phenols **4**–**7c** (Scheme 2).

**Figure 3:**
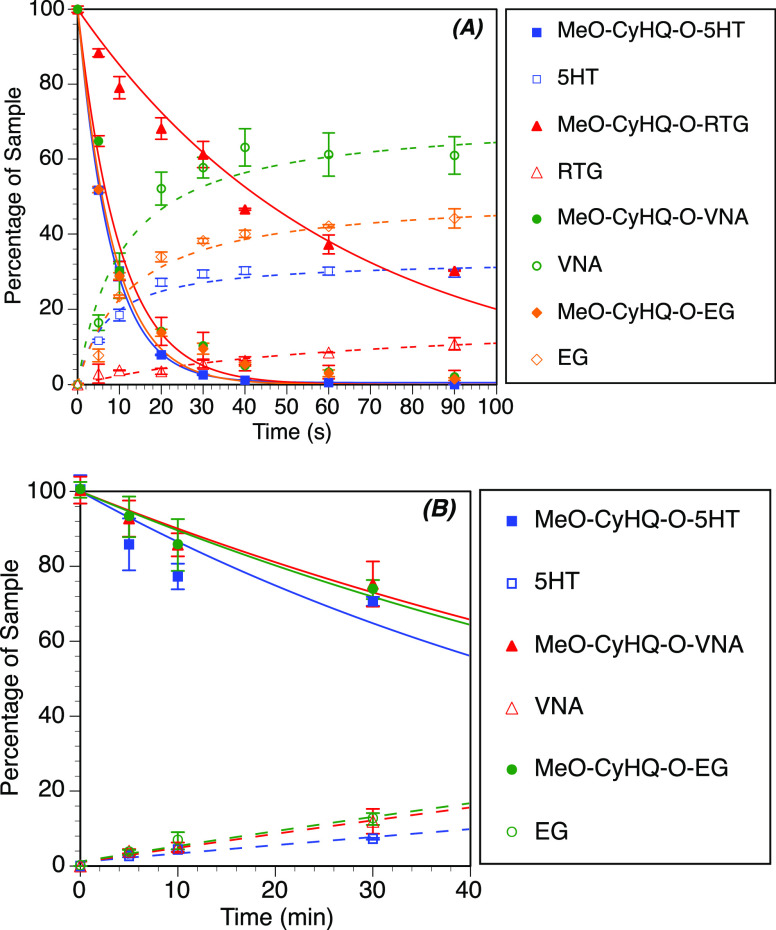
Photolysis reaction time courses of MeO-CyHQ conjugates **4**–**7c**. (A) Time courses of the photolysis reactions upon 1PE with a 365 nm (5 mW power) LED. Percent remaining was determined by HPLC analysis and reported as an average of three runs (error bars represent the standard deviations). Lines are least-squares fits of a simple exponential decay (solid lines) and an exponential rise to max (dotted lines). (B) Time courses for the photolysis reactions on 2PE (720 nm, 400–500 mW average power).

The photochemical behavior of constructs **4**–**7c** was investigated in KMOPS buffer at the concentration of 0.1 mM. Under these conditions, all compounds were soluble except for the VNA analogue **6c**, which, because of the lipophilicity of the vanilloid, required 20% CH_3_CN for full solubilization. The stability toward hydrolysis in the dark was monitored over a week, and no significant decomposition was detected for any probe. All constructs were successfully photolyzed after light exposure, as shown in the time courses of the photolysis reactions (Figure 3). MeO-CyHQ constructs **4c**, **6c**, and **7c** were photoactivated with excellent quantum efficiency and sensitivities, and good chemical yields were obtained by monitoring the appearance of the released bioeffector over the time course of the photoreaction (Table 1). The rotigotine probe **5c** displayed low quantum yield and sensitivity (0.12 and 278, respectively) compared to the rest of the series (Φ_u_ = 0.27–0.51 and ε Φ_u_ = 1731–2168, respectively). Additionally, MeO-CyHQ-O-RTG did not undergo photorelease through 2PE (vide infra), confirming its poor photochemical efficiency. We believe this effect is due to the presence of the thiophene ring, a well-known photoactive group,^51^ which could act as an energy sink in the excited state and quench the photochemical reaction.

The photolysis reaction on **4c**, **6c**, and **7c** could be successfully performed through 2PE. For these experiments, we employed two different wavelengths (720 and 740 nm) and, as in the case of dopamine derivative **3c**, the photochemical reactions were more efficient with 720 nm light (Table 1). Under these conditions, high values of two-photon uncaging action cross section were obtained (δ_u_ = 1.31–1.61 GM), which is a 4- to 6-fold increase in comparison to previously reported CyHQ-protected phenols.^46,50^

Considering the excellent dark stability, good solubility, and larger 2PE cross section, the 4-MeO-CyHQ conjugates with dopamine and serotonin were tested by exploring the activation of the respective genetically encoded sensors dLight 1.2^52,53^ and GRAB5-HT^54^ expressed on the surface of HCT 116 cells. The efficacy of the MeO-CyHQ constructs of VNA and eugenol was evaluated by activating the TRPV1 receptor expressed on the membrane of HEK 293 cells through calcium imaging.

To establish the cellular assay for photoactivatable dopamine and serotonin, we tested CyHQ-O-DA for its ability to release dopamine and activate dLight because it was previously shown to activate the dopamine-1 receptor on MDA-MB-231 cells in culture and the dopamine-2 receptor in mouse brain slice.^46^ HCT 116 cells expressing dLight 1.2 were treated with CyHQ-O-DA (10 μM), which released dopamine after exposure to a 405 nm light pulse (1PE), as shown by the increase in fluorescence output from the dLight sensor (Figure S2, Video S9), similar to the addition of bulk dopamine (100 μM) to dLight expressing HCT 116 cells in culture (Figure S1A, Video S3). No response from dLight 1.2 was observed when the cells were exposed to 405 nm light in the absence of CyHQ-O-DA or any other photoactivatable dopamine (Figure S1C, Video S5). The response depended on the duration of the light pulse delivered (Figure S2A–C). Robust fluorescent signals were observed with different CyHQ-O-DA concentrations and light pulse durations (Figure S2D–G, Videos S10–S12).

In HCT 116 cell culture, MeO-CyHQ-O-DA (10 μM) responded to a 10 ms flash of 405 nm light, releasing dopamine in an amount sufficient to cause a robust response from the dLight sensor expressed on the membrane, which then decayed as expected to baseline (Figure S3D–E, Videos S14 and S15). MeO-CyHQ-O-DA also showed a dependence on the duration of the 405 nm light pulse; the signal from dLight 1.2 increased with increasing length of the exposure (Figure S3A–C, Video S13). The excitation laser was directed at different places along the membrane of the cell, and the response was recorded. Between 50 and 100 ms durations, the response plateaus, indicating saturation of dopamine around the dLight sensor expressed on the membrane (Figure S3C). In a similar experiment, the cells were bathed with MeO-CyHQ-O-DA (10 μM) and then exposed to increasing durations of light bursts at the same location. The dLight response increased in intensity and spread along the membrane as larger amounts of dopamine were released (Figure S3F and Video S16).

**Figure 4:**
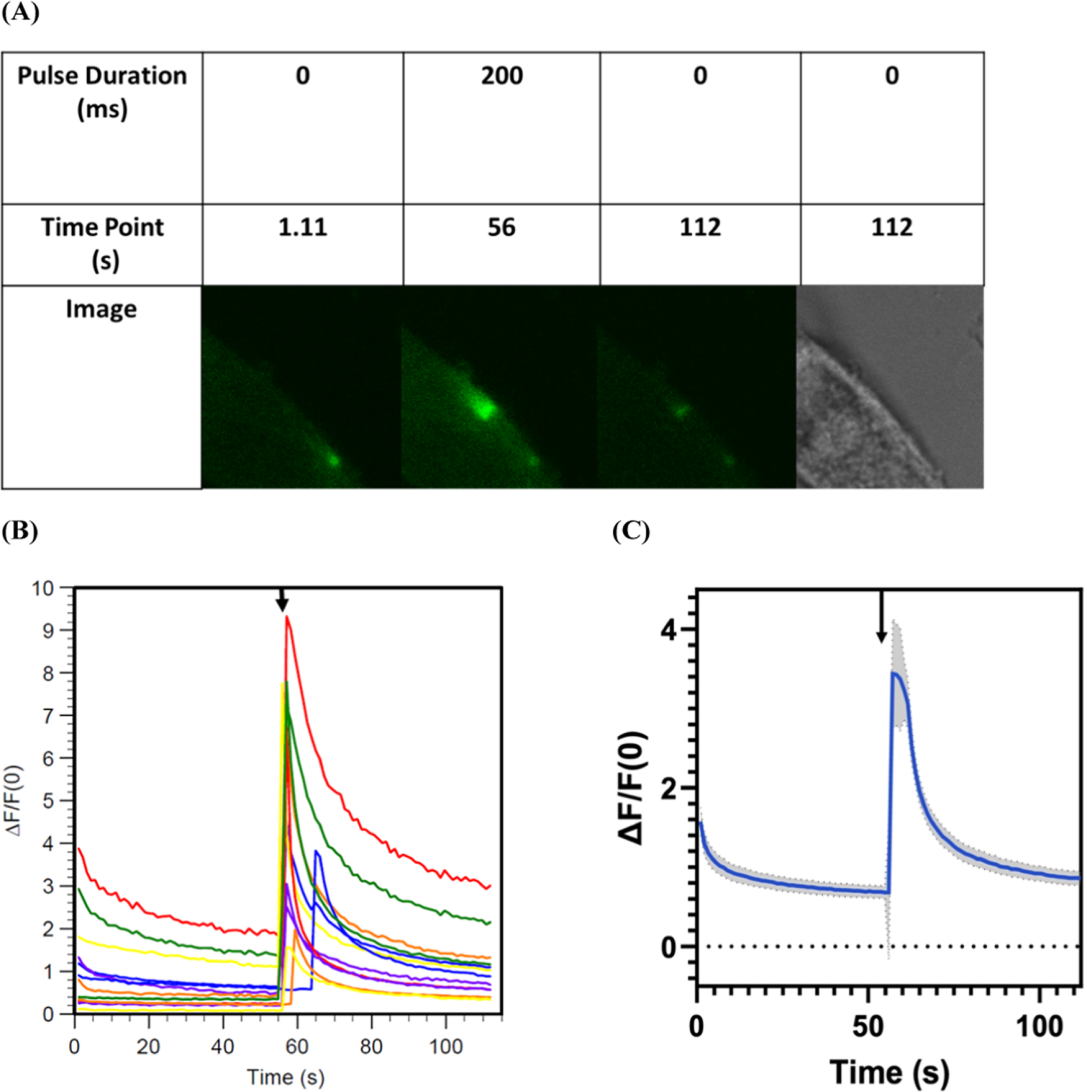
Dopamine released in a controlled spatial and temporal manner from MeO-CyHQ-O-DA via 2PE (720 nm) as depicted by the activation of dLight 1.2 expressed on the membrane of HCT 116 cells. (A) Response of dLight to a single, 200 ms pulse of 720 nm light to activate MeO-CyHQ-O-DA (100 μM). See Video S1. (B) Plot of Δ*F*/*F*_0_ vs time (s) for MeO-CyHQ-O-5HT (100 μM) measured in nine experiments. (C) Plot of average Δ*F*/*F*_0_ vs time (s) for MeO-CyHQ-O-DA (100 μM) from nine experiments. Gray areas show the standard deviation of the measurement. Arrow marks the time point of the 200 ms light pulse.

Having established that photoactivation of MeO-CyHQ-O-DA caused dLight 1.2 to fluoresce via 1PE, we used the compound to mediate the release of dopamine via 2PE. HCT 116 cells expressing dLight 1.2 were treated with MeO-CyHQ-O-DA (100 μM). A 200 ms flash of 720 nm light from a Ti:sapphire laser directed near the membrane of a single cell released dopamine at the focus of the beam and activated dLight 1.2 (Figure 4 and Video S1). The response of the detector was robust, and the fluorescence signal decayed as expected. In the absence of MeO-CyHQ-O-DA, dLight 1.2 gave no response to a 300 ms, 720 nm pulse of light (Figure S1E and Video S7), showing that dLight does not respond to light alone. Light-dosing experiments were not possible using 2PE because damage to the cells occurred at a pulse duration of 500 ms and longer. The repetitive application of light pulses and the longer duration of pulses damaged the culture plates and the cells.

Similar experiments releasing serotonin from MeO-CyHQ-O-5HT via 1PE and 2PE were conducted using GRAB5-HT^54^ expressed on the membrane of HCT 116 cells as the serotonin sensor (Figure S4). MeO-CyHQ-O-5HT exhibited a dependence on the increase in the duration of the light pulse at 405 nm (Figure S4A–C, Video S17). The excitation laser was aimed at different points along the cell membrane in an inclined gradient. The response plateaued out at around 200 ms pulse duration, indicating saturation of serotonin in the vicinity of the serotonin receptor on the cell membrane. MeO-CyHQ-O-5HT (20 μM) responded to a 20 ms burst of 405 nm light, releasing a sufficient amount of serotonin to trigger a robust response from the GRAB5-HT receptor expressed on the cellular membrane (Figure S4D,E, Video S18). The response decayed to baseline, similarly to dopamine.

**Figure 5:**
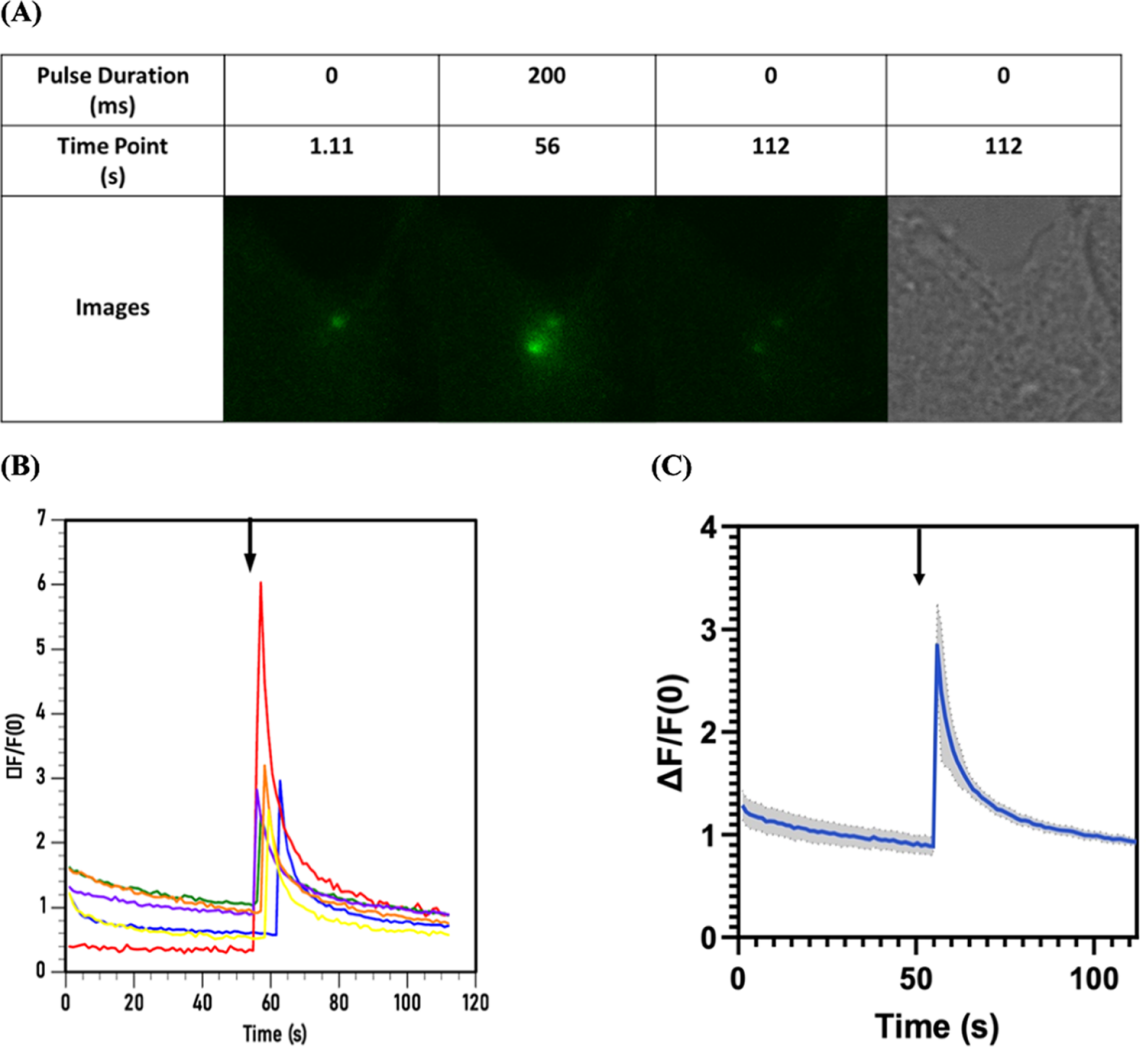
Serotonin released in a controlled spatial and temporal manner from MeO-CyHQ-O-5HT via 2PE (720 nm) as depicted by the activation of GRAB5-HT expressed on the membrane of HCT 116 cells. (A) Response of GRAB5-HT to a single, 200 ms pulse of 720 nm light to activate MeO-CyHQ-O-DA (200 μM) (see Video S2). (B) Plot of Δ*F*/*F*_0_ vs time (s) for MeO-CyHQ-O-5HT (200 μM) measured in six experiments. (C) Plot of average Δ*F*/*F*_0_ vs time (s) for MeO-CyHQ-O-5HT (100 μM) from each of six experiments. Gray areas show the standard deviation of the measurement. The arrow marks the time point of the 200 ms light pulse.

The same GRAB5-HT and MeO-CyHQ-O-5HT system was used for the 2PE-mediated release of serotonin. A 200 ms pulse of 720 nm light from a Ti:sapphire laser directed near the membrane of the cell resulted in an increase in serotonin concentration to activate the GRAB5-HT receptor (Figure 5 and Video S2). A robust response with a subsequent decay of the fluorescence signal was observed. No response was observed only in the presence of a 300 ms burst of 720 nm light (Figure S1F, Video S8) in the absence of MeO-CyHQ-O-5HT showing that GRAB5-HT does not respond to light alone.

Calcium imaging of HEK 293 cells expressing TRPV1 receptors on the cell surface was carried out to study the release of VNA and EG from MeO-CyHQ-VNA and MeO-CyHQ-EG, respectively. The conditions were optimized for both 1PE- and 2PE-mediated photoactivation. MeO-CyHQ-VNA (2.5 μM) released VNA in sufficient amounts to elicit a robust response from the calcium-sensitive dye Fluo-4 using a 50 ms pulse of 405 nm wavelength light (Figure S11, Video S21) and EG was released from 5 μM MeO-CyHQ-EG with a 500 ms pulse of 405 nm light (Figure S12, Video S22). These results demonstrate that VNA is a more potent activator of TRPV1 channels.

**Figure 6:**
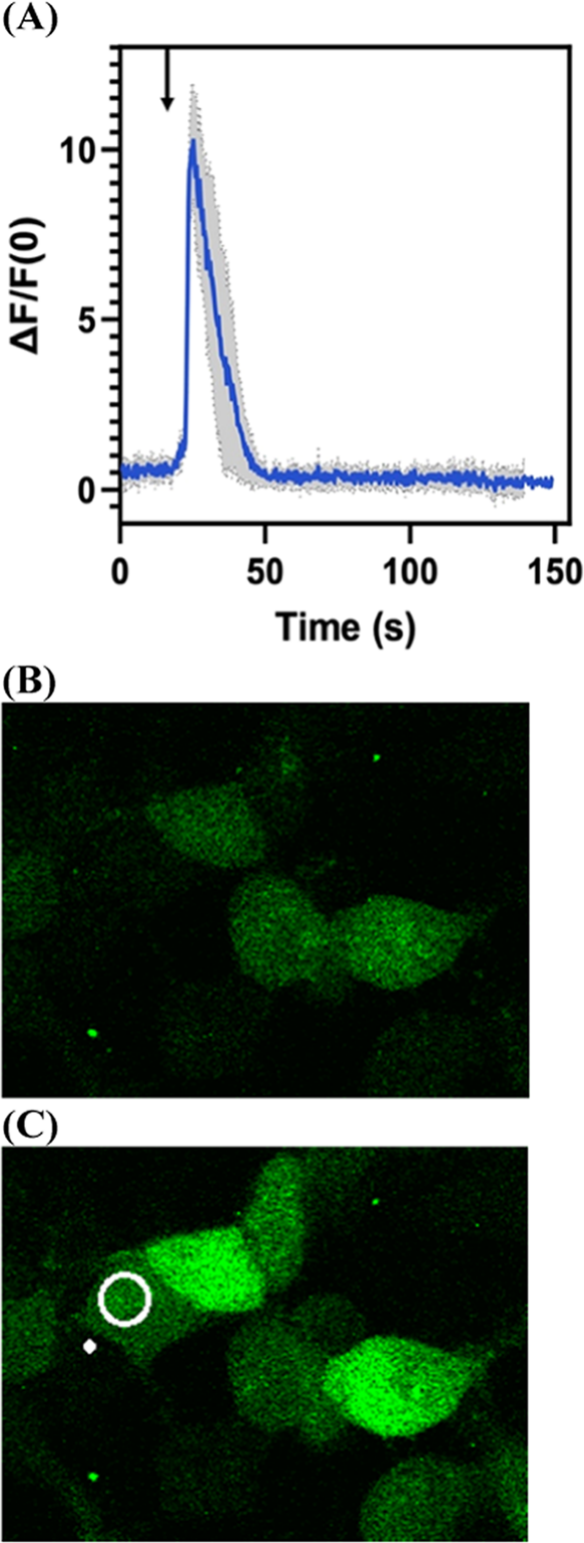
Calcium influx measured in HEK 293 cells stably expressing TRPV1 channels after activation of MeO-CyHQ-VNA with a 250 ms flash of 740 nm light (20% power). (A) Plot of Δ*F*/*F*_0_ vs time (s) for MeO-CyHQ-VNA (5 μM). The graph is an average of three different experiments. Gray areas show the standard deviation of the measurement. The black arrow indicates the timing of the light flash at the 30 s time point. (B) Image of cells before light exposure. (C) Image of cells at peak response of the fluorescent Ca^2+^ indicator (Fluo-4). See Video S19.

**Figure 7:**
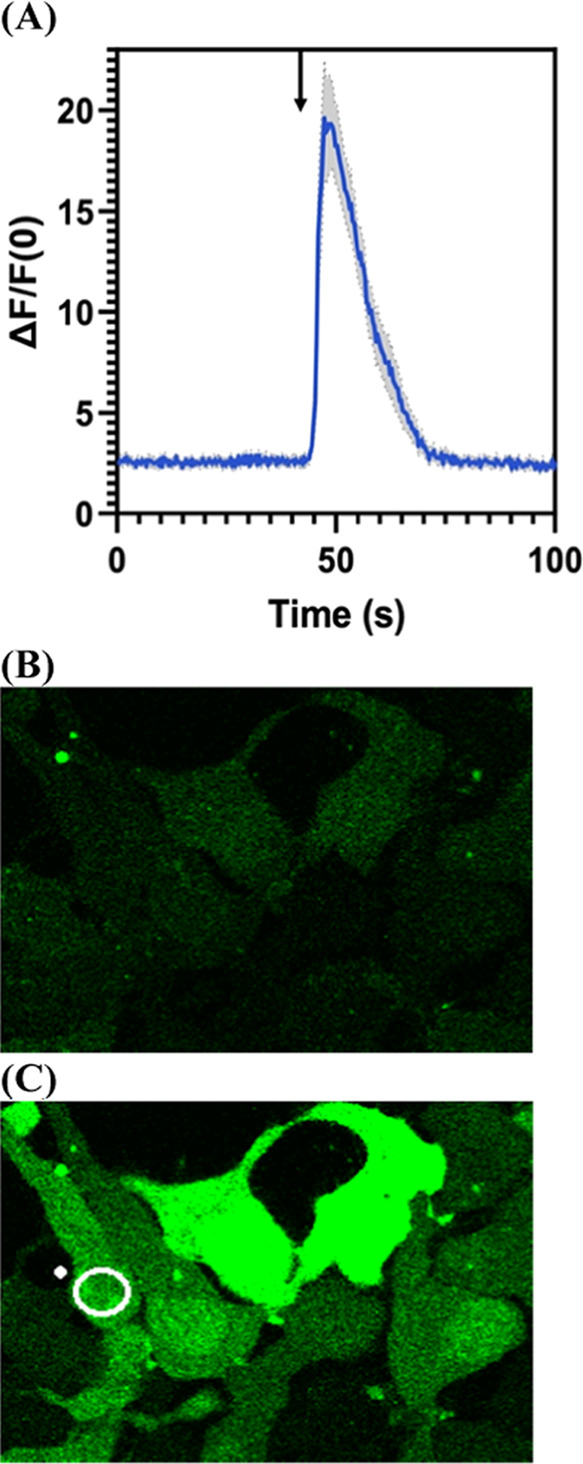
Calcium influx measured in HEK 293 cells stably expressing TRPV1 channels after activation of MeO-CyHQ-EG with a 500 ms flash of 740 nm light (20% power). (A) Plot of Δ*F*/*F*_0_ vs time (s) for MeO-CyHQ-EG (5 μM). The graph is an average of three different experiments. Gray areas show the standard deviation of the measurement. The black arrow indicates the timing of the light flash (43 s). (B) Image of the cell before light exposure. (C) Image of cells at peak response of the fluorescent Ca^2+^ indicator (Fluo-4). See Video S20.

Using the same TRPV1-expressing HEK 293 cell line, MeO-CyHQ-VNA (5 μM) released a sufficient amount of VNA to generate a robust response from Fluo-4 through 2PE by a pulse of 250 ms, 740 nm light (Figure 6 and Video S19). Owing to its lower potency, a 500 ms light pulse was required to obtain a sufficient response when MeO-CyHQ-EG was used to activate the TRPV1 receptors on the cell surface (Figure 7 and Video S20). Notably, CyHQ-VNA and CyHQ-EG did not induce any response from Fluo-4 upon exposure to 740 nm light, indicating no 2PE-mediated activation of TRPV1 receptors (data not shown). In the absence of MeO-CyHQ-VNA or MeO-CyHQ-EG, the Fluo-4 indicator did not respond to bursts of 400- (1PE) or 740 nm (2PE) light (data not shown).

## Conclusions

The C4 position of the CyHQ PPG was derivatized to generate five analogues: Me-CyHQ, Mo-CyHQ, MeO-CyHQ, *p*Tol-CyHQ, and TMP-CyHQ. These constructs were used to study the release of dopamine through 1PE and 2PE. The two-photon uncaging action cross section (δ_u_) of MeO-CyHQ-O-DA at 740 nm was enhanced 3.5-fold compared CyHQ-O-DA. The value of δ_u_ was higher at 720 nm (1.49 vs 0.85 GM). The MeO-CyHQ PPG was tested for its ability to mediate the release of serotonin, rotigotine, VNA, and eugenol via 1PE and 2PE. With the exception of rotigotine, all of the bioactive phenols were efficiently released through 1PE and 2PE (Φ_u_ = 0.27–0.51 and δ_u_ = 0.75–0.85 GM at 740 nm and δ_u_ = 1.31–1.61 GM at 720 nm). Using genetically encoded fluorescent sensors of dopamine (dLight 1.2) and serotonin (GRAB5-HT) and the calcium sensor Fluo-4, we showed that the biological effectors could be released in sufficient quantity through 1PE and 2PE to generate robust signals from the sensors in cell culture. Further optimization efforts will focus on optimizing the concentration of the photoactivatable compounds to use a shorter pulse duration and a lower average laser power than the 24 and 15 mW used here. For comparison, a 0.2 ms pulse of 720 nm light (unreported average power) released a sufficient amount of glutamate from 10 mM MNI-Glu to activate a fluorescent probe of glutamate (iGluSnFR).^55^ The probes are excellent for use on biological preparations due to sufficient solubility in aqueous media at physiological pH, excellent hydrolytic stability in the dark, fast kinetics, and the production of benign byproducts after photocleavage. Overall, we created an efficient PPG with sensitivity toward 2PE to release phenols in biologically relevant systems. The tools developed in this study can be used to study the action of these biologically active molecules in tissues.

## Methods

### Synthesis

#### General

Commercially available reagents and solvents were employed without further purification. ^1^H and ^13^C NMR spectra were recorded on a Bruker Avance III HD 500 and 600 MHz NMR spectrometer. A Lambda 25 UV–vis–NIR spectrophotometer (PerkinElmer) recorded the UV spectra. The uHPLC analysis and preparative HPLC purifications were performed on an Agilent Infinity series system equipped with an autosampler and diode array detector using Zorbax Eclipse C-18 reversed-phase columns, having a mobile phase composed of water with 0.1% TFA and acetonitrile. An Agilent 6540 HD Accurate Mass QTOF/LC/MS with electrospray ionization (ESI) measured the high-resolution mass spectrometry (HRMS). Purification of the compounds was carried out by flash chromatography on an Isolera Spektra 4 with Biotage SNAP cartridges packed with KPSIL silica. An aqueous solution of 100 mM KCl and 10 mM MOPS (3-(N-morpholino)propanesulfonic acid) titrated to pH 7.2 with 0.1 N NaOH afforded the KMOPS buffer.

#### General Procedure for the Preparation of 4-R-CyHQ-OMs **2a**–**e**

The alcohols 4-R-CyHQ-OH (0.68 mmol) and triethylamine (0.38 mL, 2.72 mmol) were dissolved in CH_2_Cl_2_ (20 mL) in a round-bottom flask and cooled to 0 °C in an ice bath. Methanesulfonyl chloride (0.16 mL, 2.05 mmol) was added to this solution in a dropwise manner. The ice was removed and the reaction was stirred at room temperature for 12 h. After the completion of the reaction, it was diluted with CH_2_Cl_2_ (150 mL), washed with H_2_O (3 × 100 mL) and brine (3 × 100 mL), dried over MgSO_4_, and concentrated to dryness. The resulting residue was purified by column chromatography (0–70% EtOAc in *n*-hexane, gradient elution), yielding the corresponding 4-R-CyHQ-mesylates **2a**–**e**.

#### (8-Cyano-7-(methoxymethoxy)-4-methylquinolin-2-yl)methylmethanesulfonate (**2a**)

(201 mg, 0.60 mmol, 88% yield). ^1^H NMR (500 MHz, chloroform-*d*) δ 8.27–8.10 (m, 1H), 7.64–7.54 (m, 1H), 7.40 (s, 1H), 5.52 (s, 2H), 5.48 (s, 2H), 3.61 (s, 3H), 3.30 (s, 3H), 2.75 (s, 3H); ^13^C NMR (126 MHz, chloroform-*d*) δ 162.1, 156.7, 148.4, 146.6, 130.0, 123.0, 119.8, 115.7, 114.8, 95.1, 71.7, 67.1, 56.9, 38.5, 18.8; HRMS (ESI-QTOF) *m*/*z*: [M + H]^+^ calcd for C_15_H_16_N_2_O_5_S, 337.0853; found, 337.0852.

#### (8-Cyano-7-(methoxymethoxy)-4-morpholinoquinolin-2-yl)methylmethanesulfonate (**2b**)

(254 mg, 0.63 mmol, 92% yield). ^1^H NMR (500 MHz, chloroform-*d*) δ 8.18 (dd, *J* = 9.5, 2.9 Hz, 1H), 7.51 (dd, *J* = 9.4, 2.9 Hz, 1H), 6.99 (d, *J* = 2.8 Hz, 1H), 5.64–5.28 (m, 4H), 4.09–3.83 (m, 4H), 3.70–3.49 (m, 3H), 3.41–3.02 (m, 7H); ^13^C NMR (126 MHz, chloroform-*d*) δ 162.1, 158.4, 157.7, 150.2, 129.8, 117.8, 114.9, 114.4, 106.6, 100.1, 95.0, 72.0, 66.7, 56.9, 52.7, 38.5; HRMS (ESI-QTOF) *m*/*z*: [M + H]^+^ calcd for C_18_H_21_N_3_O_6_S, 408.1224; found, 408.1222.

#### (8-Cyano-4-methoxy-7-(methoxymethoxy)quinolin-2-yl)methylmethanesulfonate (**2c**)

(204 mg, 0.58 mmol, 85% yield). ^1^H NMR (500 MHz, chloroform-*d*) δ 8.33 (d, *J* = 9.6 Hz, 1H), 7.48 (d, *J* = 9.5 Hz, 1H), 6.90 (s, 1H), 5.49 (s, 2H), 5.46 (s, 2H), 4.12 (d, *J* = 7.8 Hz, 3H), 3.60 (s, 3H), 3.30 (s, 3H); ^13^C NMR (126 MHz, chloroform-*d*) δ 163.6, 162.7, 158.6, 149.5, 128.4, 115.9, 114.8, 114.6, 99.1, 98.4, 95.0, 72.1, 56.9, 56.3, 38.4; HRMS (ESI-QTOF) *m*/*z*: [M + H]+ calcd for C_15_H_16_N_2_O_6_S, 353.0802; found, 353.0816.

#### (8-Cyano-7-(methoxymethoxy)-4-(*p*-tolyl)quinolin-2-yl)methylmethanesulfonate (**2d**)

(243 mg, 0.59 mmol, 87% yield). ^1^H NMR (500 MHz, chloroform-*d*) δ 8.14 (d, *J* = 9.6 Hz, 1H), 7.52 (d, *J* = 9.5 Hz, 1H), 7.48 (d, *J* = 2.9 Hz, 1H), 7.38 (s, 4H), 5.59 (s, 2H), 5.47 (s, 2H), 3.59 (d, *J* = 1.9 Hz, 3H), 3.32 (s, 3H), 2.49 (s, 3H); ^13^C NMR (126 MHz, chloroform-*d*) δ 162.3, 156.5, 150.7, 149.0, 139.4, 133.6, 132.4, 129.6, 129.5, 121.7, 119.2, 115.9, 114.9, 95.0, 71.6, 57.0, 52.6, 38.5, 21.3; HRMS (ESI-QTOF) *m*/*z*: [M + H]^+^ calcd for C_21_H_20_N_2_O_5_S, 413.1166; found, 413.1162.

#### (8-Cyano-7-(methoxymethoxy)-4-(3,4,5-trimethoxyphenyl)quinolin-2-yl)methylmethanesulfonate (**2e**)

(297 mg, 0.61 mmol, 90% yield). ^1^H NMR (500 MHz, chloroform-*d*) δ 8.15 (d, *J* = 9.5 Hz, 1H), 7.53 (d, *J* = 9.5 Hz, 1H), 7.49 (s, 1H), 6.65 (s, 2H), 5.58 (s, 2H), 5.46 (s, 2H), 3.95 (s, 3H), 3.90 (s, 6H), 3.59 (s, 3H), 3.31 (s, 3H); ^13^C NMR (126 MHz, chloroform-*d*) δ 162.3, 156.5, 153.5, 150.5, 149.0, 138.8, 132.2, 132.0, 121.6, 119.0, 116.0, 114.7, 106.7, 99.8, 95.0, 71.6, 61.0, 57.0, 56.4, 38.4; HRMS (ESI-QTOF) *m*/*z*: [M + H]+ calcd for C_23_H_24_N_2_O_8_S, 489.1326; found, 489.1316.

#### General Procedure for the Preparation of 4-R-CyHQ-Protected Dopamine and Phenol Derivatives (**3a**–**e** and **4**–**7c**)

The phenols (0.06 mmol) were dissolved in acetonitrile (3 mL) followed by the addition of potassium carbonate (25 mg, 0.18 mmol) and MOM-protected methanesulfonate ester **2a**–**d** (0.08 mmol). The reaction was stirred at 70 °C with constant monitoring by thin-layer chromatography (TLC) or HPLC. The reactions were completed in 1–3 h. After completion, the solvent was evaporated under reduced pressure and the residue was dissolved in CH_2_Cl_2_ (20 mL), washed with H_2_O (3 × 10 mL) and brine (3 × 10 mL), dried over MgSO_4_, and concentrated to dryness. The resulting residue was again dissolved in CH_2_Cl_2_ (2 mL). TFA (0.2 mL) was added dropwise. The reaction was stirred at room temperature in the dark and constantly monitored by HPLC until complete consumption of the starting material (2–5 h) was observed. The solvent was completely evaporated, and the compounds were purified by reversed-phase column chromatography utilizing a gradient of solvents A and B, where A is 0.1% TFA in water and B is pure acetonitrile. The gradient for the purification process started with 100% A and 0% B and finished with 0% A and 100% B in the 12 min method to yield pure CyHQ-phenols.

#### **2**-(3-((8-Cyano-7-hydroxy-4-methylquinolin-2-yl)methoxy)-4-hydroxyphenyl)ethan-1-aminium 2,2,2-trifluoroacetate (**3a**)

(20 mg, 0.043 mmol, 72% yield). ^1^H NMR (500 MHz, methanol-*d*_4_) δ 8.21 (dd, *J* = 9.3, 4.1 Hz, 1H), 7.54 (d, *J* = 17.0 Hz, 1H), 7.29 (dd, *J* = 9.3, 2.3 Hz, 1H), 7.09–7.00 (m, 1H), 6.87–6.80 (m, 1H), 6.71 (ddd, *J* = 39.9, 8.1, 2.1 Hz, 1H), 5.39 (d, *J* = 13.4 Hz, 2H), 3.13 (td, *J* = 7.7, 4.7 Hz, 2H), 2.84 (t, *J* = 7.6 Hz, 2H), 2.70 (d, *J* = 5.0 Hz, 3H); ^13^C NMR (126 MHz, methanol-*d*_4_) δ 164.0, 160.0, 148.4, 147.2, 146.9, 146.8, 146.0, 130.3, 127.8, 121.9, 121.4, 119.6, 118.9, 118.7, 117.3, 116.0, 114.6, 94.5, 71.6, 40.7, 32.7, 17.3; HRMS (ESI-QTOF) *m*/*z*: [M – CF_3_CO_2_]^+^ calcd for C_20_H_19_N_3_O_3_, 350.1499; found, 350.1496.

#### 2-(3-((8-Cyano-7-hydroxy-4-morpholinoquinolin-2-yl)methoxy)-4-hydroxyphenyl)ethan-1-aminium 2,2,2-trifluoroacetate (**3b**)

(21 mg, 0.04 mmol, 68% yield). ^1^H NMR (500 MHz, acetonitrile-*d*_3_) δ 8.04 (dd, *J* = 9.4, 3.4 Hz, 1H), 7.33 (dd, *J* = 9.5, 3.6 Hz, 1H), 7.10–6.99 (m, 2H), 7.00–6.65 (m, 4H), 5.31 (d, *J* = 22.7 Hz, 2H), 3.87 (dd, *J* = 5.6, 3.8 Hz, 4H), 3.63 (t, *J* = 4.5 Hz, 4H), 3.18 (s, 2H), 2.93–2.76 (m, 2H); ^13^C NMR (126 MHz, CD_3_CN) δ 167.0, 160.1, 153.9, 147.5, 146.2, 145.0, 144.6, 132.1, 128.3, 123.8, 120.4, 116.8, 116.7, 116.4, 115.1, 113.6, 112.7, 104.8, 89.8, 69.5, 66.1, 52.5, 41.3, 32.1, 14.6; HRMS (ESI-QTOF) *m*/*z*: [M – CF_3_CO_2_]^+^ calcd for C_23_H_24_N_4_O_4_, 421.1870; found, 421.1872.

#### 2-(4-((8-Cyano-7-hydroxy-4-methoxyquinolin-2-yl)methoxy)-3-hydroxyphenyl)ethan-1-aminium 2,2,2-trifluoroacetate (**3c**)

(19 mg, 0.04 mmol, 66% yield). ^1^H NMR (500 MHz, acetonitrile-*d*_3_) δ 8.17 (dd, *J* = 9.2, 3.3 Hz, 1H), 7.31 (d, *J* = 9.2 Hz, 1H), 7.11–7.06 (m, 2H), 6.87 (d, *J* = 8.1 Hz, 1H), 6.84–6.79 (m, 1H), 6.71 (dd, *J* = 8.2, 2.0 Hz, 1H), 6.62 (s, 2H), 5.33 (d, *J* = 12.9 Hz, 2H), 4.09 (d, *J* = 4.5 Hz, 3H), 3.19 (p, *J* = 6.2 Hz, 2H), 2.88 (t, *J* = 8.2 Hz, 2H); ^13^C NMR (126 MHz, acetonitrile-*d*_3_) δ 165.1, 164.7, 164.7, 160.8, 147.7, 146.5, 146.3, 145.7, 131.4, 128.3, 128.0, 123.4, 120.3, 116.5, 116.1, 114.1, 99.0, 72.5, 72.2, 56.8, 41.4, 32.0; HRMS (ESI-QTOF) *m*/*z*: [M – CF_3_CO_2_]^+^ calcd for C_20_H_19_N_3_O_4_, 366.1448; found, 366.1446.

#### 2-(3-((8-Cyano-7-hydroxy-4-(*p*-tolyl)quinolin-2-yl)methoxy)-4-hydroxyphenyl)ethan-1-aminium 2,2,2-trifluoroacetate (**3d**)

(24 mg, 0.045 mmol, 75% yield). ^1^H NMR (500 MHz, acetonitrile-*d*_3_) δ 7.98 (d, *J* = 9.2 Hz, 1H), 7.49 (d, *J* = 17.6 Hz, 1H), 7.41–7.31 (m, 5H), 7.13–7.00 (m, 1H), 6.85–6.65 (m, 5H), 5.35 (d, *J* = 14.6 Hz, 2H), 3.19 (s, 2H), 2.87 (d, *J* = 13.9 Hz, 2H), 2.44 (s, 3H); ^13^C NMR (126 MHz, acetonitrile-*d*_3_) δ 164.0, 159.4, 159.2, 150.0, 149.2, 147.3, 146.6, 146.0, 139.1, 134.1, 132.1, 129.5, 129.4, 128.0, 122.9, 120.3, 120.1, 118.7, 118.5, 116.2, 116.0, 115.6, 115.4, 95.0, 72.5, 41.4, 32.2, 20.3; HRMS (ESI-QTOF) *m*/*z*: [M – CF_3_CO_2_]^+^ calcd for C_26_H_23_N_3_O_3_, 426.1812; found, 426.1808.

#### 2-(3-((8-Cyano-7-hydroxy-4-(3,4,5-trimethoxyphenyl)quinolin-2-yl)methoxy)-4-hydroxyphenyl)ethan-1-aminium 2,2,2-trifluoroacetate (**3e**)

(29 mg, 0.047 mmol, 78% yield). ^1^H NMR (500 MHz, methanol-*d*_4_) δ 8.08 (d, *J* = 9.3 Hz, 1H), 7.63 (d, *J* = 16.2 Hz, 1H), 7.24 (dd, *J* = 9.4, 2.8 Hz, 1H), 7.07 (d, *J* = 1.9 Hz, 1H), 6.98 (d, *J* = 8.2 Hz, 1H), 6.84–6.73 (m, 4H), 5.37 (d, *J* = 24.2 Hz, 2H), 3.90–3.85 (m, 9H), 3.18–3.12 (m, 2H), 2.85 (dd, *J* = 16.1, 8.3 Hz, 2H); ^13^C NMR (126 MHz, methanol-*d*_4_) δ 164.0, 159.6, 153.3, 150.0, 149.0, 146.7, 145.9, 138.3, 132.9, 132.0, 130.2, 127.8, 121.9, 119.8, 119.7, 118.5, 118.3, 117.7, 117.6, 115.8, 114.5, 114.4, 106.9, 94.5, 71.7, 59.8, 55.5, 40.7, 32.7, 27.4; HRMS (ESI-QTOF) *m*/*z*: [M – CF_3_CO_2_]^+^ calcd for C_28_H_27_N_3_O_6_, 502.1973; found, 502.1978.

#### 2-(5-((8-Cyano-7-hydroxy-4-methoxyquinolin-2-yl)methoxy)-1H-indol-3-yl)ethan-1-aminium 2,2,2-trifluoroacetate (**4c**)

(19 mg, 0.037 mmol, 62% yield). ^1^H NMR (500 MHz, methanol-*d*_4_) δ 8.19 (d, *J* = 9.2 Hz, 1H), 7.30 (d, *J* = 8.8 Hz, 1H), 7.27–7.23 (m, 1H), 7.21–7.14 (m, 3H), 6.98–6.92 (m, 1H), 5.36 (s, 2H), 4.10 (s, 3H), 3.33 (s, 1H), 3.23 (t, *J* = 7.4 Hz, 2H), 3.08 (t, *J* = 7.4 Hz, 2H); ^13^C NMR (126 MHz, methanol-*d*_4_) δ 165.2, 164.8, 162.2, 160.2, 152.3, 147.7, 132.5, 128.3, 127.1, 124.0, 116.6, 114.4, 113.8, 112.2, 112.1, 112.0, 108.8, 101.3, 98.0, 92.5, 70.4, 55.9, 39.7, 23.1; HRMS (ESI-QTOF) *m*/*z*: [M – CF_3_CO_2_]^+^ calcd for C_22_H_20_N_4_O_3_, 389.1608; found, 389.1610.

#### (*R*)-7-Hydroxy-4-methoxy-2-(((6-(propyl(2-(thiophen-2-yl)ethyl)amino)-5,6,7,8-tetrahydronaphthalen-1-yl)oxy)methyl)quinoline-8-carbonitrile (**5c**)

(20 mg, 0.04 mmol, 65% yield). ^1^H NMR (500 MHz, chloroform-*d*) δ 8.49–8.28 (m, 1H), 7.47–7.12 (m, 4H), 7.09 (s, 1H), 7.01 (d, *J* = 5.0 Hz, 1H), 6.88 (d, *J* = 7.0 Hz, 2H), 5.40 (s, 2H), 4.25 (s, 2H), 3.88 (d, *J* = 12.3 Hz, 1H), 3.65 (dq, *J* = 15.6, 8.5 Hz, 1H), 3.60–3.11 (m, 10H), 2.90 (q, *J* = 12.5 Hz, 1H), 2.55–2.38 (m, 1H), 2.12–1.84 (m, 3H), 1.09 (t, *J* = 7.0 Hz, 3H); ^13^C NMR (126 MHz, chloroform-*d*) δ 163.3, 163.3, 162.4, 155.9, 149.6, 128.3, 126.6, 126.5, 125.2, 124.6, 123.3, 122.3, 115.9, 115.1, 113.9, 108.8, 99.1, 97.7, 95.0, 77.3, 71.1, 67.1, 60.4, 56.9, 56.0, 52.7, 32.1, 25.8, 24.1, 14.2, 11.9; HRMS (ESI-QTOF) *m*/*z*: [M + H]^+^ calcd for C_31_H_33_N_3_O_3_S, 528.2315; found, 528.2310.

#### *N*-(4-((8-Cyano-7-hydroxy-4-methoxyquinolin-2-yl)methoxy)-3-methoxybenzyl)nonanamide (**6c**)

(19 mg, 0.037 mmol, 62% yield). ^1^H NMR (500 MHz, acetone-*d*_6_) δ 8.12 (d, *J* = 9.2 Hz, 1H), 7.30 (s, 1H), 7.24 (d, *J* = 9.2 Hz, 1H), 7.14 (s, 1H), 6.96 (d, *J* = 8.1 Hz, 1H), 6.87 (d, *J* = 2.0 Hz, 1H), 6.68 (dd, *J* = 8.1, 2.1 Hz, 1H), 5.15 (s, 2H), 4.19 (d, *J* = 6.0 Hz, 2H), 3.98 (s, 3H), 3.75 (s, 3H), 2.08 (t, *J* = 7.4 Hz, 2H), 1.50–1.47 (m, 2H), 1.21–1.14 (m, 10H), 0.78–0.72 (m, 3H); ^13^C NMR (126 MHz, methanol-*d*_4_) δ 174.7, 163.6, 149.8, 147.0, 132.8, 128.8, 127.9, 122.3, 119.8, 114.5, 111.8, 109.8, 97.5, 72.0, 55.4, 55.2, 42.7, 42.4, 35.7, 31.6, 31.5, 29.0, 29.0, 28.9, 28.8, 25.7, 22.3, 13.0, 5.9; HRMS (ESI-QTOF) *m*/*z*: [M + H]^+^ calcd for C_29_H_35_N_3_O_5_, 506.2649; found, 506.2691.

#### 2-((4-Allyl-2-methoxyphenoxy)methyl)-7-hydroxy-4-methoxyquinoline-8-carbonitrile (**7c**)

(15 mg, 0.04 mmol, 67% yield). ^1^H NMR (500 MHz, methanol-*d*_4_) δ 8.24–8.15 (m, 1H), 7.20 (s, 1H), 7.17–7.11 (m, 1H), 7.01–6.95 (m, 1H), 6.84 (t, *J* = 2.7 Hz, 1H), 6.70 (dd, *J* = 8.1, 2.1 Hz, 1H), 5.96 (ddt, *J* = 16.8, 10.0, 6.7 Hz, 1H), 5.28 (s, 2H), 5.11–5.00 (m, 2H), 4.08 (s, 3H), 3.89 (d, *J* = 3.8 Hz, 3H), 3.32 (d, *J* = 1.7 Hz, 2H); ^13^C NMR (126 MHz, methanol-*d*_4_) δ 164.7, 164.1, 162.3, 149.6, 148.8, 146.1, 137.6, 134.3, 128.0, 120.5, 116.3, 114.7, 114.7, 114.5, 113.8, 112.6, 97.7, 93.3, 71.6, 55.6, 55.2, 39.4; HRMS (ESI-QTOF) *m*/*z*: [M + H]^+^ calcd for C_22_H_20_N_2_O_4_, 377.1496; found, 377.1491.

### Photochemistry

#### Measurement of UV Spectra and the Molar Extinction Coefficient (ε)

A 100 μM solution of each protected phenol was prepared in KMOPS buffer (20% CH_3_CN was used as a cosolvent for compounds **3d** and **6c**). The UV–vis spectra of these solutions were recorded at between 250 and 500 nm. All of the measurements were repeated in triplicate, and the absorbance values were averaged. The Beer–Lambert law, ε = *A*(*cl*)^−1^, was used for the calculation of ε values at λ = 365 nm, where *A* is the absorbance value measured at 365 nm, *c* is the concentration of the sample, and *l* is the cuvette length (1 cm). See the Supporting Information for the spectra.

#### Spontaneous Hydrolysis in the Dark

A 0.1 mM solution of each compound was prepared in KMOPS buffer. For compounds **3d** and **6c**, 20% CH_3_CN was used as a cosolvent. The solutions were kept in the dark for 7 days, and the percentage of the starting materials was determined by periodic HPLC analysis (Supporting Information, HPLC Data). Negligible to no hydrolysis was observed for a period of 7 days for all of the compounds.

#### One-Photon Excitation (1PE)

A 0.1 mM solution (3 mL) of each protected phenol in KMOPS buffer (10 or 20% CH_3_CN was used as a cosolvent for **3d** and **6c**) was placed in a 3 mL quartz cuvette along with a stirring bar. The solution was irradiated with stirring using a 365 nm LED lamp (Cairn OptoLED Lite). At different time intervals, 80 μL aliquots of the solution were taken out and analyzed by uHPLC (Agilent 1290 Infinity series), monitoring the AUC at 320 nm (Supporting Information, HPLC Data). An external standard calibration method of quantification was used for the calibration of the samples. The experiments were repeated in sets of three. A gradient elution with a flux rate of 0.3 mL/min using a mobile phase comprising A = 0.1% trifluoroacetic acid in water and B = acetonitrile (starting from 5% B to 100% over 10 min and re-equilibrating to 5% B before the next run) was used for the separations. A comparison of the AUC with the calibration curves of the substrates (obtained from known concentration) determined the percentages of the remaining starting materials. The percentages of the remaining starting materials were plotted versus time. The time to 90% of decomposition of the substrate (*t*_90%_ values) was obtained by fitting a single exponential decay curve to the data using DeltaGraph (Red Rock Software). The following equation was used to calculate the quantum yield (Φ_u_) of each photolysis reaction:^42,47,56,57^ Φ_u_ = (*I* σ *t*_90%_)^−1^, where *I* is the irradiation intensity (determined by potassium ferrioxalate actinometry and measured in Einstein cm^–2^ s^–1^),^58^ σ is the decadic extinction coefficient (1000 × ε) at 365 nm, and *t*_90%_ is the time required to consume 90% of the starting material. See Supporting Information Table S1 for the *I*, σ, and *t*_90%_ data.

#### Two-Photon Excitation (2PE)

A 50–100 μM solution (25 μL) of each protected phenol was placed in a microcuvette (26.10F-Q-10, Starna, 10 × 1 × 1 mm illuminated dimensions). The solution was irradiated for different time intervals (typically 5, 10, and 30 min) with 740 nm light from a femtosecond-pulsed and mode-locked Ti:sapphire laser (Mai Tai HP DeepSee, Spectra-Physics) focused on the center of the cuvette chamber. Average power of 450 to 650 mW of the laser was used for the photolysis. The quantification of the samples was carried out the same way as in 1PE experiments (Supporting Information, HPLC Data). DeltaGraph (Red Rock Software) was used to plot the data to fit to a single exponential decay. The following equation was used to calculate the 2-photon uncaging action cross section (δ_u_) in Goeppert Mayer (GM, 10^–50^ cm^4^·s/photon) using fluorescein as the external standard:^42−44^
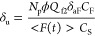
*N*_p_ = number of molecules photolyzed per second determined by HPLC analysis.

*Q*_f2_ = fluorescence quantum yield of the external standard fluorescein (0.9).^59,60^

δ_aF_ = 2-photon absorption cross section of fluorescein (30 GM at 740 nm).^61^

*C*_F_ = concentration of fluorescein.

<*F*(*t*)> = time-averaged fluorescence photon flux of the fluorescein standard measured by a radiometer positioned at a right angle to the excitation laser beam (photons/s).

*C*_s_ = concentration of the sample being photolyzed.

φ = estimated collection efficiency of the fluorescence detector, calculated according to the following equation:
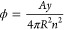
*A* = area of detector (0.33 cm^2^).

*y* = fraction of integrated emission spectrum transmitted by interference filter (0.465).

*R* = distance from the center of the cuvette to the detector.

*n* = refractive index of water (1.33).

See Supporting Information Table S2 for *C*_F_, *C*_S_, *R*, *N*_p_, ϕ, and <*F*(*t*)> data.

### Biology

#### General

All of the required materials for cell culturing were purchased from commercial sources and used without further purification.

#### Plasmids

Plasmids were purchased from Addgene. The catalog number for the dLight 1.2 plasmid is 111054, and that for the GRAB5-HT is 180005.

#### Cell Culture

HCT 116 cells were grown in Dulbecco’s minimal essential medium (DMEM) supplemented with 10% fetal bovine serum (FBS), 1% nonessential amino acids (NEAA), and maintained at 37 °C and 5% CO_2_. HEK 293 cells stably expressing TRPV1 channels were grown as previously described.^62^

#### Calcium Dye Loading

HEK 293 cells (stably expressing TRPV1 channels) were plated on a 35 mm glass-bottom dish (previously coated with poly-d-Lysine) a day before the experiment. A mixture of pluronic F-127 (Molecular Probes) and Fluo-4 AM (50 μg, Life Technologies) dissolved in dimethylsulfoxide (DMSO) (50 μL) was added to Hank’s balanced salt solution (HBSS) and sonicated for 5 min to prepare a solution containing a 0.02% final concentration of each. The solution was then loaded onto the cells growing in the 35 mm glass-bottom dish and incubated for 35–45 min in a humidified CO_2_ incubator (37 °C, 5% CO_2_). The cells were washed with HBSS (3 × 2 mL each) and subsequently maintained in HBSS during photoactivation experiments.

#### Imaging

An Olympus FluoView FV1000MPE confocal microscope was employed to image and record cells plated onto 35 mm glass-bottom 4-well plates. A 488 nm ion laser excited the dopamine and serotonin sensors dLight 1.2 and GRAB5-HT, respectively, and Fluo-4 calcium dye. FluoView 10-ASW version 4.0 (Olympus), ImageJ-Fiji, and DeltaGraph (Red Rock Software) were used for capturing, extracting, and processing the collected data. Photoactivation was conducted with 405 nm (100% laser power, 1PE) or 720 nm (10% laser power, 24 mW, 2PE) light for MeO-CyHQ-O-DA and MeO-CyHQ-O-5HT or 740 nm (20% laser power, 15 mW light) for MeO-CyHQ-O-VNA and MeO-CyHQ-O-EG. Images were captured by using a 60× immersion lens.

#### Activation of dLight 1.2 or GRAB5-HT on HCT 116 Cells

HCT 116 cells were grown to 70% confluency on specialized glass-bottom plates for imaging. After 24 h, the cells were transfected (70% efficiency) with either dLight 1.2 or GRAB5-HT. Before imaging, the DMEM was discarded and the cells were washed twice with HBSS. HBSS (500 μL) was added per well for imaging. DMSO was used to dissolve the compounds CyHQ-O-DA, MeO-CyHQ-O-DA, and MeO-CyHQ-O-5HT to make 1 mM stock solutions. These were then added to the wells to obtain the desired final concentrations, keeping the DMSO concentration to less than 1%, which is tolerated by the cells. Control experiments included optimization of the response of the dLight 1.2 and GRAB5-HT sensors to the release of dopamine and serotonin, respectively, through exposure to different concentrations of CyHQ-O-DA, MeO-CyHQ-O-DA, and MeO-CyHQ-O-5HT at different durations of 405 nm light. Optimizations were conducted in a similar manner for activation through 2PE. Light dose–response curves were generated by applying a 405 nm light pulse for 1, 10, 50, 100, 200, 300, 400, 500, 600, 750, and 1000 ms at 50 frame intervals for CyHQ-O-DA, MeO-CyHQ-O-DA, and MeO-CyHQ-O-5HT.

#### TRPV1 Activation on HEK 293 Cells

Solutions of MeO-CyHQ-O-VNA or MeO-CyHQ-O-EG were added to HEK 293 cells stably expressing TRPV1 channels and loaded with Fluo-4 in a 35 mm glass-bottom dish. An area near a cell was irradiated with a single short pulse of 405 nm laser light while imaging with the 488 nm laser. Similarly, VNA and eugenol were released through 2PE using 740 nm light.

## Supporting Information Available

Data for calculation of the photolysis reaction quantum yield and 2-photon uncaging action cross sections (δ_u_) at 740 and 720 nm; UV–vis spectra; NMR spectra; HPLC chromatograms; and figures, tables, and video descriptions for dopamine, serotonin, VNA, and eugenol release in cell culture (PDF)
Videos S1−S22 (ZIP)
