# Supplementary material for: Multi-Photon-Sensitive Chromophore for the Photorelease of Biologically Active Phenols
Source: ACS Chem Neurosci. 2023 Nov 21;14(23):4163–75. doi: 10.1021/acschemneuro.3c00552 (PMC10704561; doi:10.1021/acschemneuro.3c00552)

Supporting Information

A Multi-Photon Sensitive Chromophore for the Photorelease of Biologically Active Phenols

Naeem Asad,[†,§] Davide Deodato,[†,§] Nadeem Asad,[†,§] Sangram Gore,[†] and Timothy M. Dore*[,†,‡]

[†]New York University Abu Dhabi, PO Box 129188, Abu Dhabi, United Arab Emirates
[‡]Department of Chemistry, University of Georgia, Athens, GA 30602, USA

[§]These authors contributed equally.
*To whom correspondence should be addressed:
tdore@uga.edu

Table of Contents

Tables S3
Table S1. Calculation of the photolysis reaction quantum yield S3
Table S2. Data for the calculation of 2-photon uncaging action cross-sections (δ_u) S3

Figures S4
Figure S1. Control experiments for dopamine and serotonin. S5
Figure S2. Dopamine released in a controlled spatial and temporal manner from CyHQ-O-DA as depicted by the activation of dLight 1.2 expressed on the membrane of HCT 116 cells using 1PE (405 nm). S7
Figure S3. Dopamine released in a controlled spatial and temporal manner from MeO-CyHQ-O-DA as depicted by the activation of dLight 1.2 expressed on the membrane of HCT 116 cells using 1PE (405 nm). S9
Figure S4. Serotonin released in a controlled spatial and temporal manner from MeO-CyHQ-O-5HT as depicted by the activation of 5-HT (GRAB5-HT) sensor expressed on the membrane of HCT 116 cells using 1PE (405 nm). S10
Figure S5. Calcium influx measured in HEK 293 cells stably expressing TRPV1 channels after activation of MeO-CyHQ-VNA (2.5 μM) with a 50-ms flash of 405-nm light. S11
Figure S6. Calcium influx measured in HEK 293 cells stably expressing TRPV1 channels after activation of MeO-CyHQ-EG (5 μM) with a 500-ms flash of 405-nm light. S12

Video Descriptions S13

UV-Vis Spectra S14

NMR Spectra S17
(8-cyano-7-(methoxymethoxy)-4-methylquinolin-2-yl)methyl methanesulfonate (**2a**) S17
(8-cyano-7-(methoxymethoxy)-4-morpholinoquinolin-2-yl)methyl methanesulfonate (**2b**) S18
(8-Cyano-4-methoxy-7-(methoxymethoxy)quinolin-2-yl)methyl methanesulfonate (**2c**) S19
(8-cyano-7-(methoxymethoxy)-4-(p-tolyl)quinolin-2-yl)methyl methanesulfonate (**2d**) S20
(8-cyano-7-(methoxymethoxy)-4-(3,4,5-trimethoxyphenyl)quinolin-2-yl)methyl methanesulfonate (**2e**) S21
2-(3-((8-cyano-7-hydroxy-4-methylquinolin-2-yl)methoxy)-4-hydroxyphenyl)ethan-1-aminium 2,2,2-trifluoroacetate (**3a**) S22
2-(3-((8-cyano-7-hydroxy-4-morpholinoquinolin-2-yl)methoxy)-4-hydroxyphenyl)ethan-1-aminium 2,2,2-trifluoroacetate (**3b**) S23

2-(3-((8-cyano-7-hydroxy-4-methylquinolin-2-yl)methoxy)-4-hydroxyphenyl)ethan-1-aminium 2,2,2-trifluoroacetate **(3c)** S24
2-(3-((8-cyano-7-hydroxy-4-(p-tolyl)quinolin-2-yl)methoxy)-4-hydroxyphenyl)ethan-1-aminium 2,2,2-trifluoroacetate **(3d)** S25
2-(3-((8-cyano-7-hydroxy-4-(2,4,6-trimethoxyphenyl)quinolin-2-yl)methoxy)-4-hydroxyphenyl)ethan-1-aminium 2,2,2-trifluoroacetate **(3e)** S26
2-(5-((8-cyano-7-hydroxy-4-methoxyquinolin-2-yl)methoxy)-1H-indol-3-yl)ethan-1-aminium 2,2,2-trifluoroacetate **(4c)** S27
(R)-7-hydroxy-4-methoxy-2-(((6-(propyl(2-(thiophen-2-yl)ethyl)amino)-5,6,7,8-tetrahydronaphthalen-1-yl)oxy)methyl)quinoline-8-carbonitrile **(5c)** S28
N-(4-((8-cyano-7-hydroxy-4-methoxyquinolin-2-yl)methoxy)-3-methoxybenzyl)nonanamide **(6c)** S29
2-((4-allyl-2-methoxyphenoxy)methyl)-7-hydroxy-4-methoxyquinoline-8-carbonitrile **(7c)** S30

HPLC Data S31
Me-CyHQ-DA (**3a**) S32
Mor-CyHQ-DA (**3b**) S35
MeO-CyHQ-DA (**3c**) S38
pTol-CyHQ-DA (**3d**) S42
TMP-CyHQ-DA (**3e**) S45
MeO-CyHQ-5HT (**4c**) S48
MeO-CyHQ-RTG (**5c**) S52
MeO-CyHQ-VNA (**6c**) S54
MeO-CyHQ-EG (**7c**) S58

Tables

Table S1. Calculation of the photolysis reaction quantum yield

Compound	I (Einstein cm^{-2} s^{-1}) 365 nm	σ (cm^2 mol^{-1}) 365 nm	$t_{90\%}$ (s) 365 nm
(Me-CyHQ-O-DA) (**3a**)	$2.89 \cdot 10^{-8}$	$5.01 \cdot 10^{6}$	28.0
(Mor-CyHQ-O-DA) (**3b**)	$2.49 \cdot 10^{-8}$	$4.90 \cdot 10^{6}$	23.2
(MeO-CyHQ-O-DA) (**3c**)	$1.92 \cdot 10^{-8}$	$5.67 \cdot 10^{6}$	25.4
(pTol-CyHQ-O-DA) (**3d**)	$3.86 \cdot 10^{-8}$	$4.80 \cdot 10^{6}$	76.7
(TMP-CyHQ-O-DA) (**3e**)	$3.74 \cdot 10^{-8}$	$5.21 \cdot 10^{6}$	188.7
(MeO-CyHQ-O-5HT) (**4c**)	$2.50 \cdot 10^{-8}$	$5.40 \cdot 10^{6}$	18.4
(MeO-CyHQ-O-RTG) (**5c**)	$2.50 \cdot 10^{-8}$	$2.30 \cdot 10^{6}$	143.9
(MeO-CyHQ-O-VNA) (**6c**)	$2.50 \cdot 10^{-8}$	$3.37 \cdot 10^{6}$	23.1
(MeO-CyHQ-O-EG) (**7c**)	$2.50 \cdot 10^{-8}$	$7.54 \cdot 10^{6}$	19.5

Table S2. Data for the calculation of 2-photon uncaging action cross-sections (δ_u)

2PE at 740 nm

Compound	C_F (μM)	C_S (μM)	R (cm)	N_p	φ	<F(t)> (photons/s)
(Me-CyHQ-O-DA) (**3a**)	12.9	100	2.86	$7.68 \cdot 10^{10}$	$8.40 \cdot 10^{-4}$	$4.44 \cdot 10^{8}$
(Mor-CyHQ-O-DA) (**3b**)	12.9	100	2.86	$5.85 \cdot 10^{10}$	$8.40 \cdot 10^{-4}$	$3.91 \cdot 10^{8}$
(MeO-CyHQ-O-DA) (**3c**)	12.9	100	2.86	$7.79 \cdot 10^{10}$	$8.40 \cdot 10^{-4}$	$2.66 \cdot 10^{8}$
(pTol-CyHQ-O-DA) (**3d**)	11.5	85	2.86	$6.76 \cdot 10^{10}$	$8.40 \cdot 10^{-4}$	$3.91 \cdot 10^{8}$
(MeO-CyHQ-O-5HT) (**4c**)	12.9	100	2.86	$1.84 \cdot 10^{11}$	$8.40 \cdot 10^{-4}$	$5.33 \cdot 10^{8}$
(MeO-CyHQ-O-VNA) (**6c**)	12.9	50	2.86	$5.85 \cdot 10^{10}$	$8.40 \cdot 10^{-4}$	$3.99 \cdot 10^{8}$
(MeO-CyHQ-O-EG) (**7c**)	12.9	100	2.86	$1.60 \cdot 10^{11}$	$8.40 \cdot 10^{-4}$	$6.21 \cdot 10^{8}$

2PE at 720 nm

Compound	C_F (μM)	C_S (μM)	R (cm)	N_p	φ	<F(t)> (photons/s)
(MeO-CyHQ-O-DA) (**3c**)	12.9	100	2.86	$9.46 \cdot 10^{10}$	$8.40 \cdot 10^{-4}$	$1.86 \cdot 10^{8}$
(MeO-CyHQ-O-5HT) (**4c**)	12.9	100	2.86	$2.45 \cdot 10^{11}$	$8.40 \cdot 10^{-4}$	$4.44 \cdot 10^{8}$
(MeO-CyHQ-O-VNA) (**6c**)	12.9	50	2.86	$1.03 \cdot 10^{11}$	$8.40 \cdot 10^{-4}$	$4.44 \cdot 10^{8}$
(MeO-CyHQ-O-EG) (**7c**)	12.9	100	2.86	$2.19 \cdot 10^{11}$	$8.40 \cdot 10^{-4}$	$4.88 \cdot 10^{8}$

Figures

(A)

Pulse Duration (ms)	0	0	0
Dopamine	0	100μM	0
Time Point (s)	1.11	24	112
Images	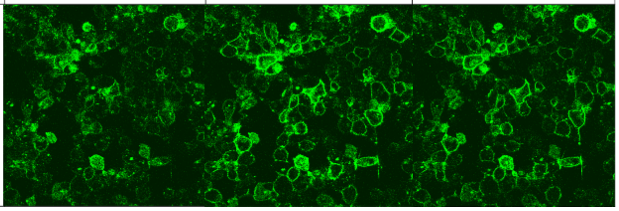		

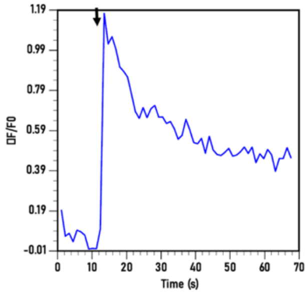

(B)

Pulse Duration (ms)	0	0	0
Serotonin	0	100μM	0
Time Point (s)	1.11	24	112
Images	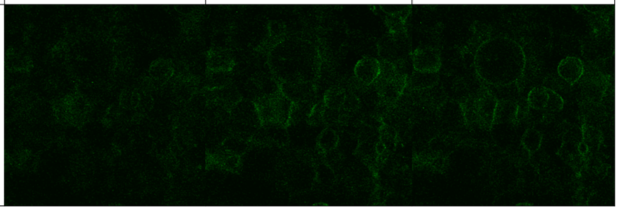		

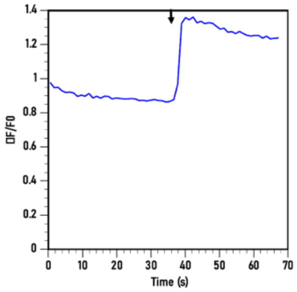

(C)

Pulse Duration (ms)	Continuous 1000	Continuous 1000	Continuous 1000
Time Point (s)	1.11	61	112
Images	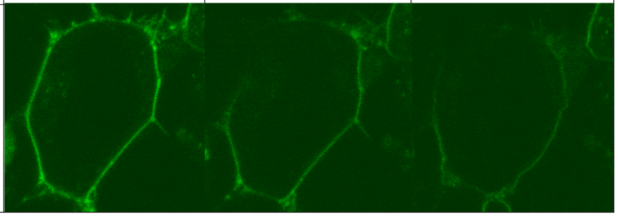		

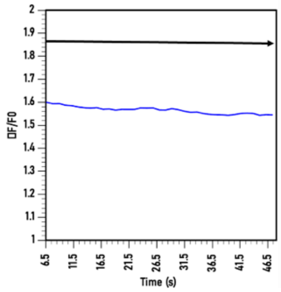

(D)

Pulse Duration (ms)	0	10	0
Time Point (s)	1.11	56	112
Images	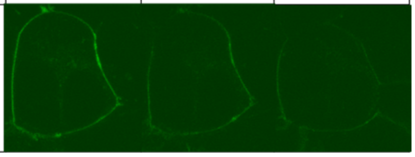		

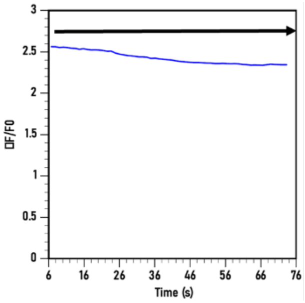

(E)

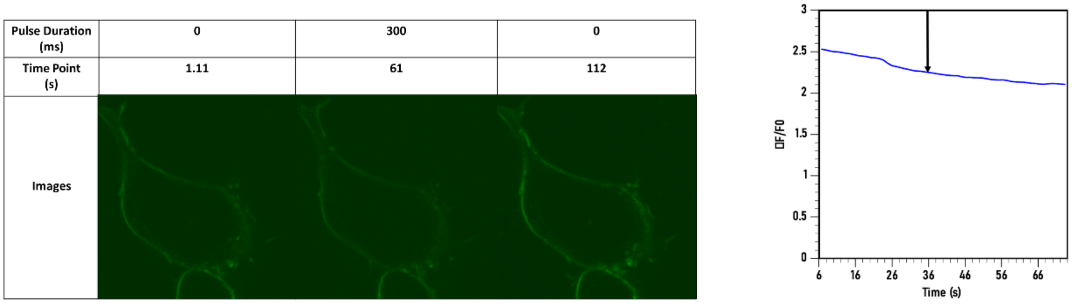

(F)

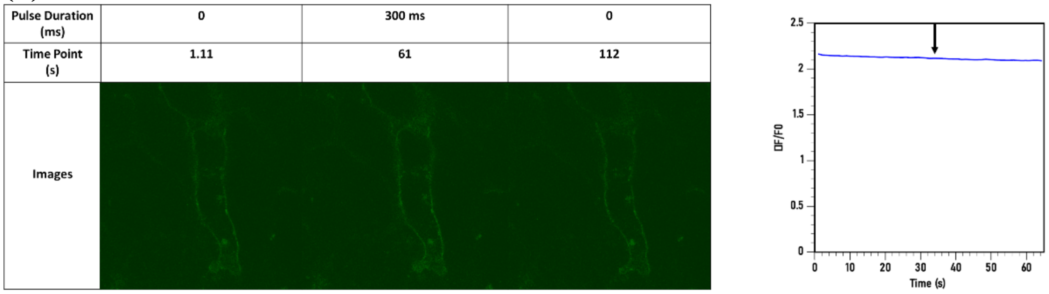

Figure S1. Control experiments for dopamine and serotonin.

(A) Response of dLight 1.2 expressed on the membrane of HCT 116 cells in HBSS media to bulk addition of dopamine (100 μM) by a micropipette into the glass-bottomed, 4-well culture plate (see Video S3). (B) Response of GRAB5-HT expressed on the membrane of HCT 116 cells in HBSS media to bulk addition of serotonin (100 μM) by a micropipette into the glass-bottomed, 4-well culture plate (see Video S4). (C and D) No response of dLight 1.2 (C) or GRAB5-HT (D) to a continuous 1000-ms, 405-nm pulse of light (1PE) in the absence of photoactivatable dopamine or serotonin, respectively (see Video S5 and S6). (E and F) No response of dLight 1.2 (E) or GRAB5-HT (F) to a 300-ms, 720-nm laser light pulse (2PE) in the absence of MeO-CyHQ-O-DA or MeO-CyHQ-O-5HT, respectively, depicting that dLight 1.2 and GRAB5-HT do not fluoresce with light exposure in the absence of dopamine and serotonin (see Video S7 and S8).

(A)

Pulse Duration (ms)	0	1	10	50	100	200	300
Time Point (s)	1.11	55	111	166	222	277	333
Images	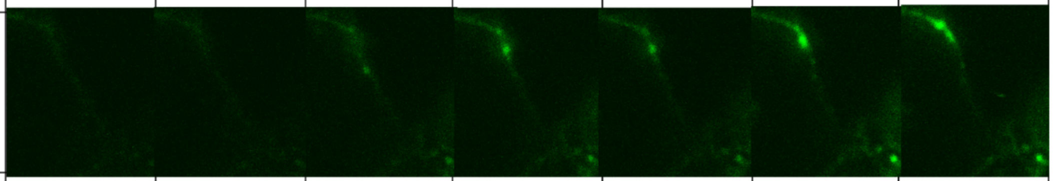						
Pulse Duration (ms)	400	500	600	750	1000	0	0
Time Point (s)	387	444	499	555	610	666	666
Images	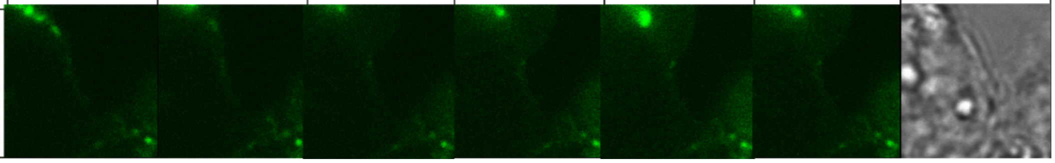						

(B)

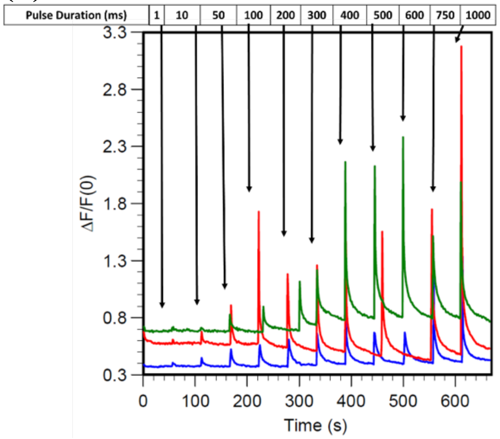

(C)

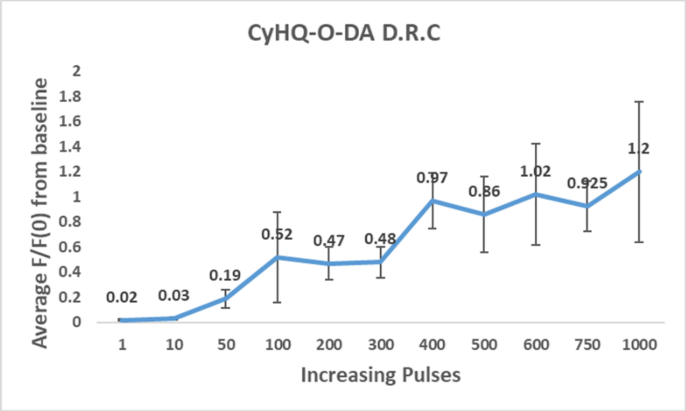

(D)

Pulse Duration (ms)	0	Pulse	0	0
Time Point (s)	1.11	56	112	112
50 µM Concentration CyHQ-O-DA 50 millisecond Pulse	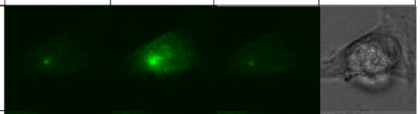			
20 µM Concentration CyHQ-O-DA 100 millisecond Pulse	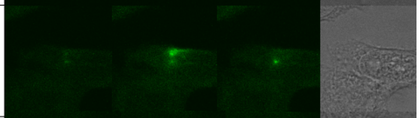			
100 µM Concentration CyHQ-O-DA 10 millisecond Pulse	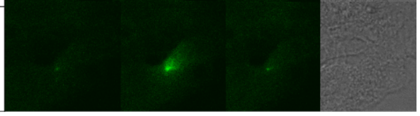			

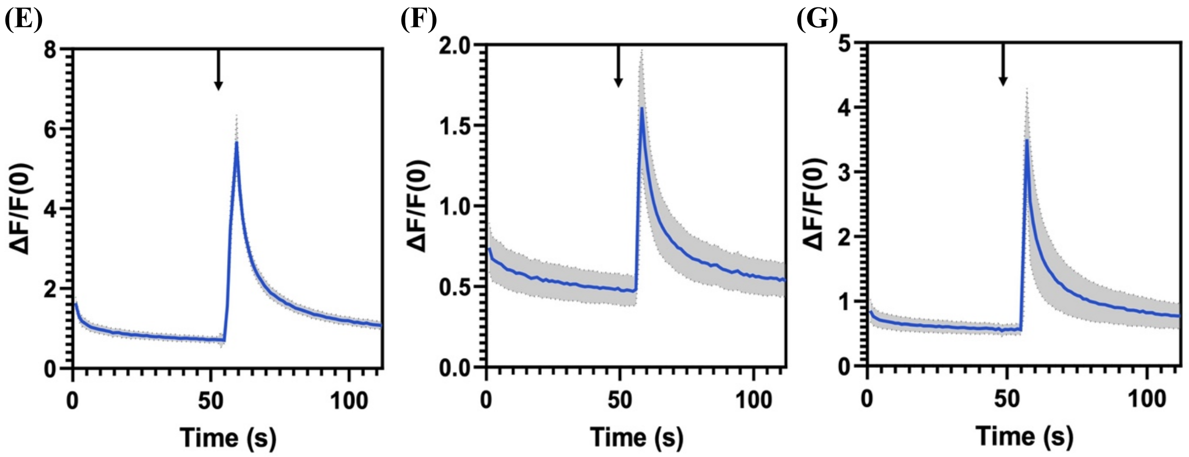

Figure S2. Dopamine released in a controlled spatial and temporal manner from CyHQ-O-DA as depicted by the activation of dLight 1.2 expressed on the membrane of HCT 116 cells using 1PE (405 nm).

(A) Light dose-response mediated by CyHQ-O-DA (100 μM) with increasing pulse durations (1, 10, 50, 100, 200, 300, 400, 500, 600, 750, 1000 ms) after every 50 frames. (see Video S9). (B) Plot of $\Delta F/F_0$ vs. time (s) for CyHQ-O-DA (100 μM) measured in 3 experiments. Arrows mark the time point of the light pulse. (C) Plot of average $\Delta F/F_0$ after baseline subtraction vs. pulse duration. (D) Response of dLight 1.2 to a single, 50, 100, and 10-ms pulse of 405-nm light to activate CyHQ-O-DA at a concentration of 50, 20, and 100 μM, respectively (see Videos S10-S12). (E-G) Plot of average $\Delta F/F_0$ vs time (s) for CyHQ-O-DA (50, 20, and 100 μM) from 3 experiments for each treatment at 50, 100, and 10-ms pulses, respectively. Gray areas show the standard deviation of the measurement. Arrow marks the time point of the light pulse.

(A)

Pulse Duration (ms)	0	1	10	50	100	200	300
Time Point (s)	1.11	55	111	166	222	277	333
Images	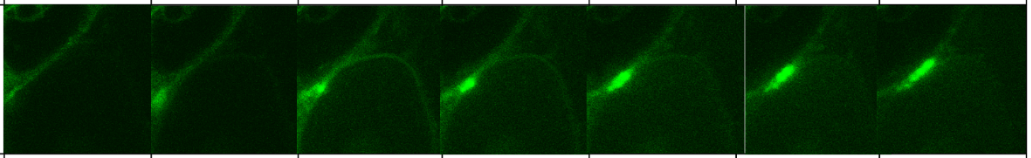						
Pulse Duration (ms)	400	500	600	750	1000	0	0
Time Point (s)	387	444	499	555	610	666	666
Images	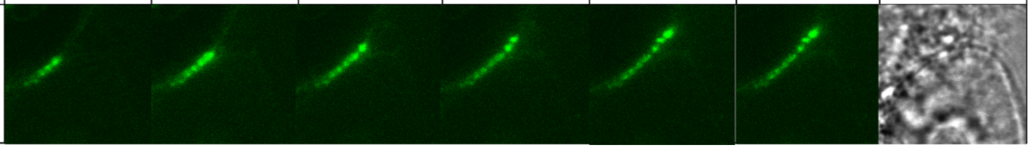						

(B)

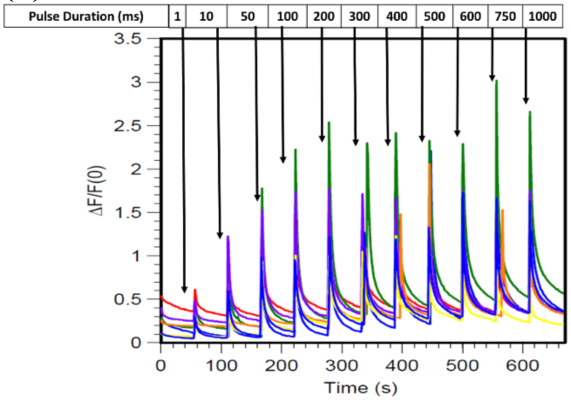

(C)

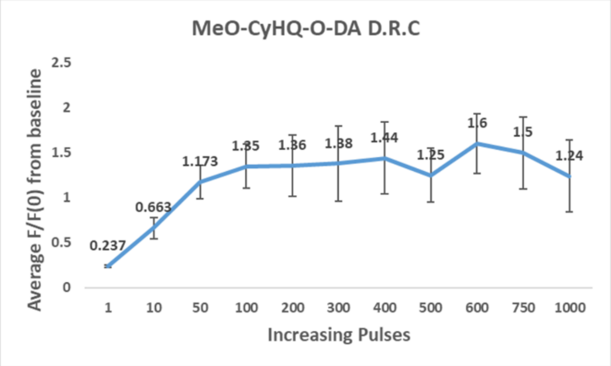

(D)

Pulse Duration (ms)	0	10	0	0
Time Point (s)	1.11	56	112	112
Images	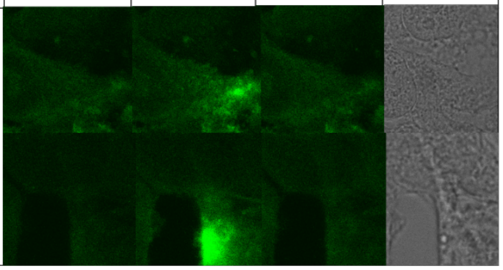			

(E)

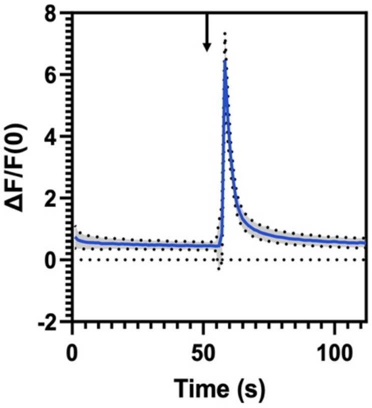

(F)

Pulse Duration (ms)	0	1	10	50	100	200	300
Time Point (s)	1.11	55	111	166	222	277	333
Images	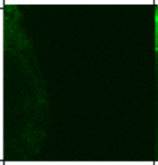	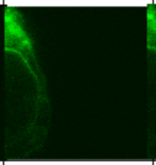	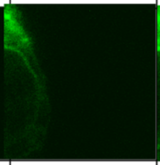	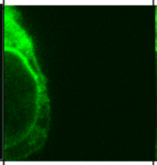	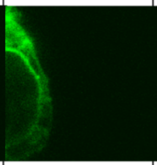	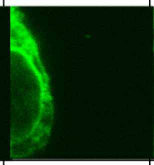	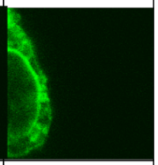
Pulse Duration (ms)	400	500	600	750	1000	0	0
Time Point (s)	387	444	499	555	610	666	666
Images	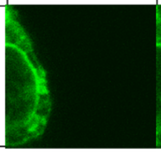	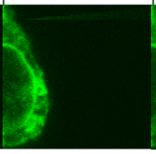	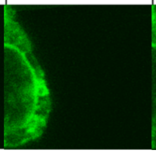	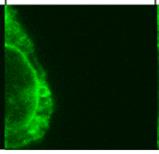	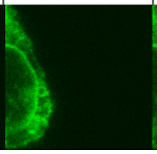	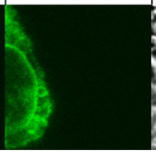	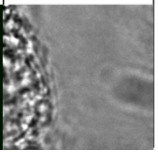

Figure S3. Dopamine released in a controlled spatial and temporal manner from MeO-CyHQ-O-DA as depicted by the activation of dLight 1.2 expressed on the membrane of HCT 116 cells using 1PE (405 nm).
(A) Light dose-response mediated by MeO-CyHQ-O-DA (10 μM) with increasing pulse durations (1, 10, 50, 100, 200, 300, 400, 500, 600, 750, 1000 ms) after every 50 frames at different points on the cell (see Video S13). (B) Plot of $\Delta F/F_0$ vs. time (s) for MeO-CyHQ-O-DA (100 μM) measured in 7 experiments. Arrows mark the time point of the light pulse. (C) Plot of average $\Delta F/F_0$ after baseline subtraction vs. pulse duration (ms). (D) Response of dLight 1.2 to a single 10-ms pulse of 405-nm light to activate MeO-CyHQ-O-DA (10 μM) (see Videos S14-S15). (E) Plot of average $\Delta F/F_0$ vs time (s) for CyHQ-O-DA (10 μM) from 5 experiments. Gray areas show the standard deviation of the measurement. Arrow marks the time point of the 10-ms light pulse. (F) Light dose-response mediated by MeO-CyHQ-O-DA (10 μM) with increasing pulse durations (1, 10, 50, 100, 200, 300, 400, 500, 600, 750, 1000 ms) after every 50 frames at the same point on the cell (see Video S16).

(A)

Pulse Duration (ms)	0	1	10	50	100	200	300
Time Point (s)	1.11	55	111	166	222	277	333
Images	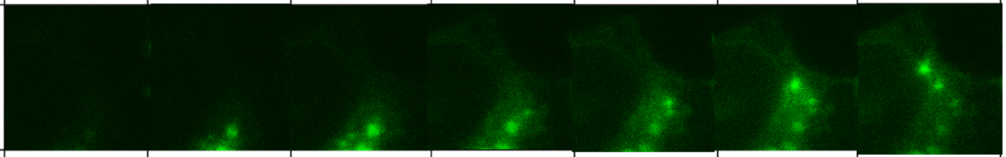						
Pulse Duration (ms)	400	500	600	750	1000	0	0
Time Point (s)	387	444	499	555	610	666	666
Images	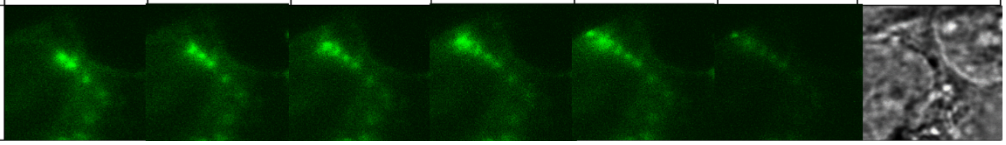						

(B)

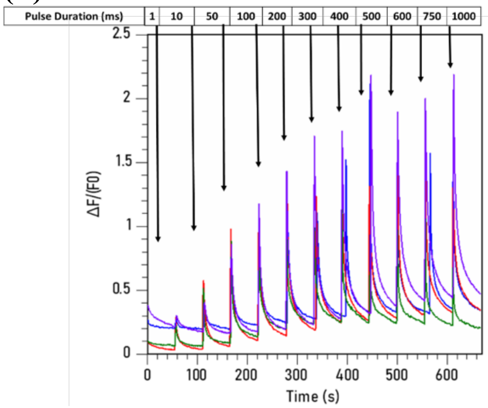

(C)

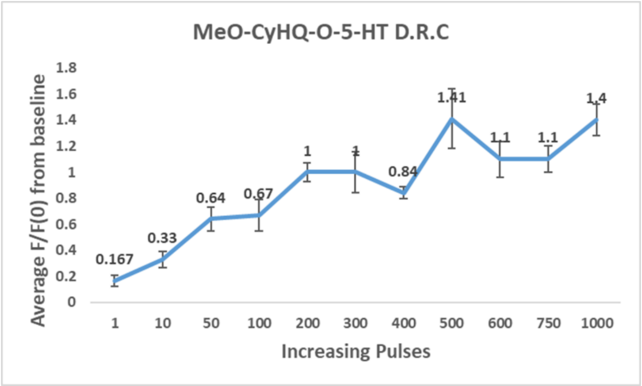

(D)

Pulse Duration (ms)	0	20	0	0
Time Point (s)	1.11	56	112	112
Images	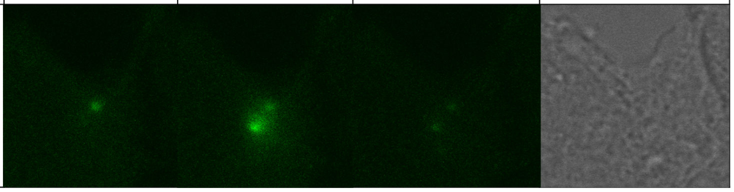			

(E)

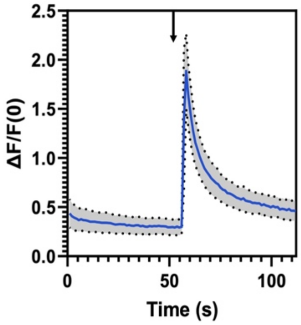

Figure S4. Serotonin released in a controlled spatial and temporal manner from MeO-CyHQ-O-5HT as depicted by the activation of 5-HT (GRAB5-HT) sensor expressed on the membrane of HCT 116 cells using 1PE (405 nm).
(A) Light dose-response mediated by MeO-CyHQ-O-5HT (20 µM) with increasing pulse durations (1, 10, 50, 100, 200, 300, 400, 500, 600, 750, 1000 ms) after every 50 frames (see Video S17). (B) Plot of $\Delta F/F_0$ vs. time (s) for MeO-CyHQ-O-5HT (20 µM) measured in 4 experiments. Arrows mark the time point of the light pulse. (C) Plot of average $\Delta F/F_0$ after baseline subtraction vs. pulse duration (ms). (D) Response of GRAB5-HT to a single 20-ms pulse of 405-nm light to activate MeO-CyHQ-O-5HT (20

μM) (see Video S18). (E) Plot of average $\Delta F/F_0$ vs time (s) for MeO-CyHQ-O-5HT (20 μM) from 4 experiments. Gray areas show the standard deviation of the measurement. Arrow marks the time point of the light pulse.

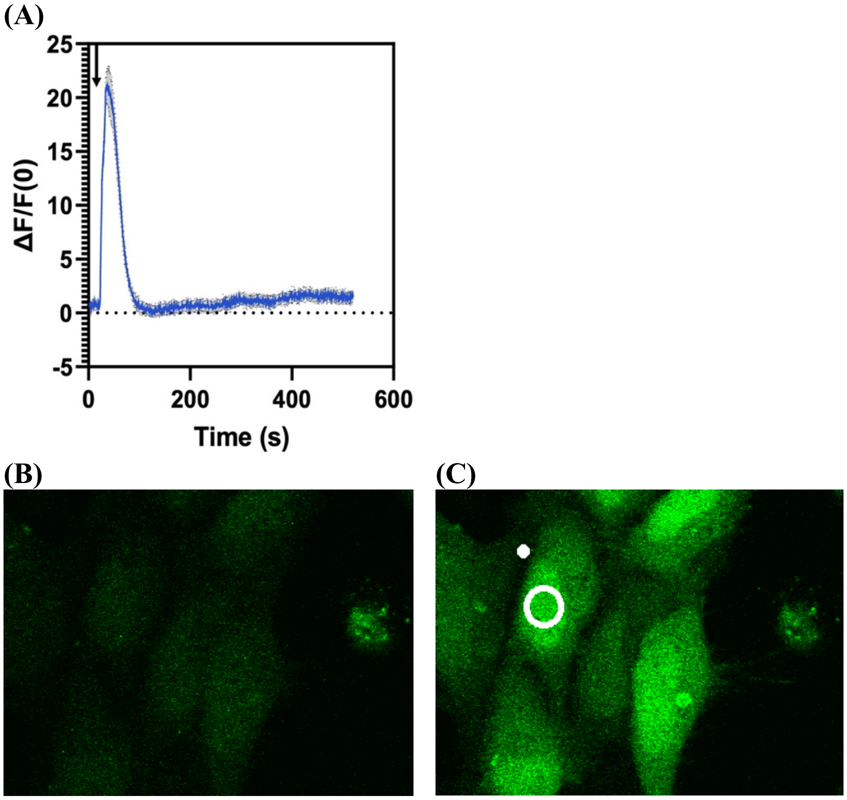

Figure S5. Calcium influx measured in HEK 293 cells stably expressing TRPV1 channels after activation of MeO-CyHQ-VNA (2.5 μM) with a 50-ms flash of 405-nm light.
(A) Plot of $\Delta F/F_0$ vs. time (s). The graph is the average of three different experiments. Gray areas show the standard deviation of the measurement. Black arrow indicates the timing of the light flash at the 21-s time point. (B) Image of cells before light exposure. (C) Image of cells at peak fluorescent Ca^{2+} indicator response (see Video S21).

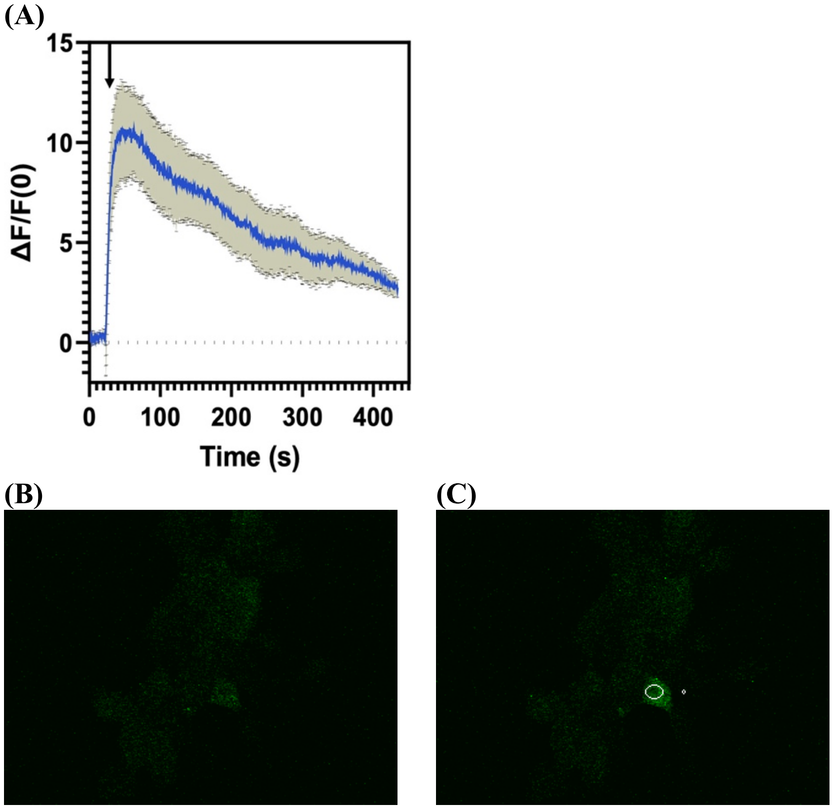

Figure S6. Calcium influx measured in HEK 293 cells stably expressing TRPV1 channels after activation of MeO-CyHQ-EG (5 µM) with a 500-ms flash of 405-nm light.
(A) Plot of $\Delta F/F_0$ vs. time (s). The graph is an average of three different experiments. Beige areas show the standard deviation of the measurement. Black arrow indicates the timing of the light flash at the 22-s time point). (B) Image of the cells before light exposure. (C) Image of the cells at peak fluorescent Ca^{2+} indicator response (see Video S22).

Video Descriptions

Video S1: Response of dLight 1.2 to a single, 200-ms pulse of 720-nm light to activate MeO-CyHQ-O-DA (100 μM).

Video S2: Response of GRAB5-HT to a single, 200-ms pulse of 720-nm light to activate MeO-CyHQ-O-5HT (200 μM).

Video S3: Response of dLight 1.2 expressed on the membrane of HCT 116 cells in HBSS media to bulk addition of dopamine (100 μM) by a micropipette into the glass-bottomed, 4-well culture plate.

Video S4: Response of GRAB5-HT expressed on the membrane of HCT 116 cells in HBSS media to bulk addition of serotonin (100 μM) by a micropipette into the glass-bottomed, 4-well culture plate

Video S5: No response of dLight 1.2 to a continuous 1000-ms, 405-nm pulse of light (1PE) in the absence of MeO-CyHQ-O-DA.

Video S6: No response of GRAB5-HT to a continuous 1000-ms, 405-nm pulse of light (1PE) in the absence of MeO-CyHQ-O-5HT.

Video S7: No response of dLight 1.2 to a 300-ms, 720-nm laser light pulse (2PE) in the absence of MeO-CyHQ-O-DA.

Video S8: No response of GRAB5-HT to a 300-ms, 720-nm laser light pulse (2PE) in the absence of MeO-CyHQ-O-5H-.

Video S9: Light dose-response mediated by CyHQ-O-DA (100 μM) with increasing pulse durations (1, 10, 50, 100, 200, 300, 400, 500, 600, 750, 1000 ms) after every 50 frames.

Video S10: Response of dLight 1.2 to a single, 50-ms pulse of 405-nm light to activate CyHQ-O-DA (50 μM).

Video S11: Response of dLight 1.2 to a single, 100-ms pulse of 405-nm light to activate CyHQ-O-DA (20 μM).

Video S12: Response of dLight 1.2 to a single, 10-ms pulse of 405-nm light to activate CyHQ-O-DA (100 μM).

Video S13: Light dose-response mediated by MeO-CyHQ-O-DA (10 μM) with increasing pulse durations (1, 10, 50, 100, 200, 300, 400, 500, 600, 750, 1000 ms) after every 50 frames at different points on the cell.

Video S14: Response of dLight 1.2 to a single 10-ms pulse of 405-nm light to activate MeO-CyHQ-O-DA at a concentration of 10 μM respectively.

Video S15: Response of dLight 1.2 to a single 10-ms pulse of 405-nm light to activate MeO-CyHQ-O-DA at a concentration of 10 μM respectively.

Video S16: Light dose-response mediated by MeO-CyHQ-O-DA (10 μM) with increasing pulse durations (1, 10, 50, 100, 200, 300, 400, 500, 600, 750, 1000 ms) after every 50 frames at the same point on the cell.

Video S17: Light dose-response mediated by MeO-CyHQ-O-5HT (20 μM) with increasing pulse durations (1, 10, 50, 100, 200, 300, 400, 500, 600, 750, 1000 ms) after every 50 frames.

Video S18: Response of 5-HT (GRAB5-HT) sensor to a single 20-ms pulse of 405-nm light to activate MeO-CyHQ-O-5HT at a concentration of 20 μM respectively. See Video S18.

Video S19: Calcium influx measured in HEK 293 cells stably expressing TRPV1 channels after activation of MeO-CyHQ-VNA (5 μM) with a 250-ms flash of 740-nm light.

Video S20: Calcium influx measured in HEK 293 cells stably expressing TRPV1 channels after activation of MeO-CyHQ-EG (5 μM) with a 500-ms pulse of 740-nm light.

Video S21: Calcium influx measured in HEK 293 cells stably expressing TRPV1 channels after activation of MeO-CyHQ-VNA (2.5 μM) with a 50-ms flash of 405-nm light.

Video S22: Calcium influx measured in HEK 293 cells stably expressing TRPV1 channels after activation of MeO-CyHQ-EG (5 μM) with a 500-ms flash of 405-nm light.

UV-Vis Spectra

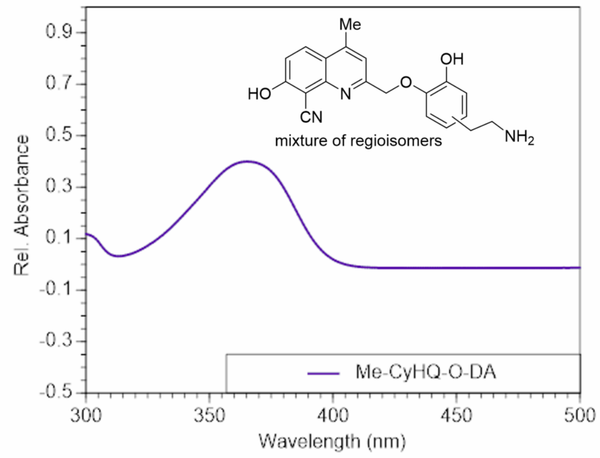

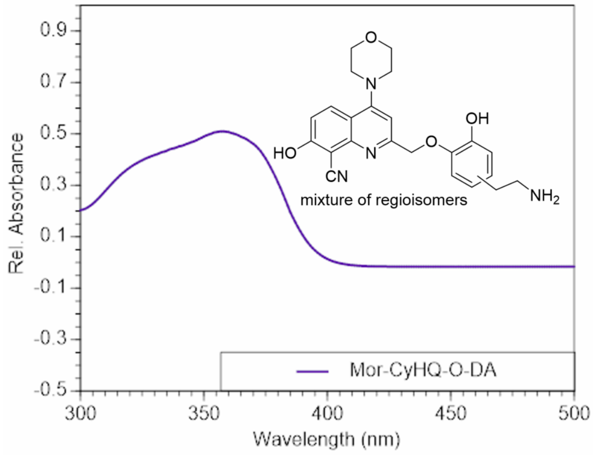

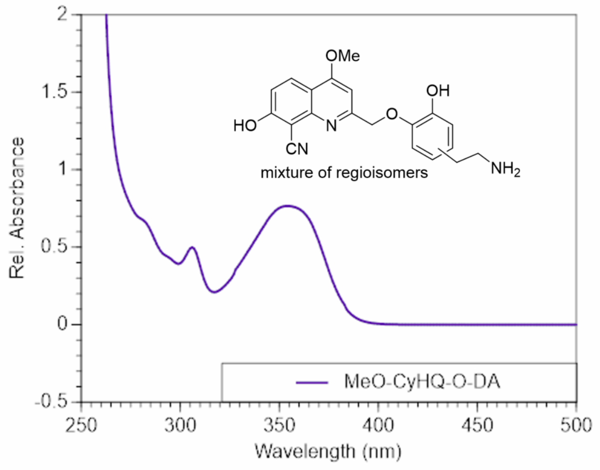

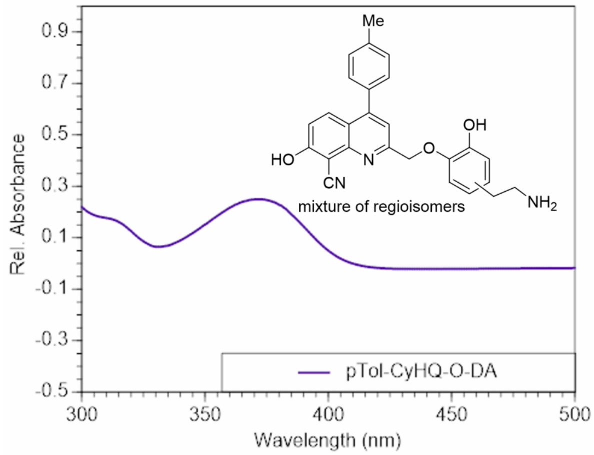

Me
OH
HO
N
O
CN
mixture of regioisomers
NH2
Rel. Absorbance
Wavelength (nm)
pTol-CyHQ-O-DA

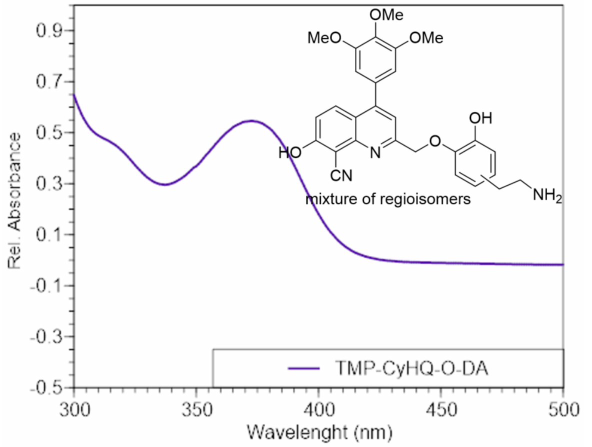

OMe
MeO
OMe
OH
HO
N
O
CN
mixture of regioisomers
NH2
Rel. Absorbance
Wavelenght (nm)
TMP-CyHQ-O-DA

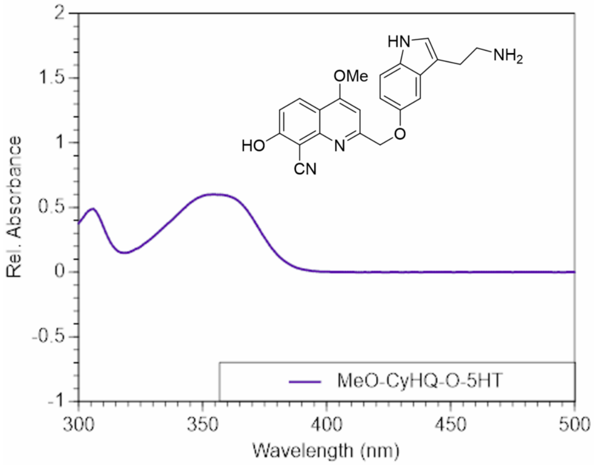

HN
NH2
OMe
HO
N
O
CN
Rel. Absorbance
Wavelength (nm)
MeO-CyHQ-O-5HT

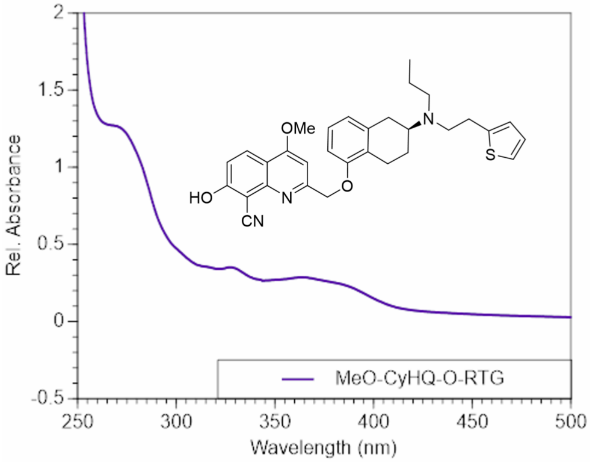
OMe
HO
N
CN
O
N
S
Rel. Absorbance
Wavelength (nm)
MeO-CyHQ-O-RTG

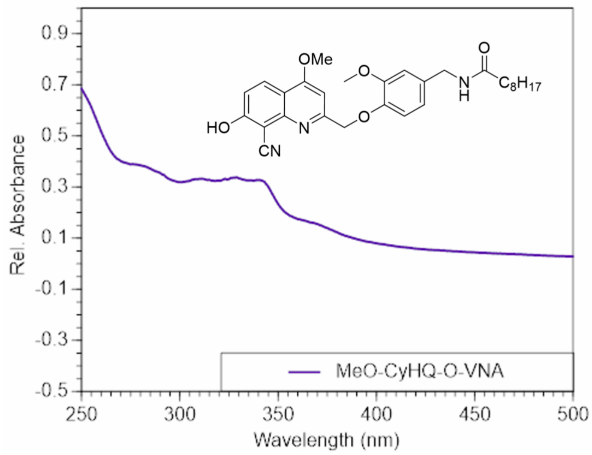
OMe
O
HO
N
CN
O
N
H
C8H17
Rel. Absorbance
Wavelength (nm)
MeO-CyHQ-O-VNA

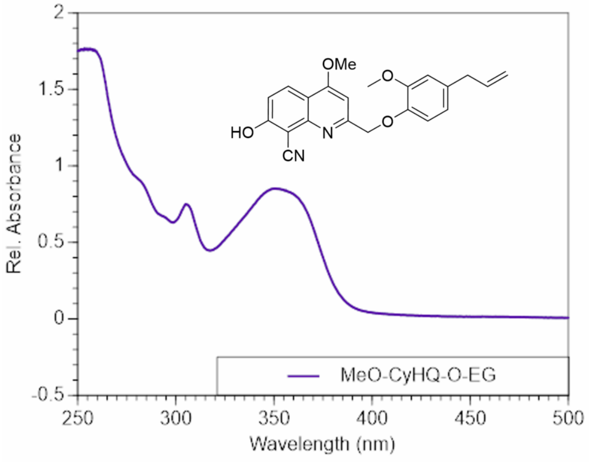
OMe
O
HO
N
CN
O
Rel. Absorbance
Wavelength (nm)
MeO-CyHQ-O-EG

NMR Spectra

(8-cyano-7-(methoxymethoxy)-4-methylquinolin-2-yl)methyl methanesulfonate (**2a**)

^{1}H NMR (500 MHz, chloroform-*d*)

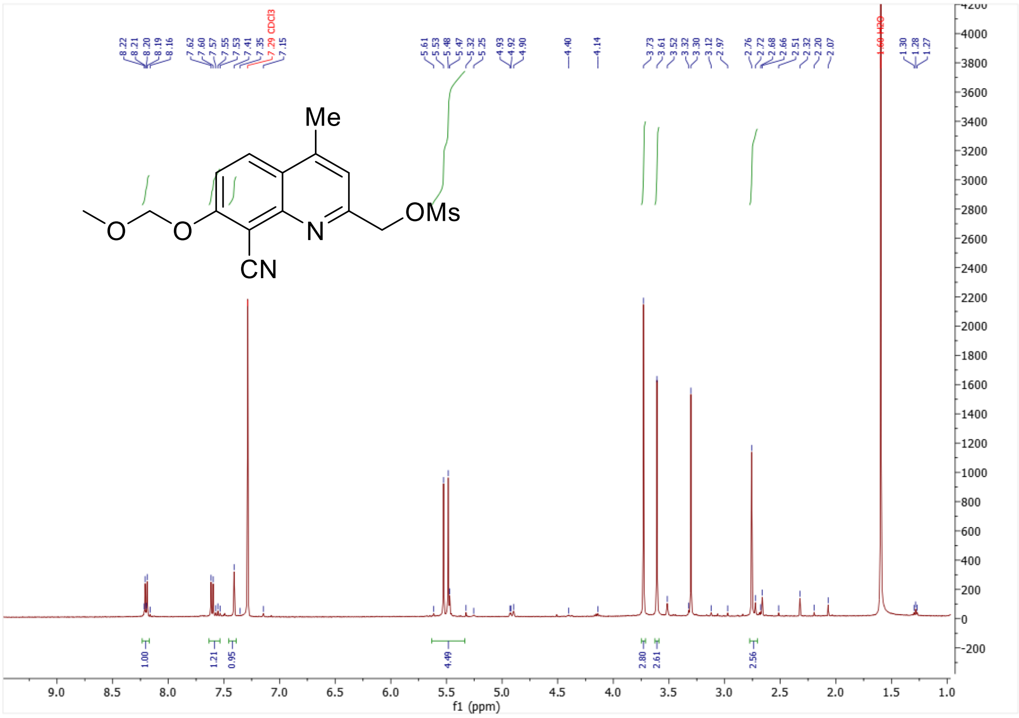

^{13}C NMR (126 MHz, chloroform-d)

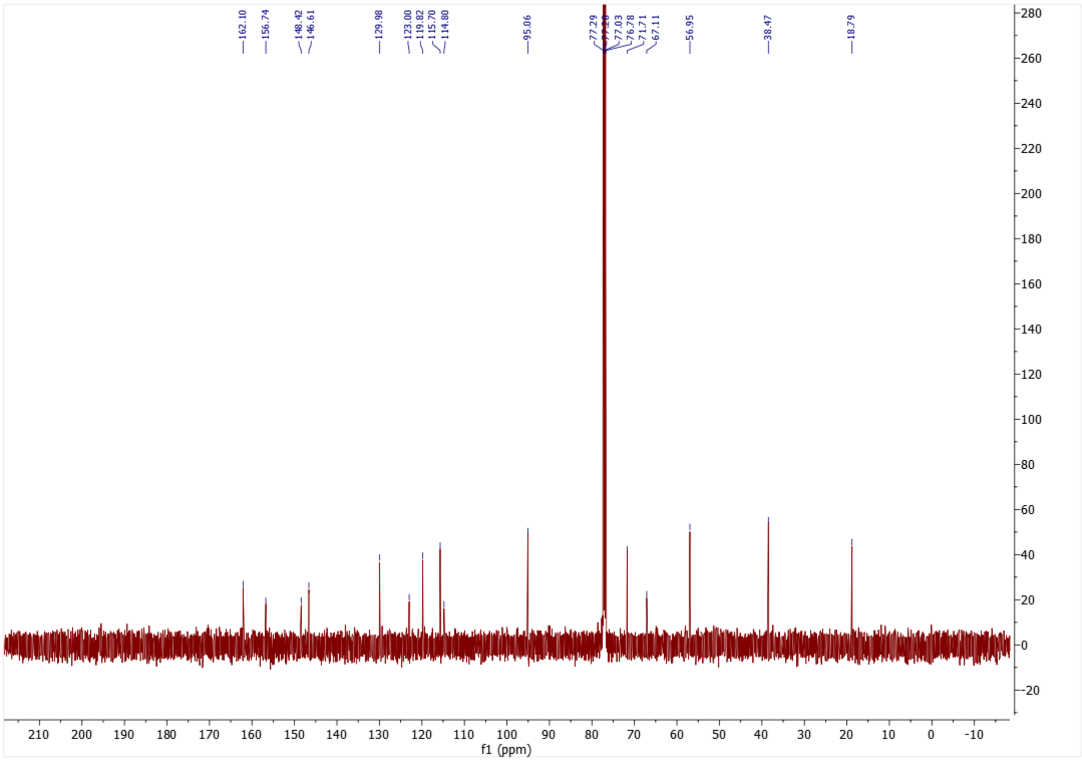

(8-cyano-7-(methoxymethoxy)-4-morpholinoquinolin-2-yl)methyl methanesulfonate (**2b**)

^{1}H NMR (500 MHz, chloroform-*d*)

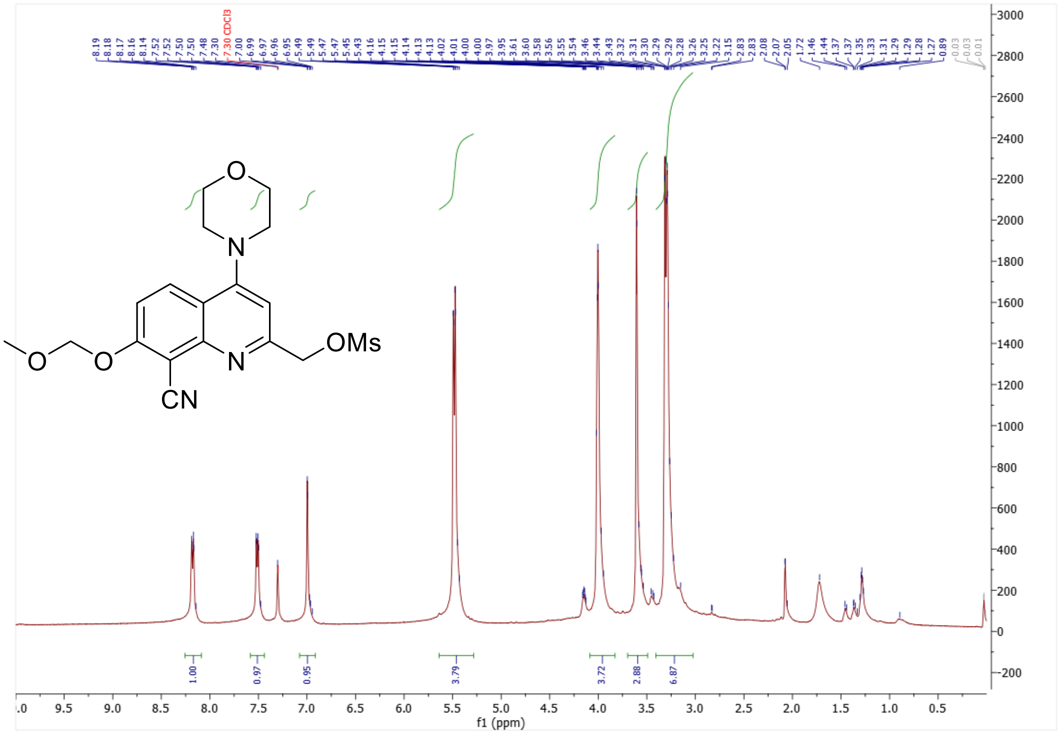

^{13}C NMR (126 MHz, chloroform-*d*)

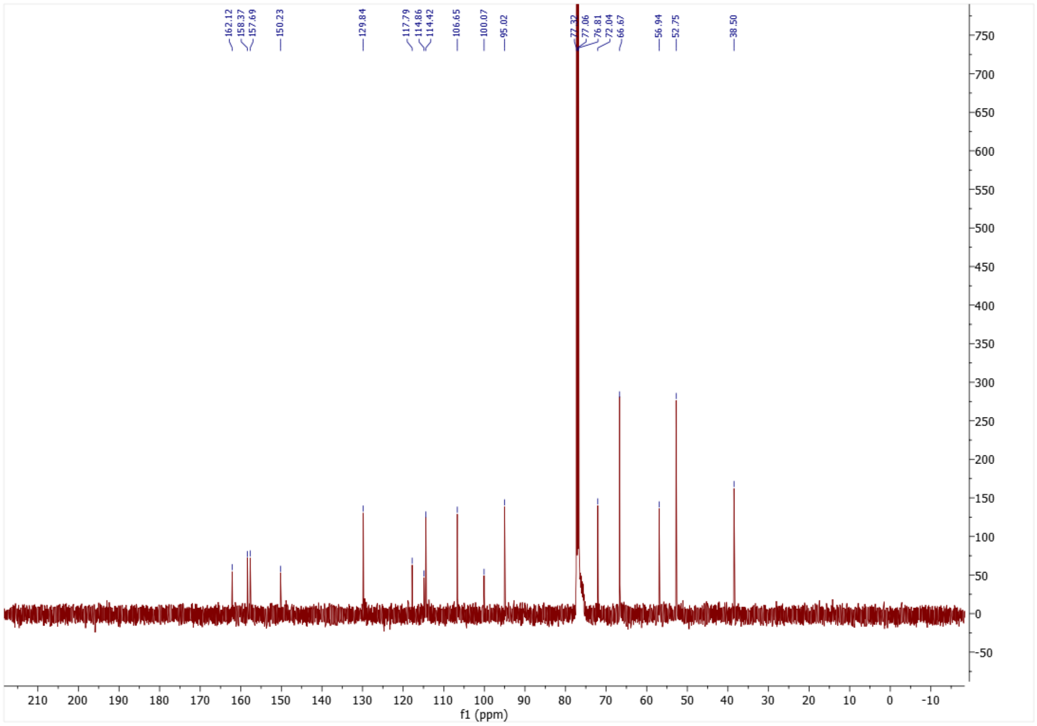

(8-Cyano-4-methoxy-7-(methoxymethoxy)quinolin-2-yl)methyl methanesulfonate (**2c**)

^{1}H NMR (500 MHz, chloroform-*d*)

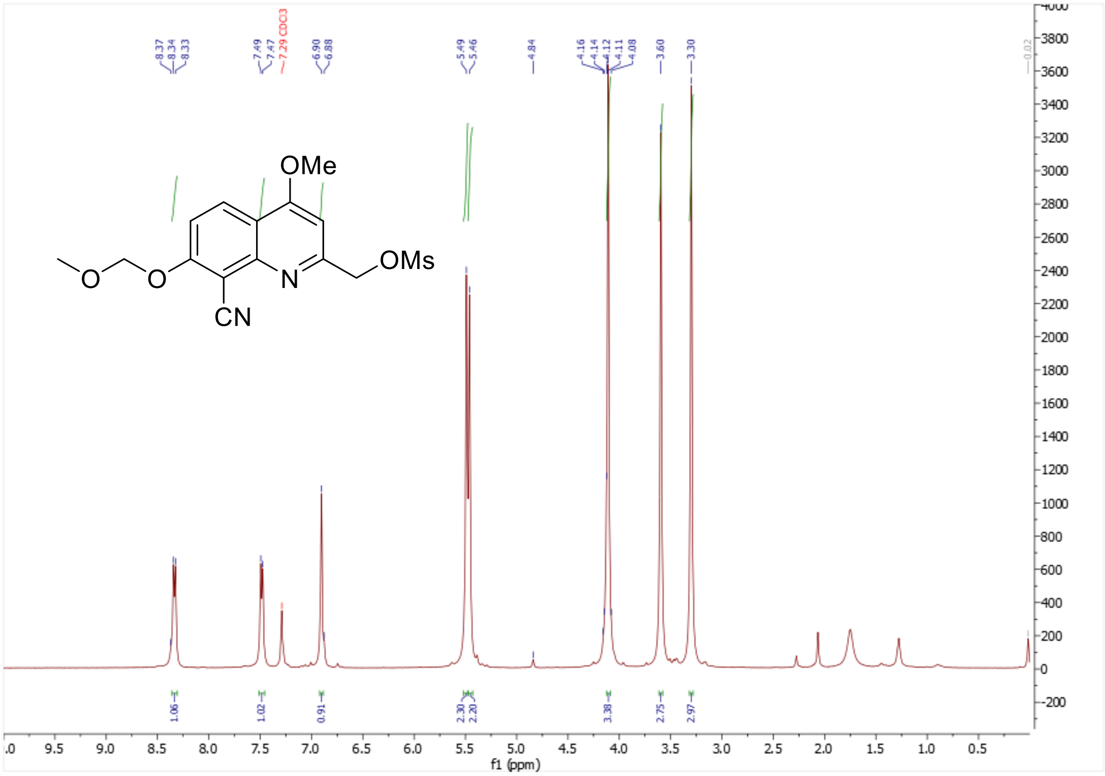

^{13}C NMR (126 MHz, chloroform-*d)*

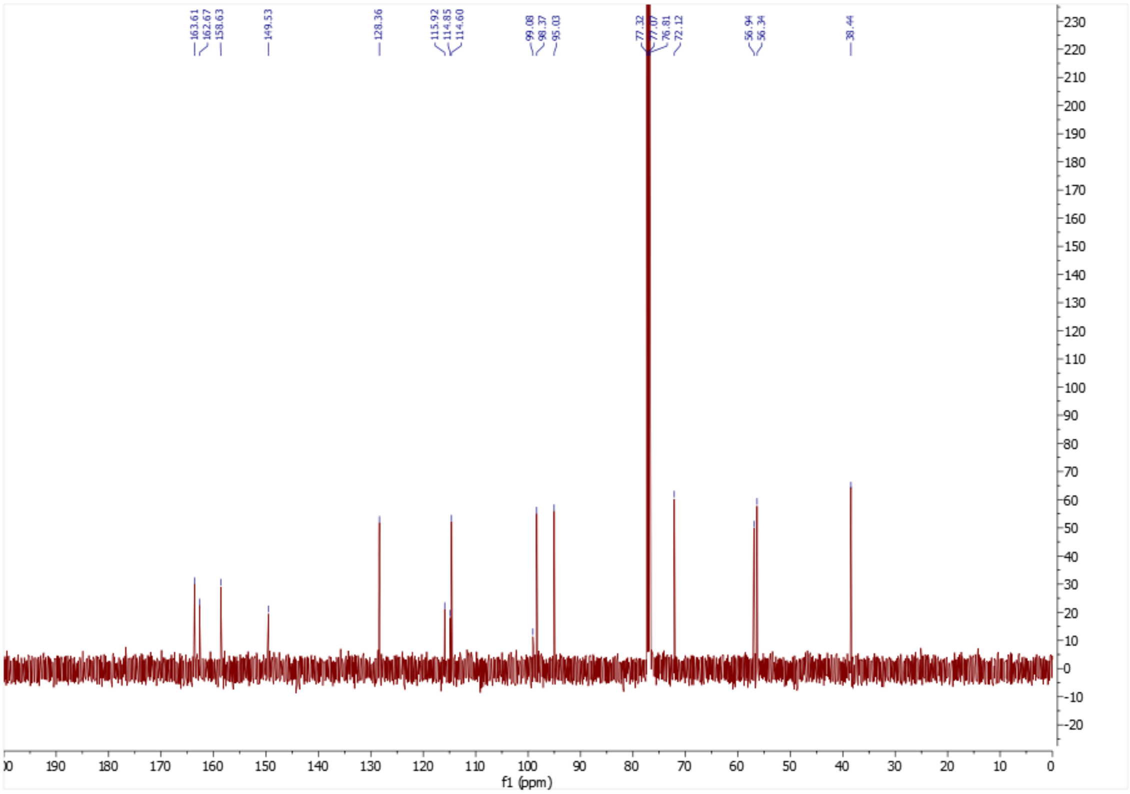

(8-cyano-7-(methoxymethoxy)-4-(p-tolyl)quinolin-2-yl)methyl methanesulfonate (**2d**)

^{1}H NMR (500 MHz, chloroform-*d*)

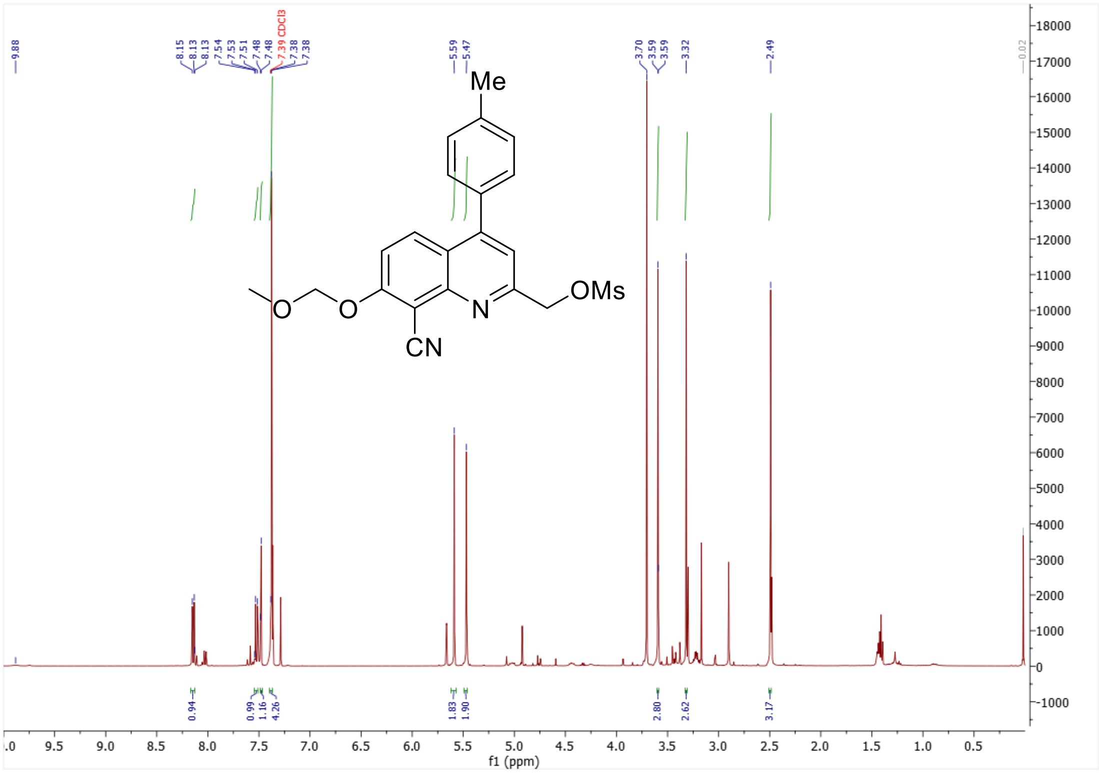

^{13}C NMR (126 MHz, chloroform-*d*)

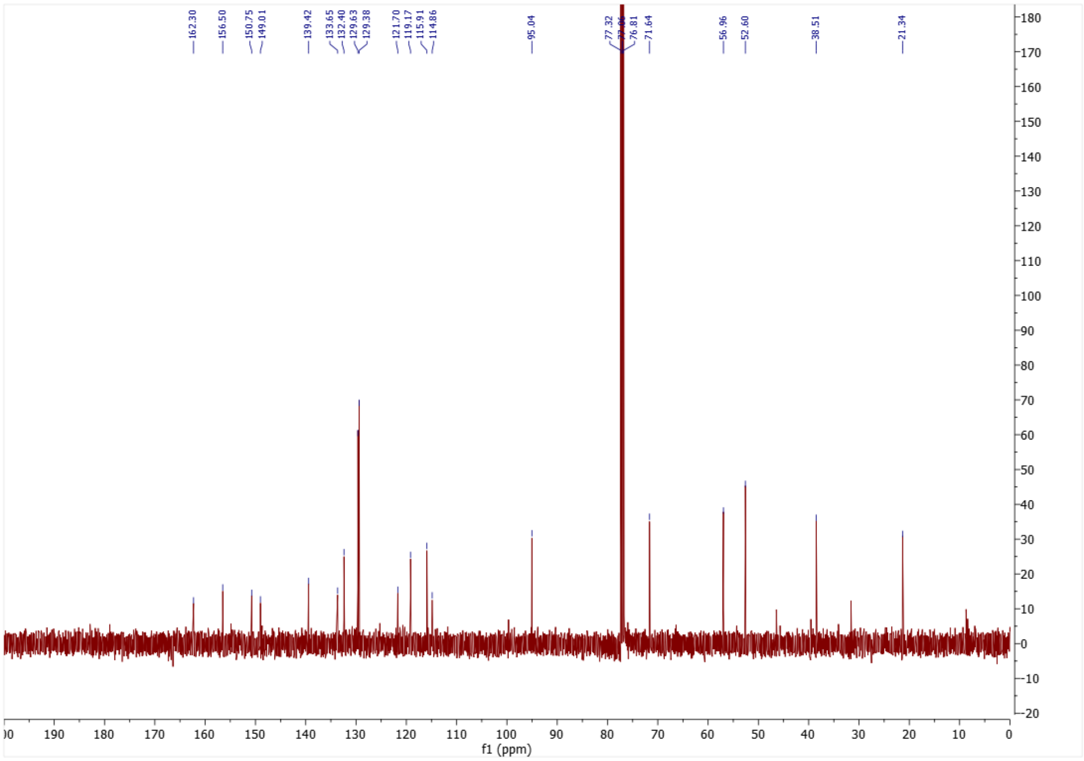

(8-cyano-7-(methoxymethoxy)-4-(3,4,5-trimethoxyphenyl)quinolin-2-yl)methyl methanesulfonate **(2e)**

^{1}H NMR (500 MHz, chloroform-*d*)

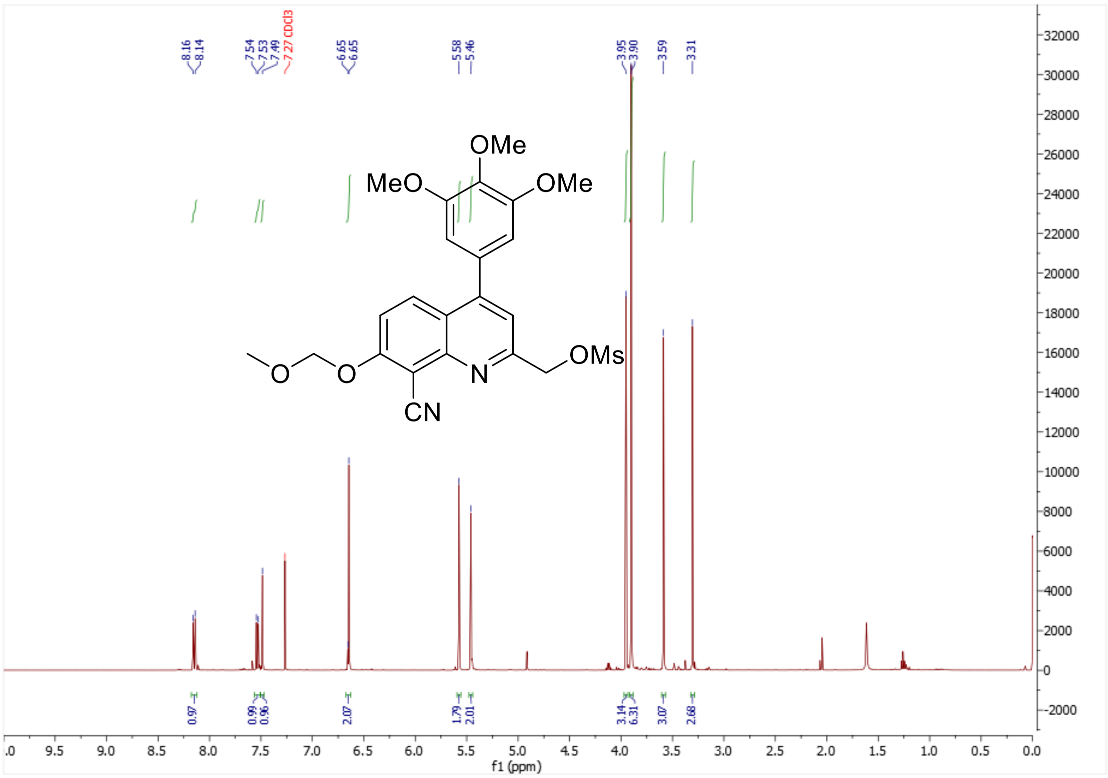

^{13}C NMR (126 MHz, chloroform-*d*)

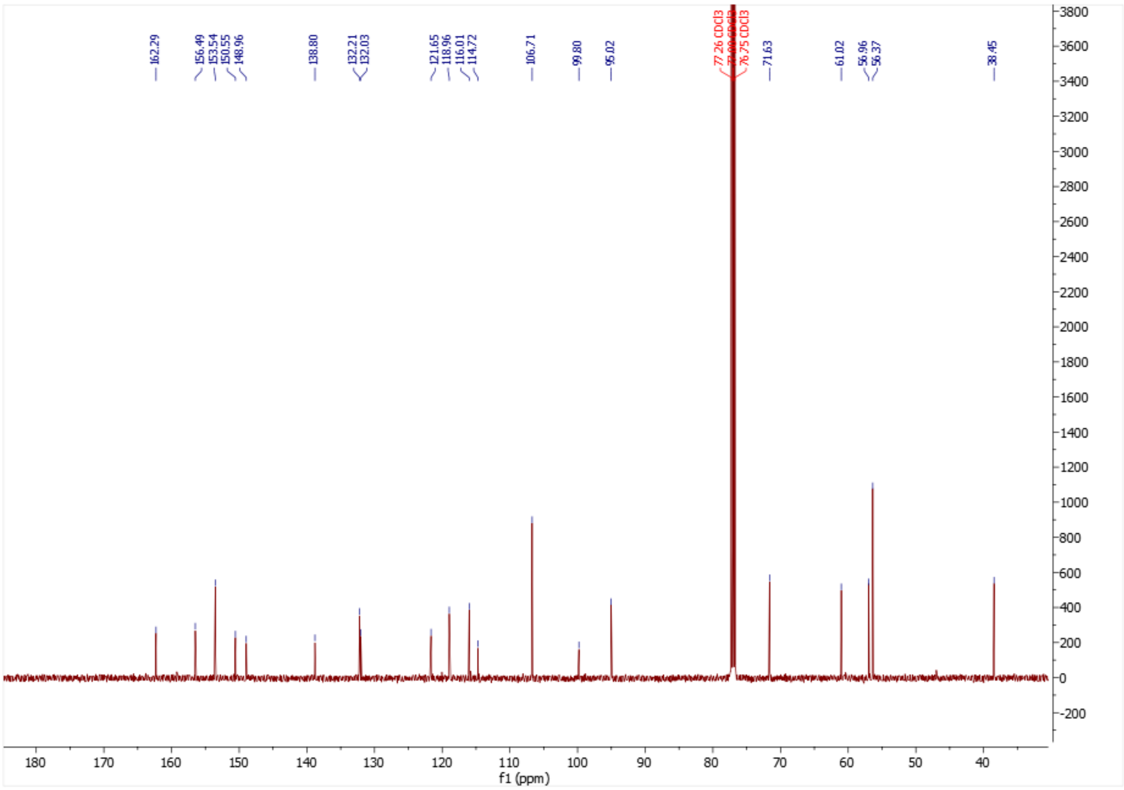

2-(3-((8-cyano-7-hydroxy-4-methylquinolin-2-yl)methoxy)-4-hydroxyphenyl)ethan-1-aminium 2,2,2-trifluoroacetate **(3a)**

^{1}H NMR (500 MHz, methanol-d_4)

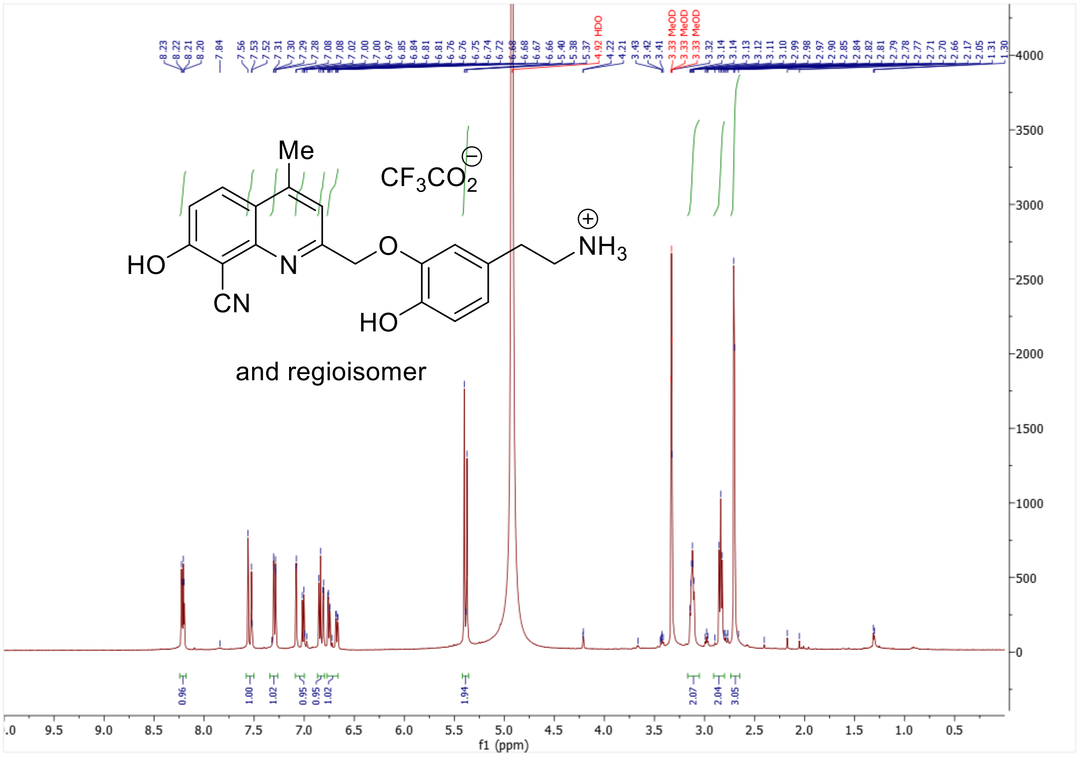

^{13}C NMR (126 MHz, methanol-d_4)

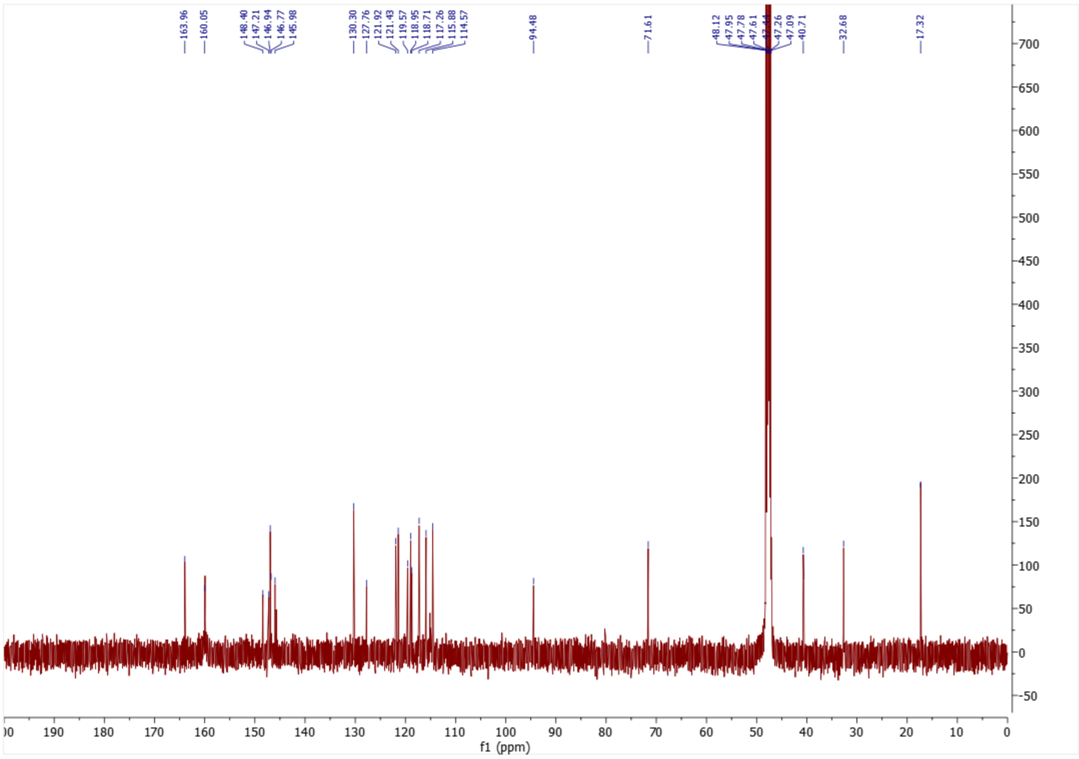

2-(3-((8-cyano-7-hydroxy-4-morpholinoquinolin-2-yl)methoxy)-4-hydroxyphenyl)ethan-1-aminium 2,2,2-trifluoroacetate **(3b)**

^{1}H NMR (500 MHz, acetonitrile-d_3)

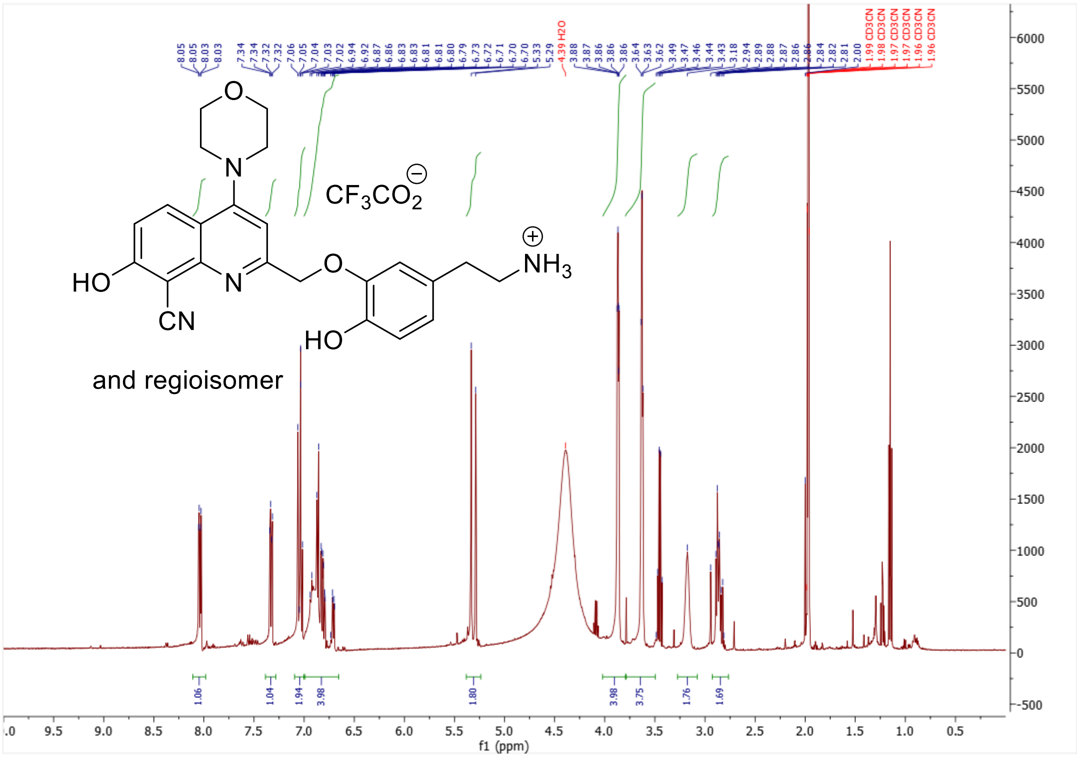

^{13}C NMR (126 MHz, acetonitrile-d_3)

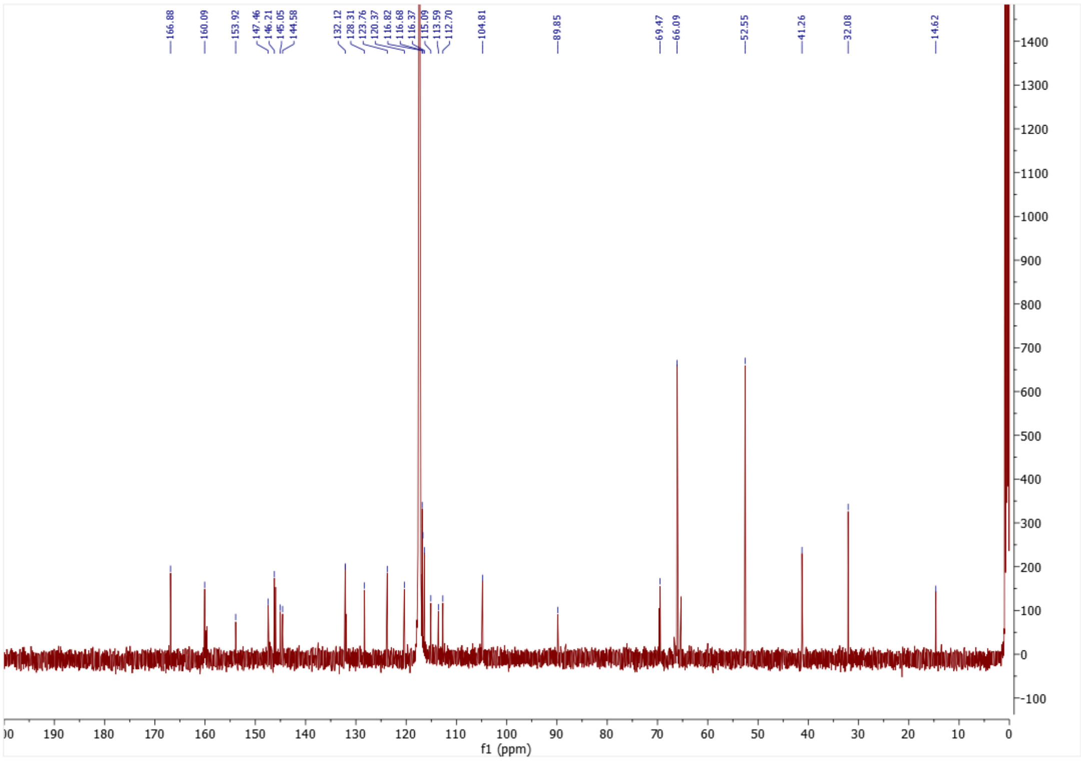

2-(3-((8-cyano-7-hydroxy-4-methylquinolin-2-yl)methoxy)-4-hydroxyphenyl)ethan-1-aminium 2,2,2-trifluoroacetate **(3c)**

^{1}H NMR (500 MHz, acetonitrile-d_3)

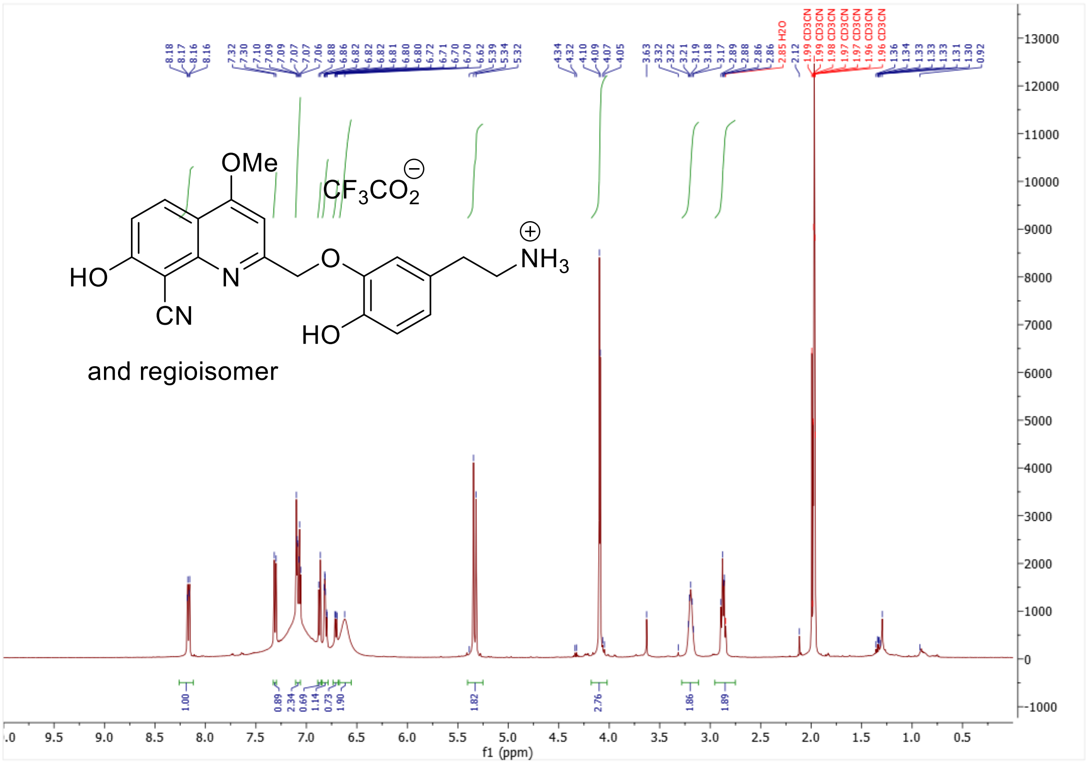

^{13}C NMR (126 MHz, acetonitrile-d_3)

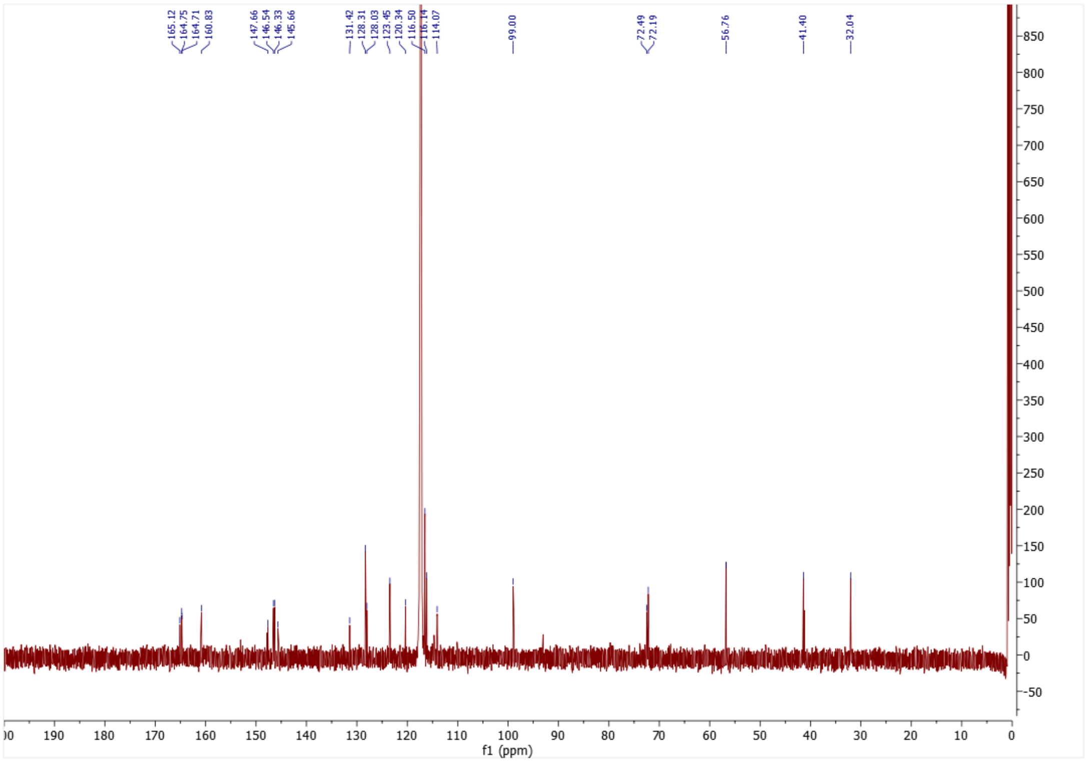

2-(3-((8-cyano-7-hydroxy-4-(p-tolyl)quinolin-2-yl)methoxy)-4-hydroxyphenyl)ethan-1-aminium 2,2,2-trifluoroacetate **(3d)**

^{1}H NMR (500 MHz, acetonitrile-d_3)

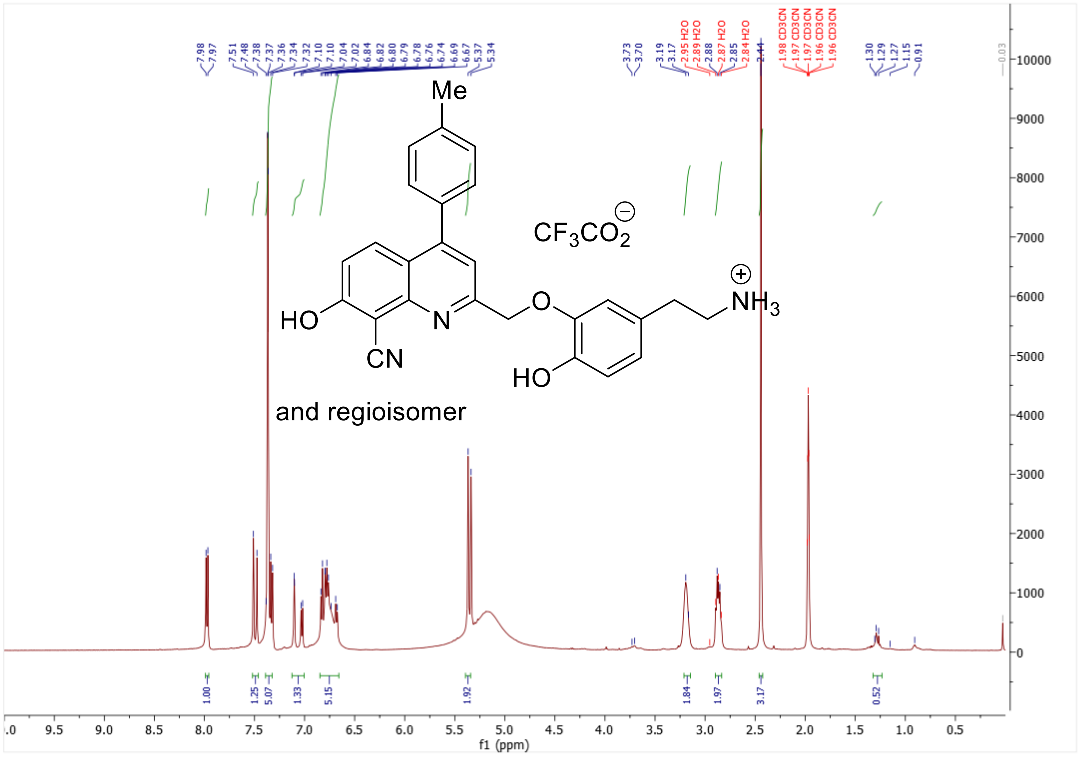

^{13}C NMR (126 MHz, acetonitrile-d_3)

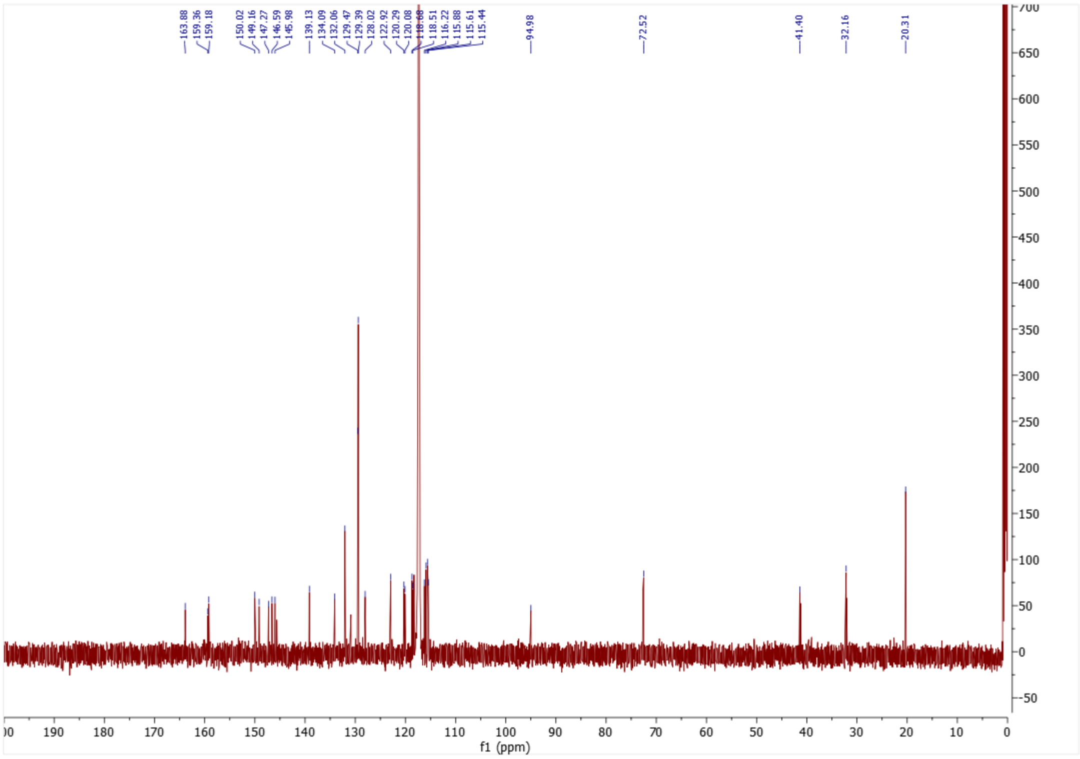

2-(3-((8-cyano-7-hydroxy-4-(2,4,6-trimethoxyphenyl)quinolin-2-yl)methoxy)-4-hydroxyphenyl)ethan-1-aminium 2,2,2-trifluoroacetate **(3e)**

^{1}H NMR (500 MHz, methanol-d_4)

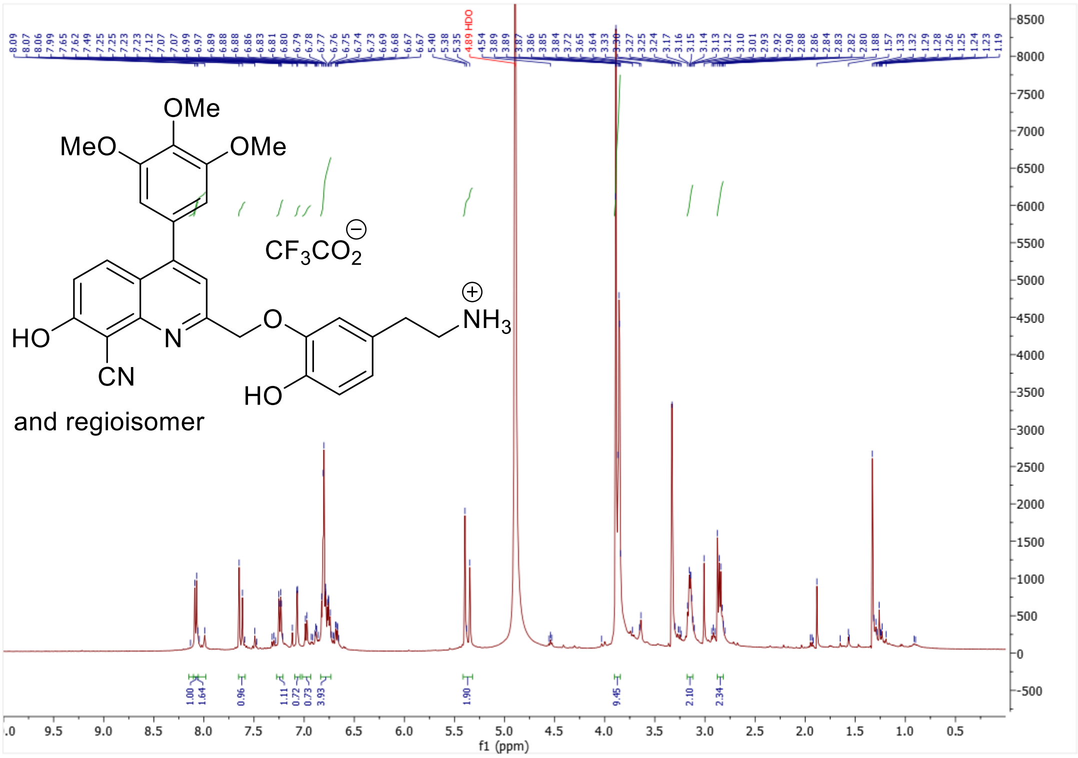

^{13}C NMR (126 MHz, methanol-d_4)

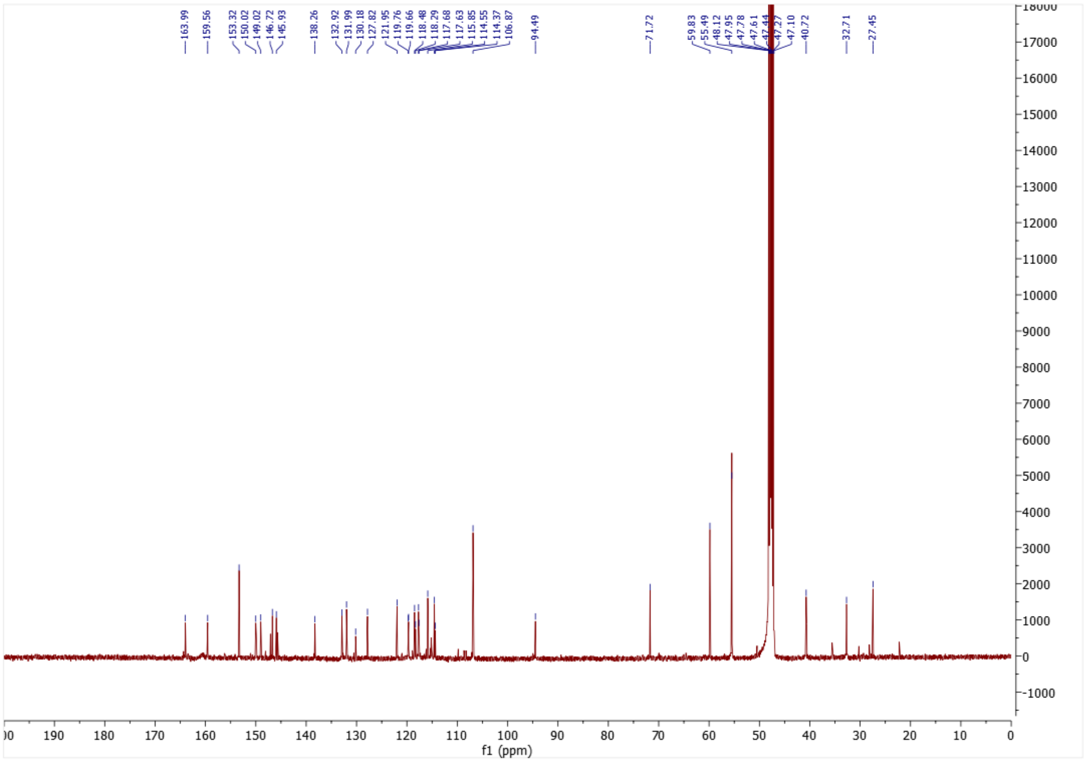

2-(5-((8-cyano-7-hydroxy-4-methoxyquinolin-2-yl)methoxy)-1H-indol-3-yl)ethan-1-aminium 2,2,2-trifluoroacetate **(4c)**

^{1}H NMR (500 MHz, methanol-d_4)

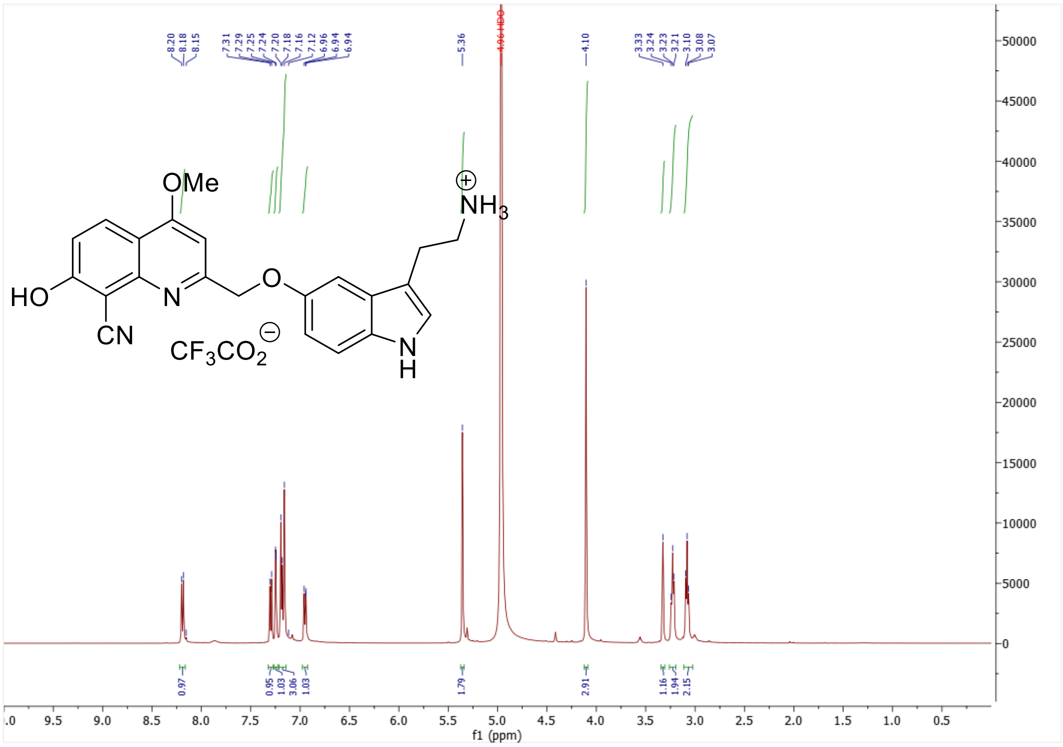

^{13}C NMR (126 MHz, methanol- d_4)

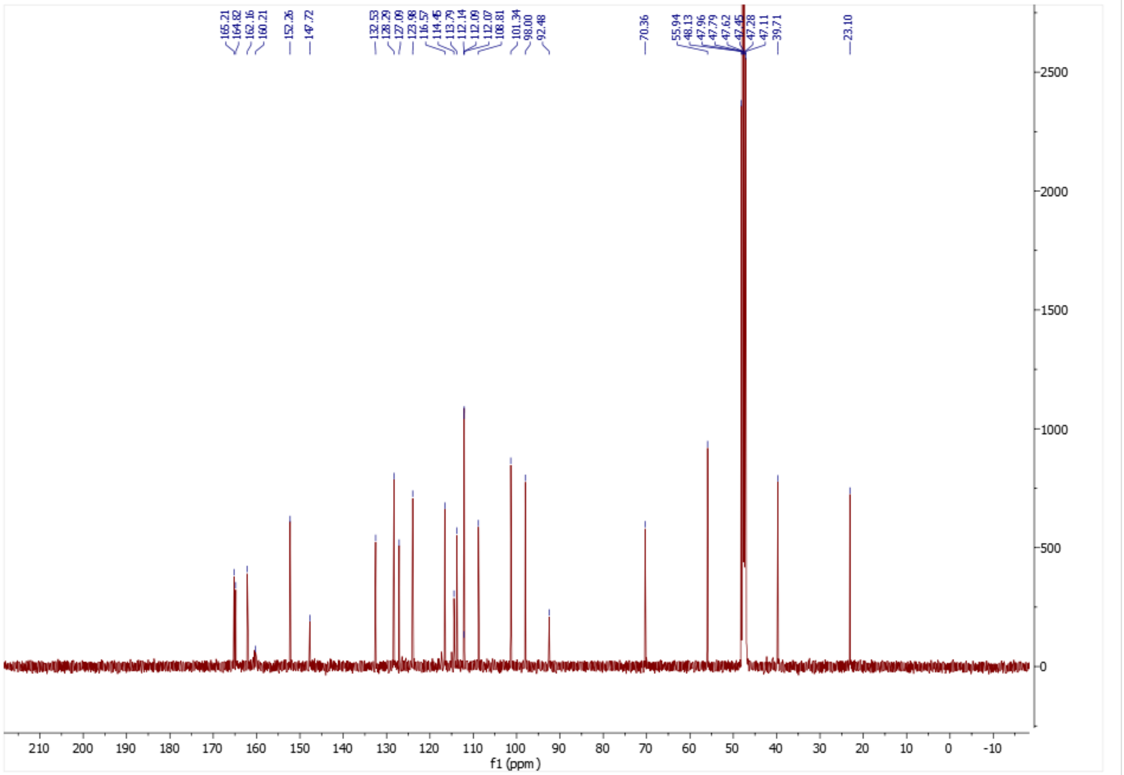

(R)-7-hydroxy-4-methoxy-2-(((6-(propyl(2-(thiophen-2-yl)ethyl)amino)-5,6,7,8-tetrahydronaphthalen-1-yl)oxy)methyl)quinoline-8-carbonitrile **(5c)**

^{1}H NMR (500 MHz, chloroform-*d*)

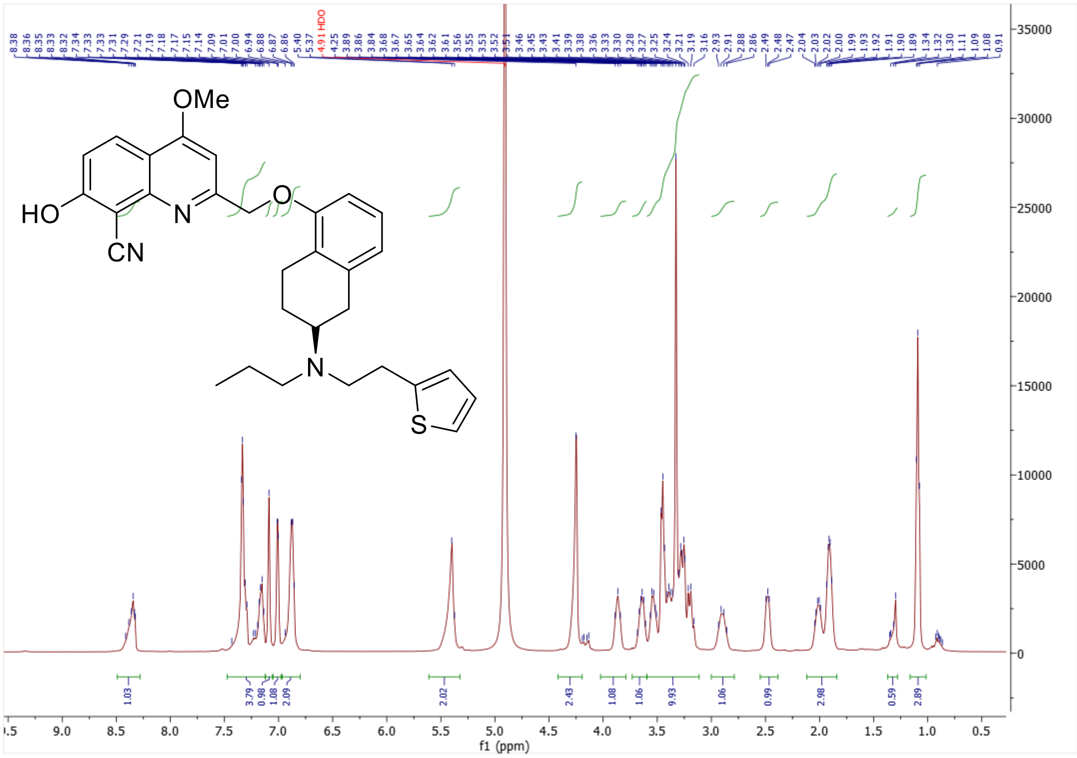

^{13}C NMR (126 MHz, chloroform-*d*)

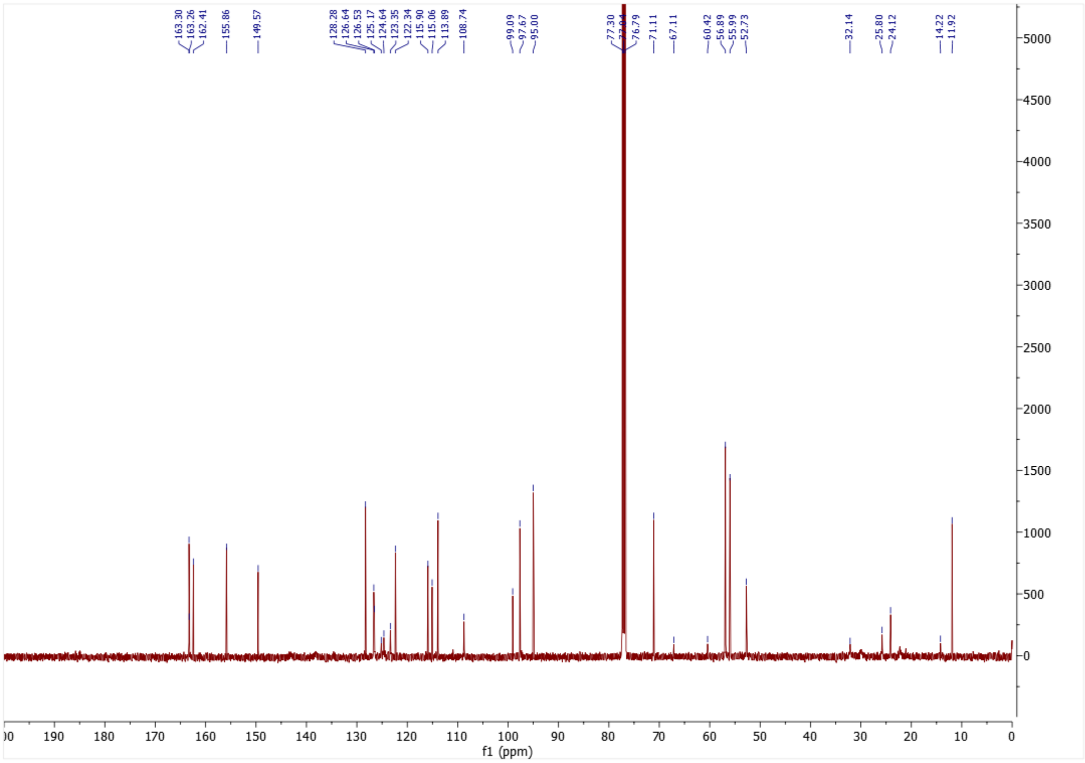

N-(4-((8-cyano-7-hydroxy-4-methoxyquinolin-2-yl)methoxy)-3-methoxybenzyl)nonanamide **(6c)**

^{1}H NMR (500 MHz, acetone-d_6)

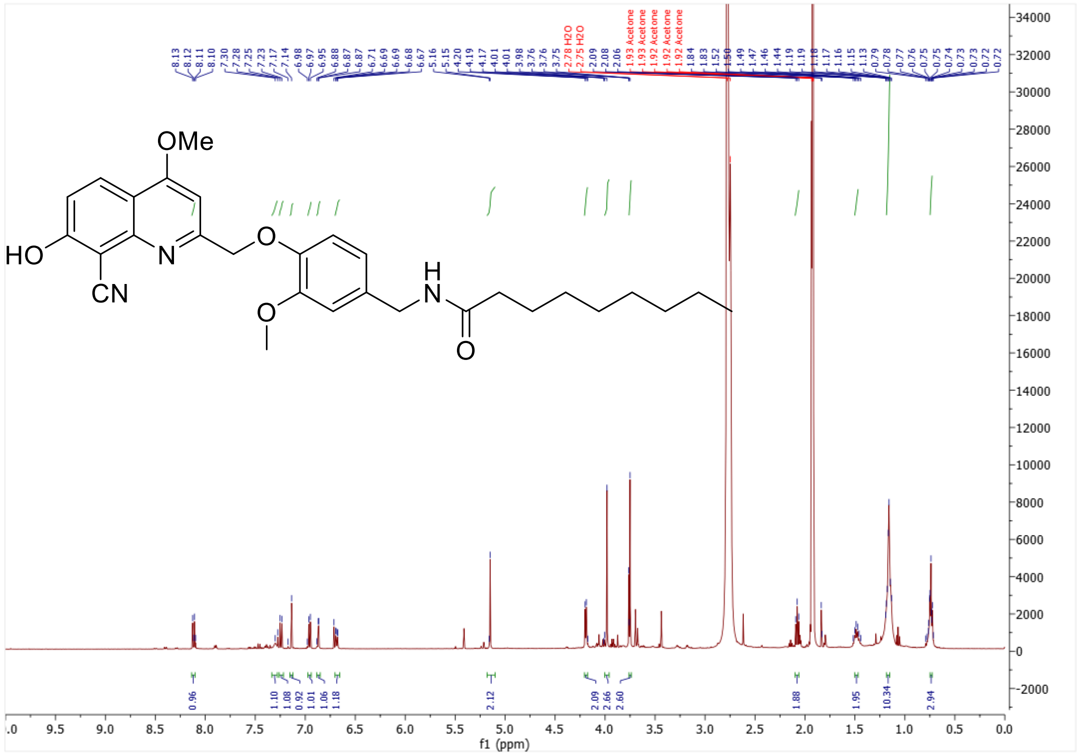

^{13}C NMR (126 MHz, methanol- d_4)

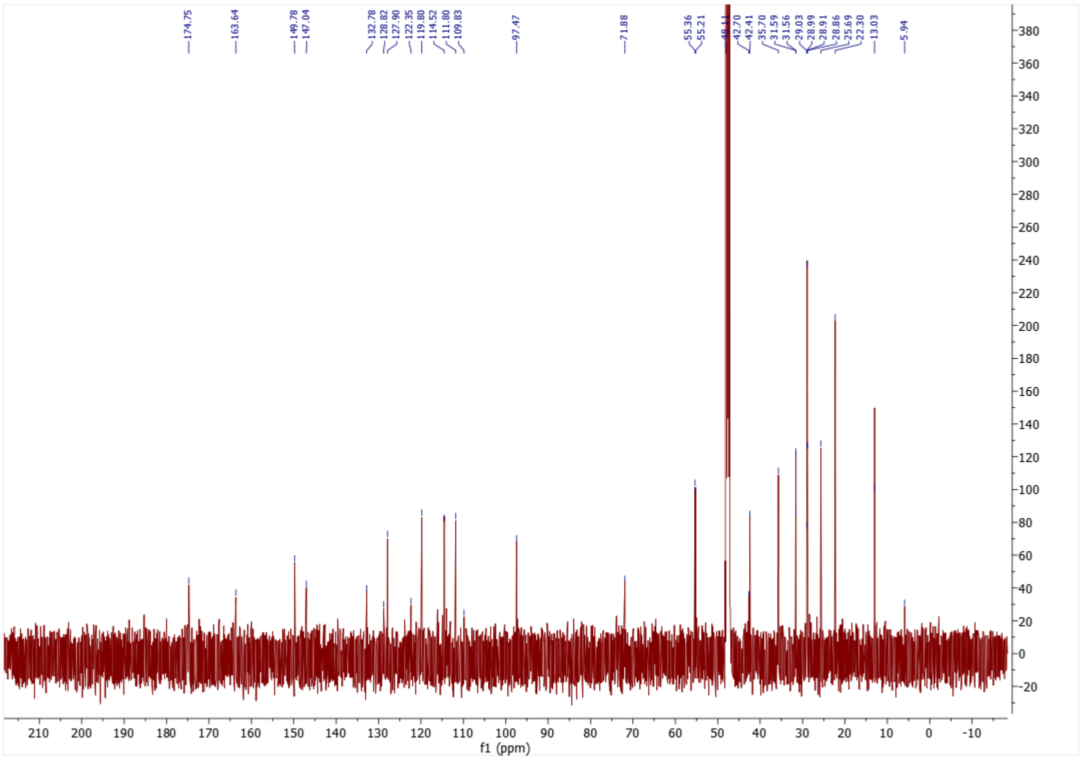

2-((4-allyl-2-methoxyphenoxy)methyl)-7-hydroxy-4-methoxyquinoline-8-carbonitrile **(7c)**

^{1}H NMR (500 MHz, methanol-d_4)

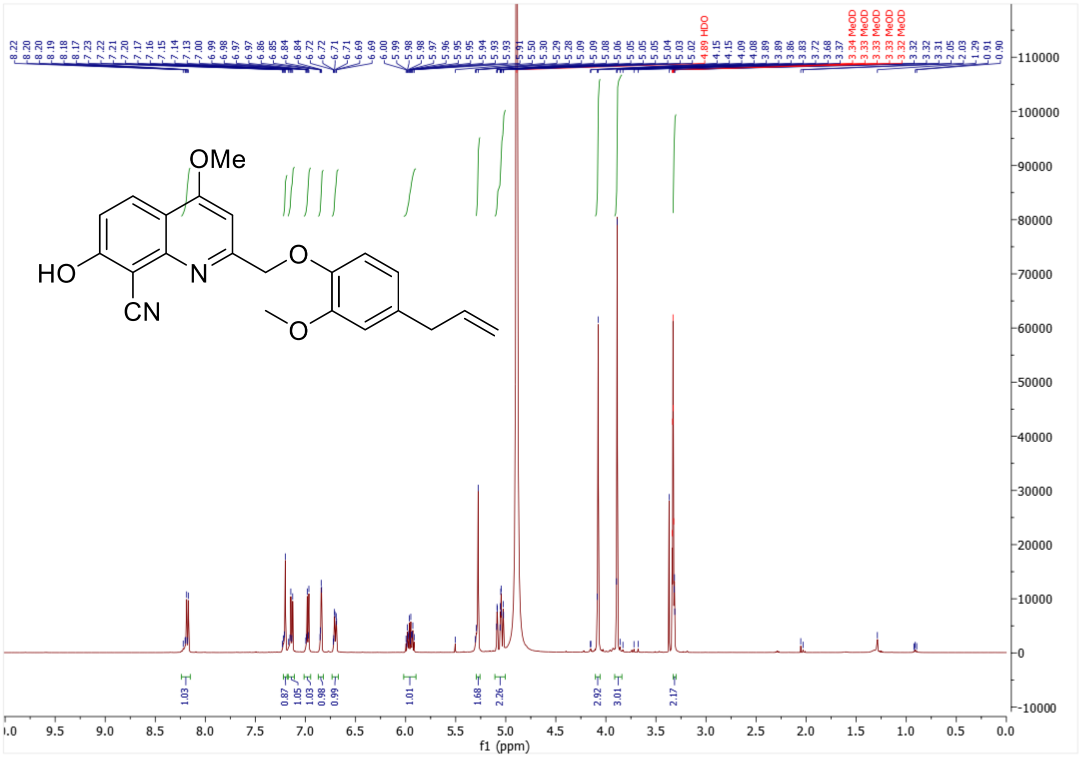

^{13}C NMR (126 MH z, methanol- d_4)

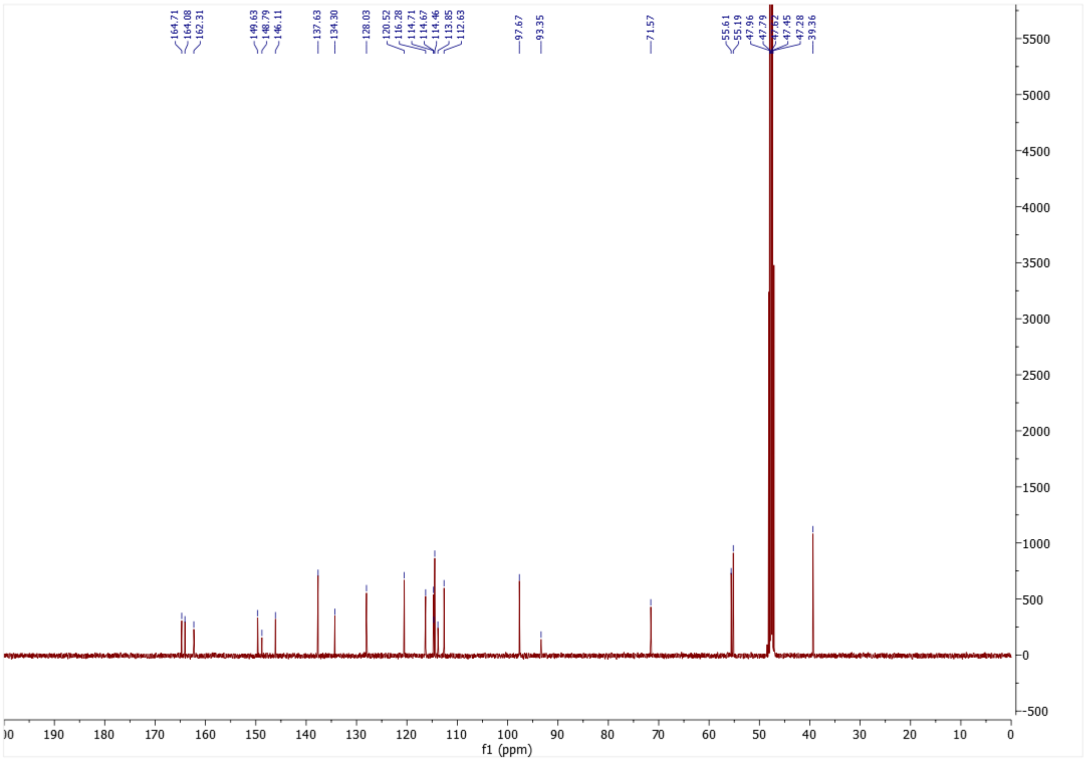

HPLC Data

Instrument: Agilent 1290 Infinity series uHPLC
Column: Zorbax Eclipse Plus C18 column
Mobile phase: A = 0.1% trifluoroacetic acid in water and B = acetonitrile
Gradient: 5% B to 100% B over 10 min, then re-equilibration to 5% B before the next run
Flow rate: 0.3 mL/min.
Observation wavelengths: 254, 280, and 320 nm.
Wavelengths used to detect different species in the photochemical reaction:

- Dopamine (DA): 280 and 254 nm
- Serotonin (5HT): 280 nm
- N-vanillyl-nonanoylamide (VNA): 280 nm
- Eugenol (EG): 280 nm
- Rotigotine (RT): 254 nm
- R-CyHQ-OH: 254 and 280 nm
- R-CyHQ-protected phenols: 254 and 280 nm

Me-CyHQ-DA (**3a**)
Reaction monitored at 254 nm for Me-CyHQ-DA (t_R = 8.3 and 8.4 min), DA (t_R = 0.74 min), and Me-CyHQ-OH (t_R = 3.5 min).

Photolysis at 365 nm (1PE)
Reaction time = 0 s

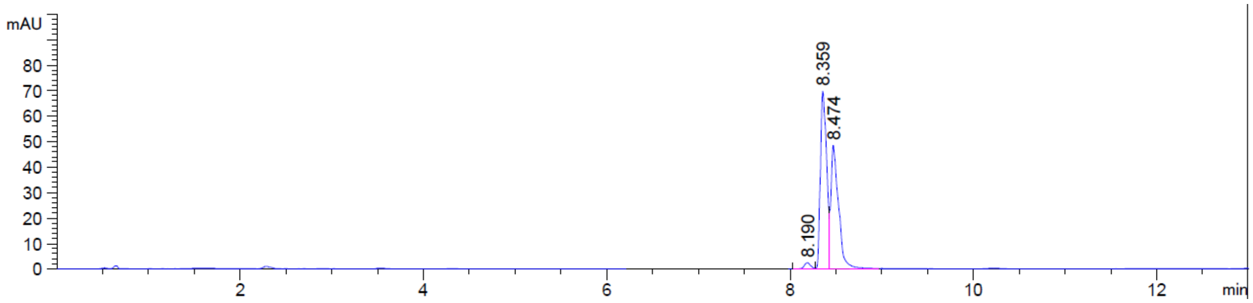

Reaction time = 05 s

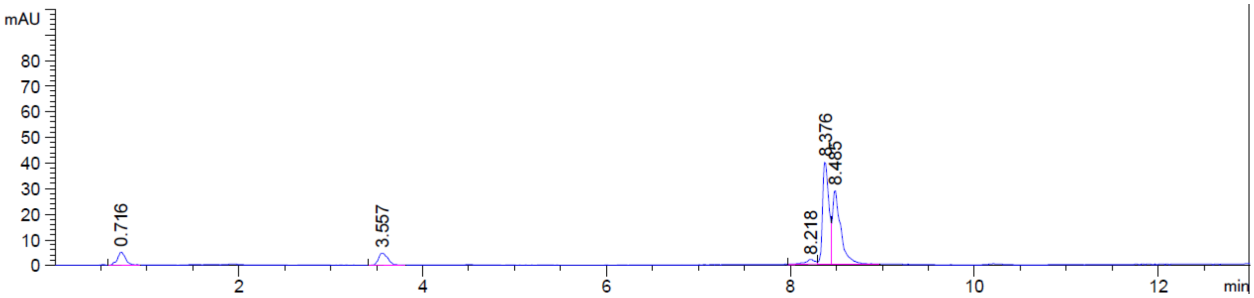

Reaction time = 20 s

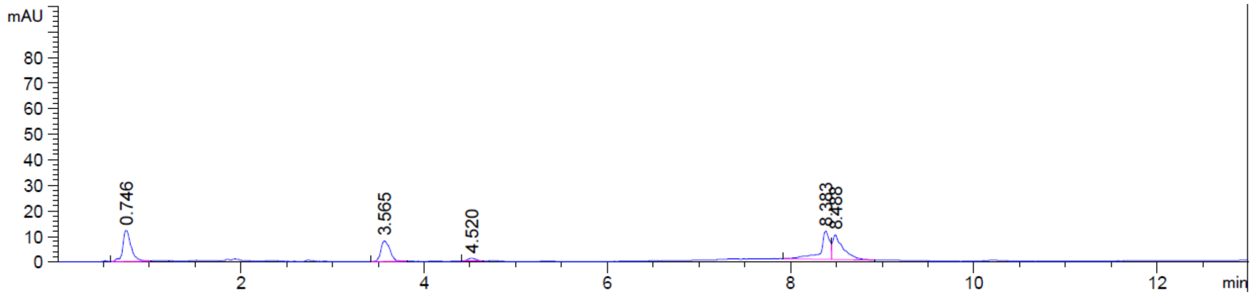

Reaction time = 40 s

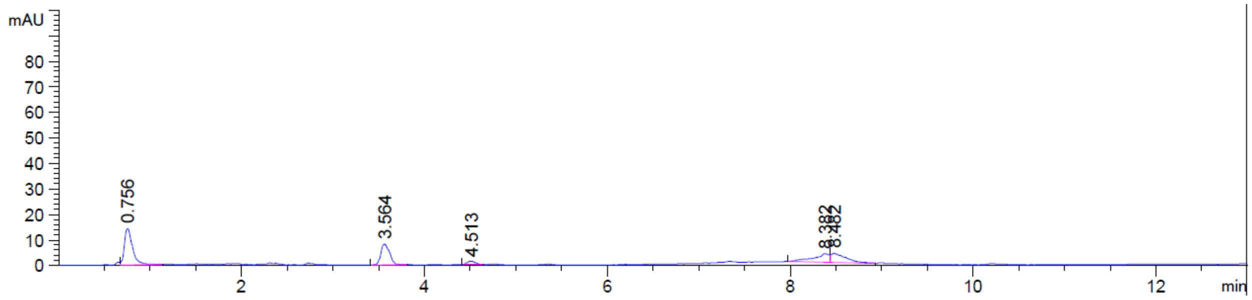

Photolysis at 740 nm (2PE)

Reaction time = 0 min

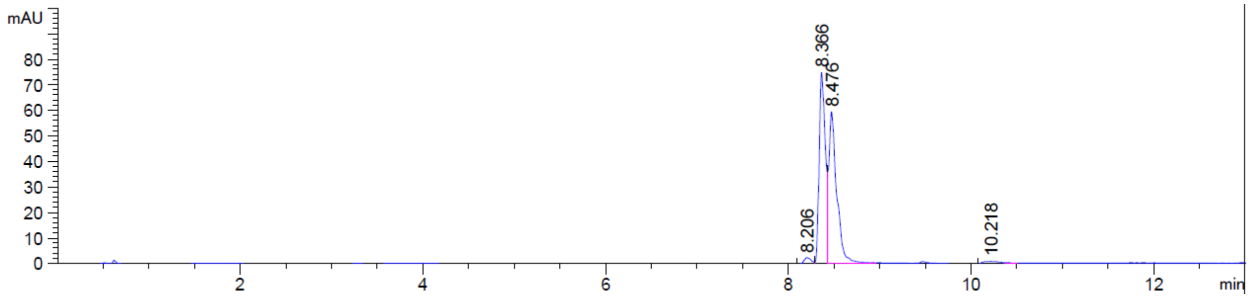

Reaction time = 10 min

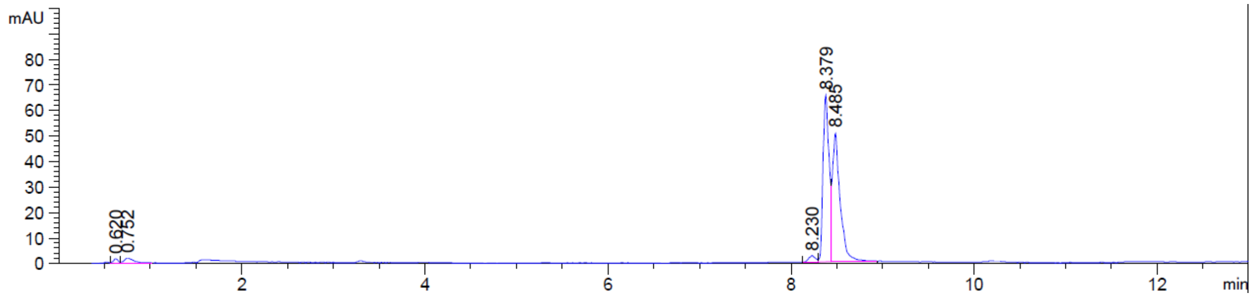

Reaction time = 30 min

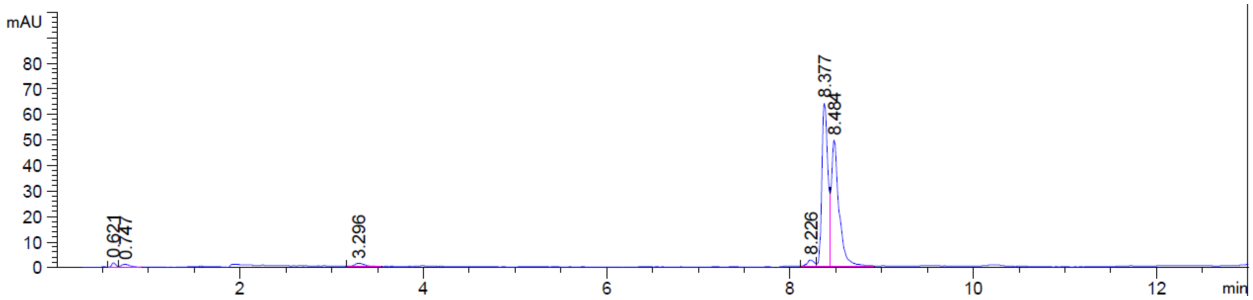

Stability toward spontaneous hydrolysis in the dark

Day 1

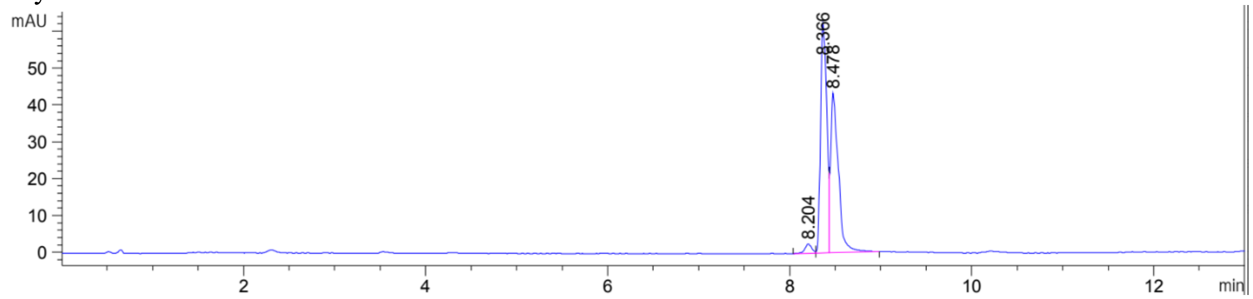

Day 2

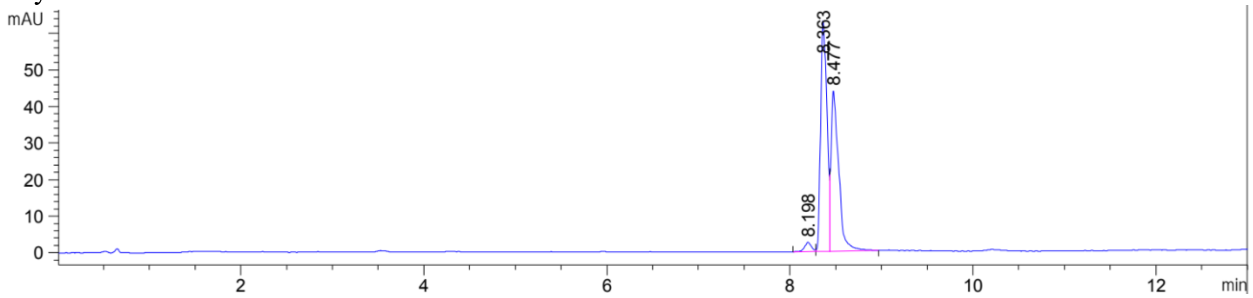

Day 3

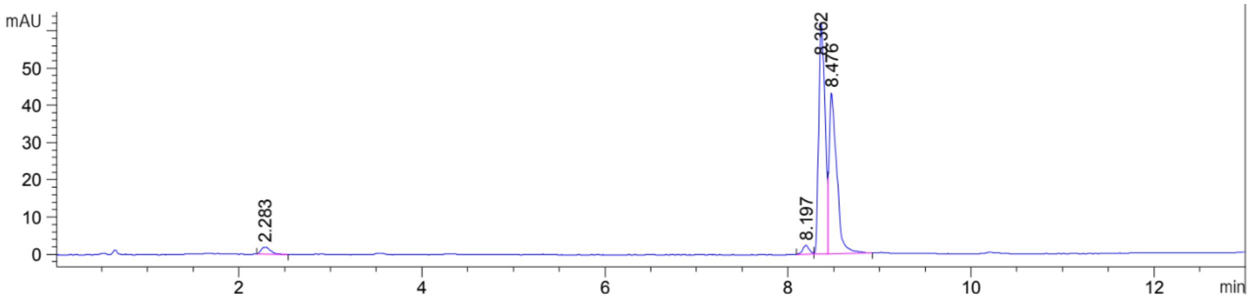

Mor-CyHQ-DA (**3b**)
Reaction monitored at 280 nm for Mor-CyHQ-DA (t_R = 2.5 min), DA (t_R = 0.83 min), and Mor-CyHQ-OH (t_R = 2.0 min).

Photolysis at 365 nm (1PE)
Reaction time = 0 s

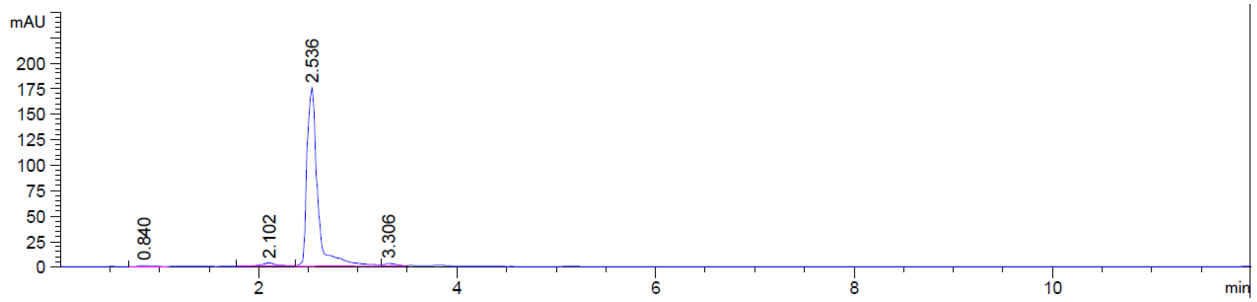

Reaction time = 05 s

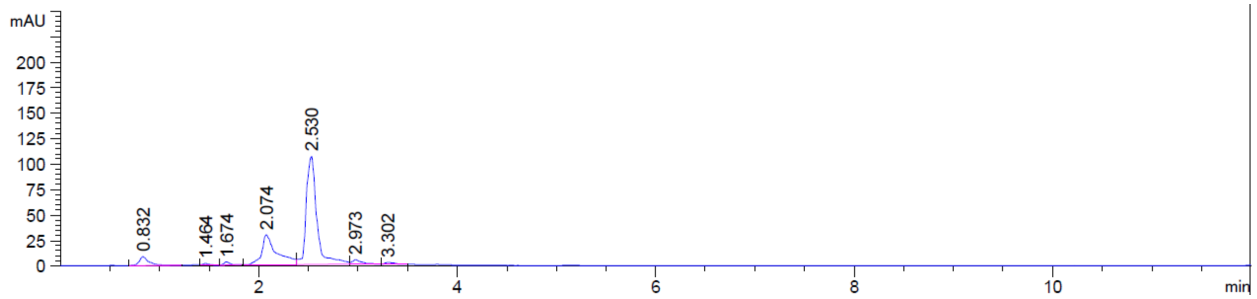

Reaction time = 20 s

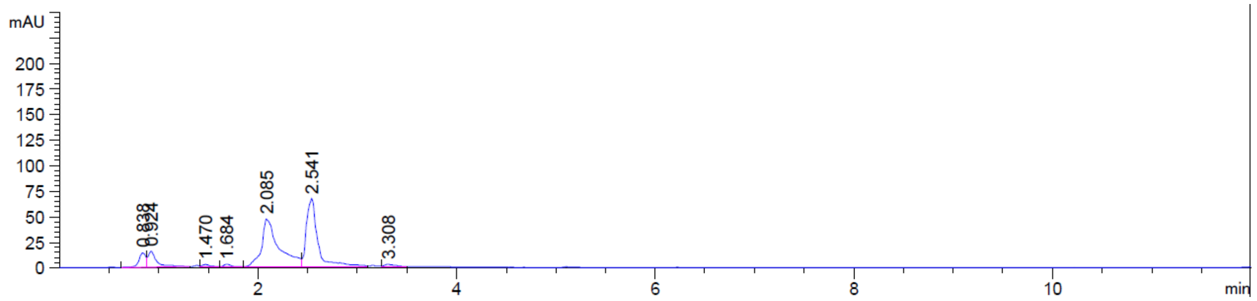

Reaction time = 40 s

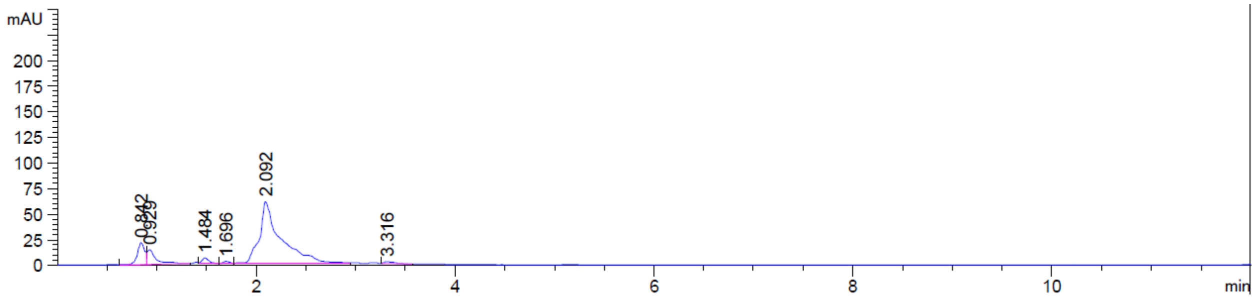

Photolysis at 740 nm (2PE)

Reaction time = 0 min

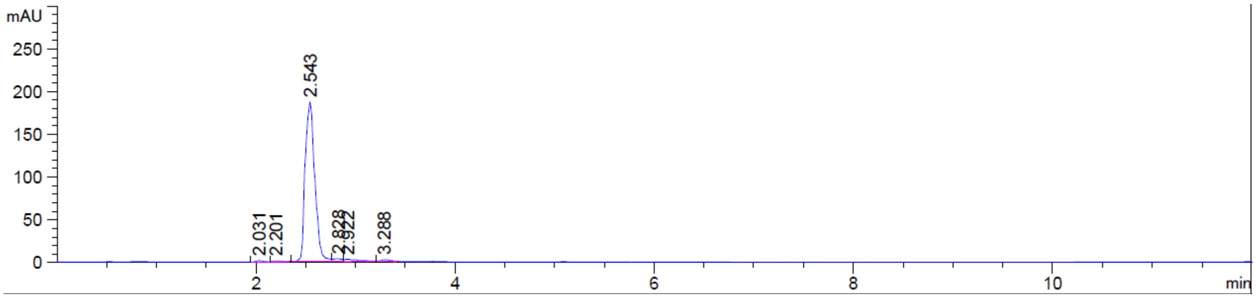

Reaction time = 10 min

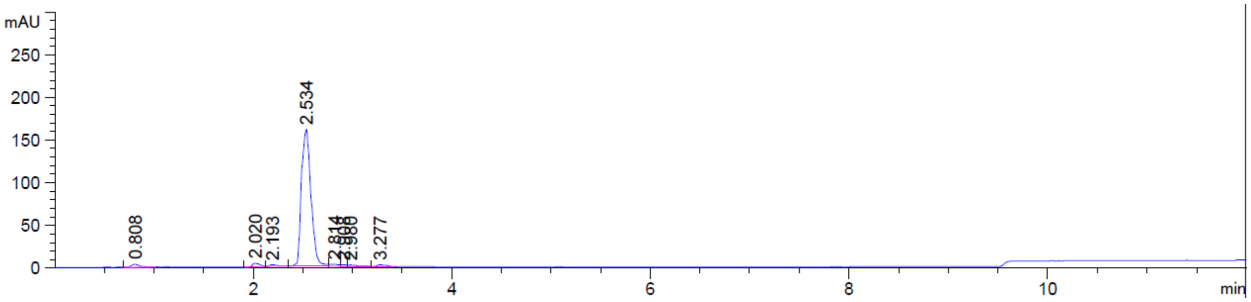

Reaction time = 30 min

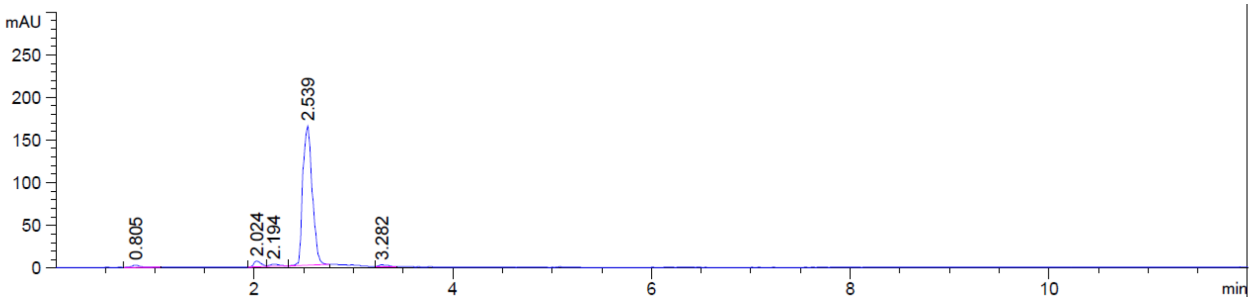

Stability toward spontaneous hydrolysis in the dark

Day 1

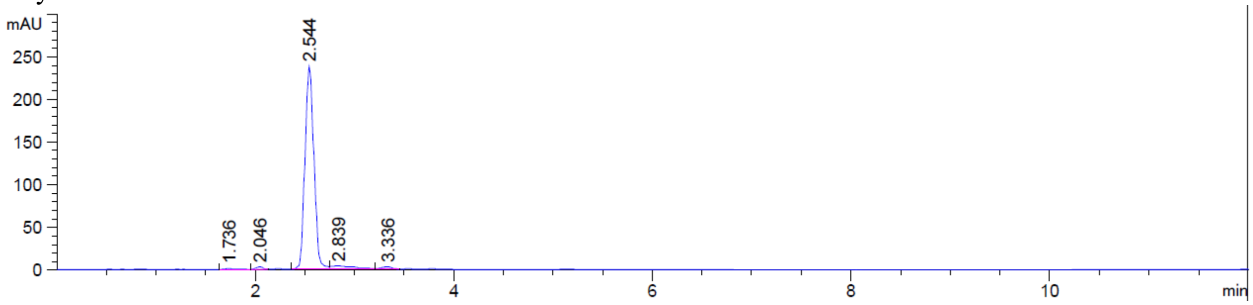

Day 2

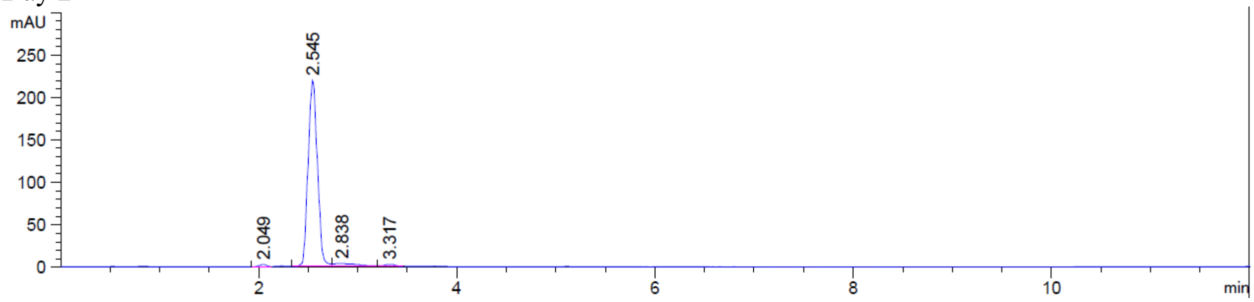

Day 3

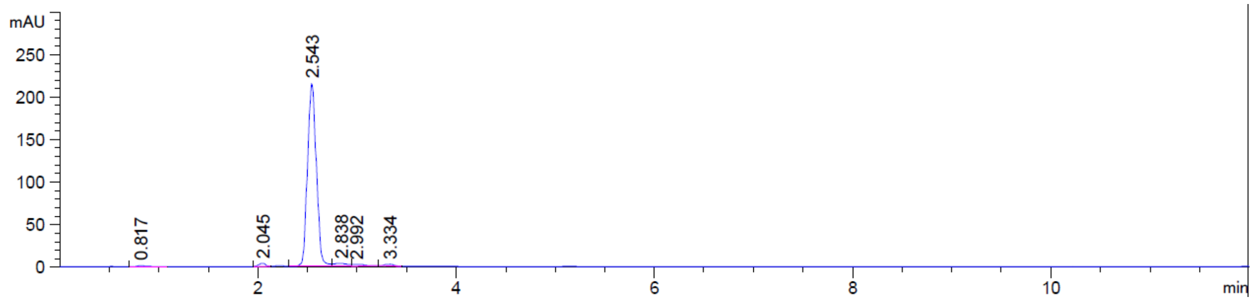

MeO-CyHQ-DA (**3c**)
Reaction monitored at 280 nm for MeO-CyHQ-DA (t_R = 3.1 and 3.2 min), DA (t_R = 1.0 min), and MeO-CyHQ-OH (t_R = 2.2 min).

Photolysis at 365 nm (1PE)
Reaction time = 0 s

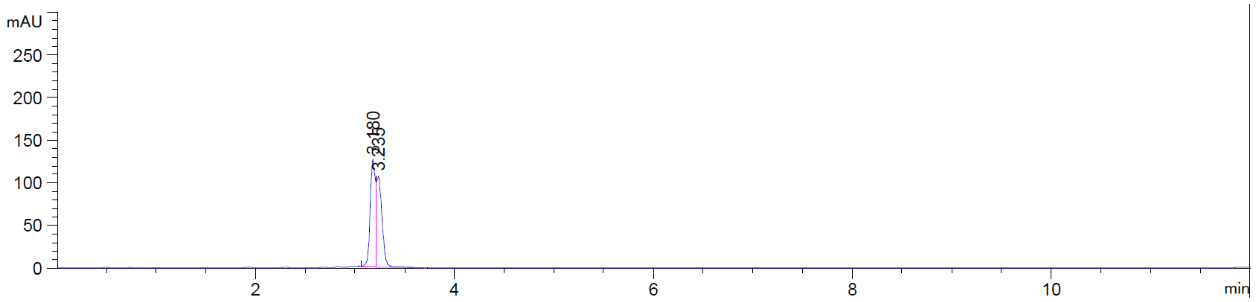

Reaction time = 05 s

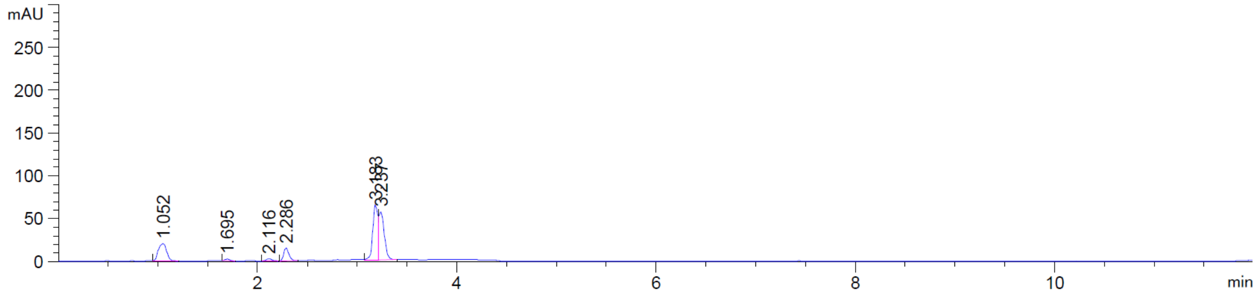

Reaction time = 20 s

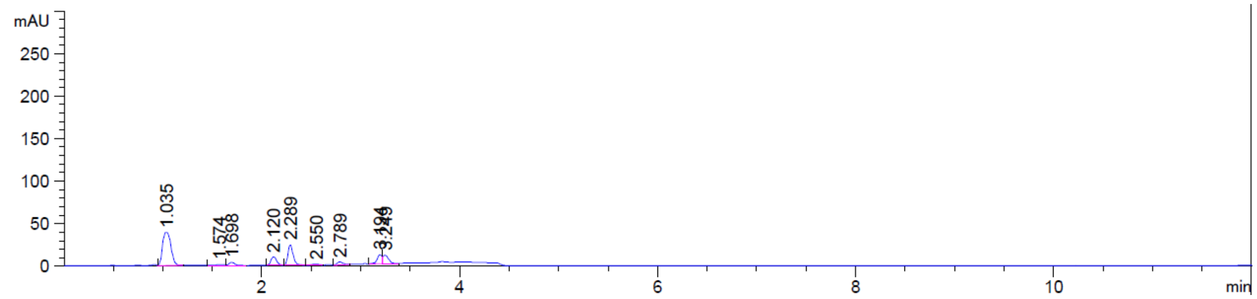

Reaction time = 40 s

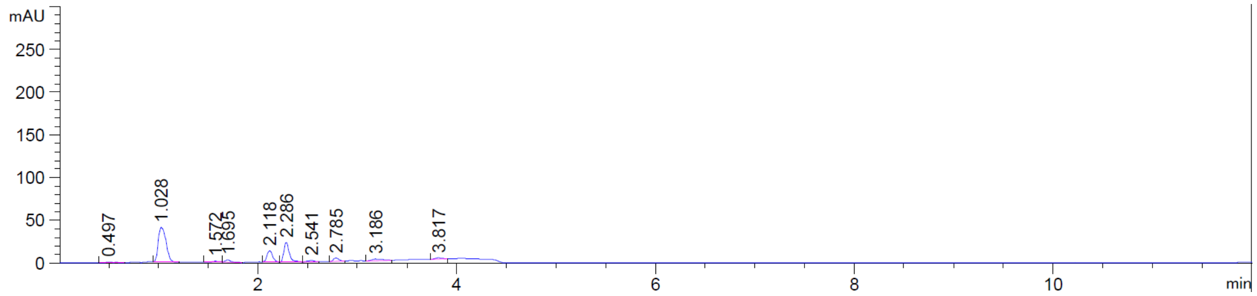

Photolysis at 720 nm (2PE)

Reaction time = 0 min

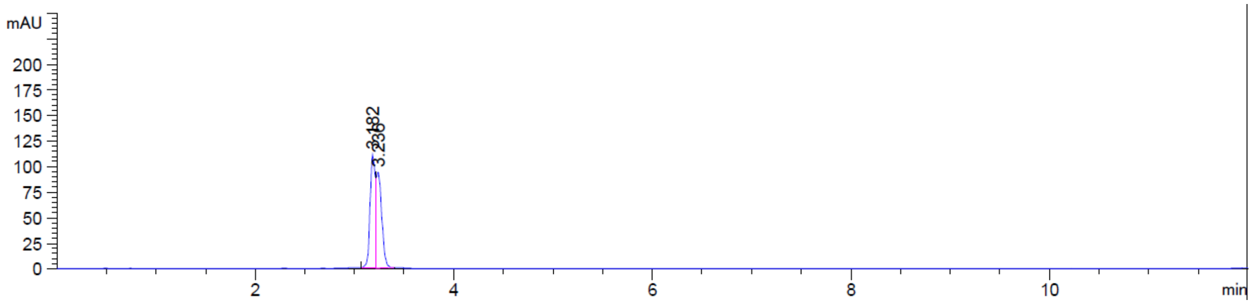

Reaction time = 10 min

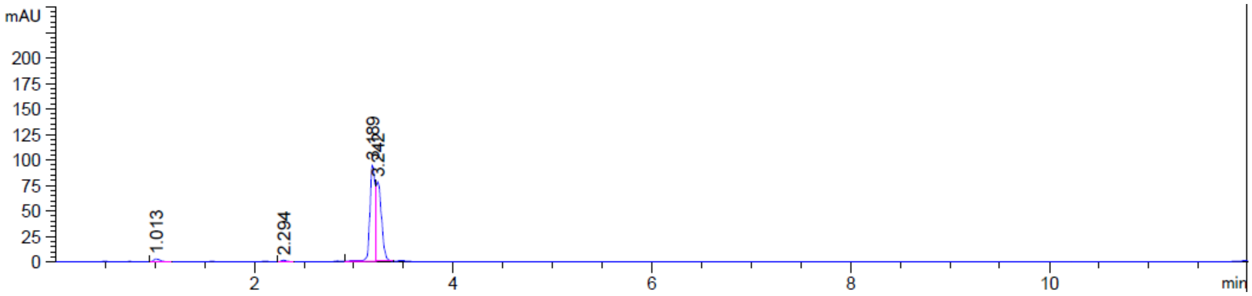

Reaction time = 30 min

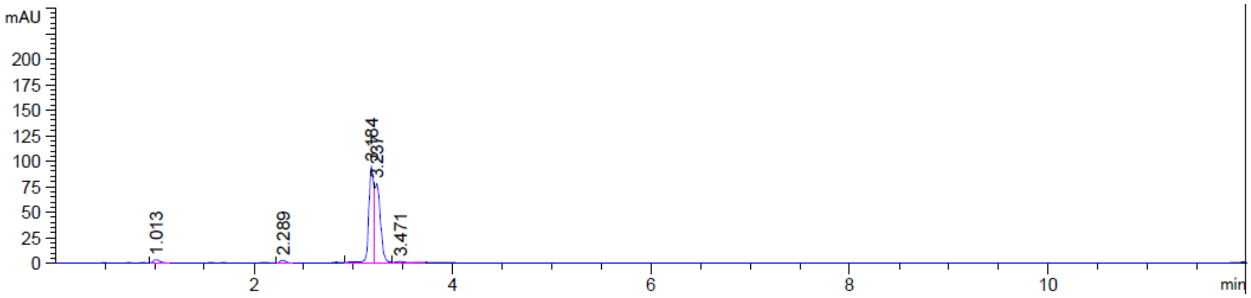

Photolysis at 740 nm (2PE)

Reaction time = 0 min

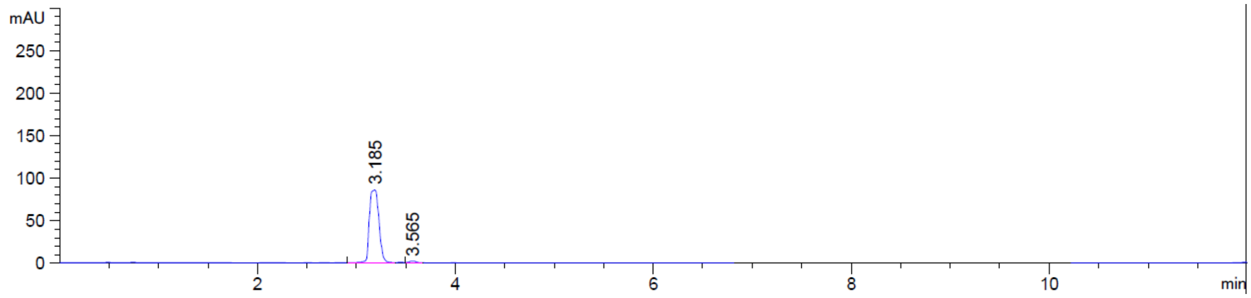

Reaction time = 10 min

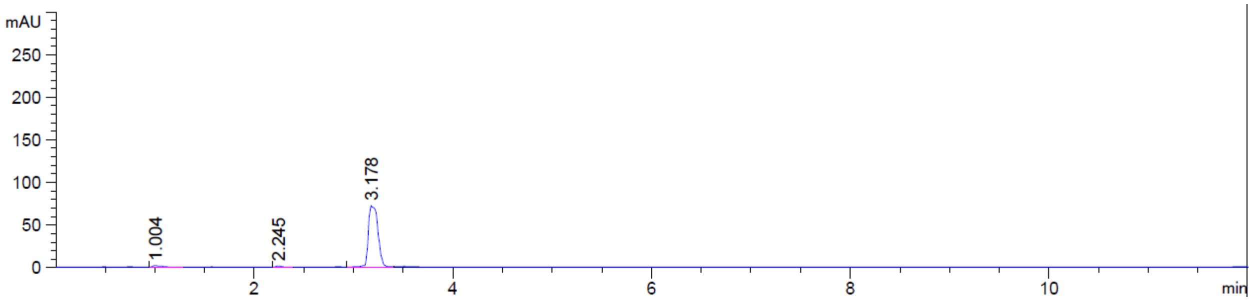

Reaction time = 30 min

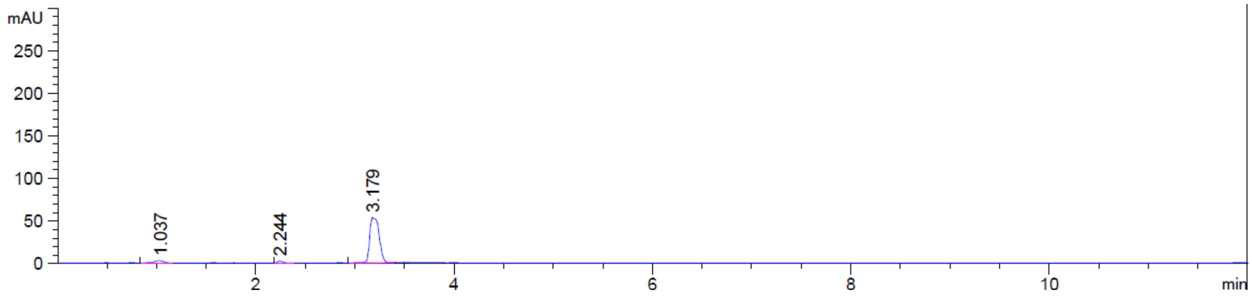

Stability toward spontaneous hydrolysis in the dark

Day 1

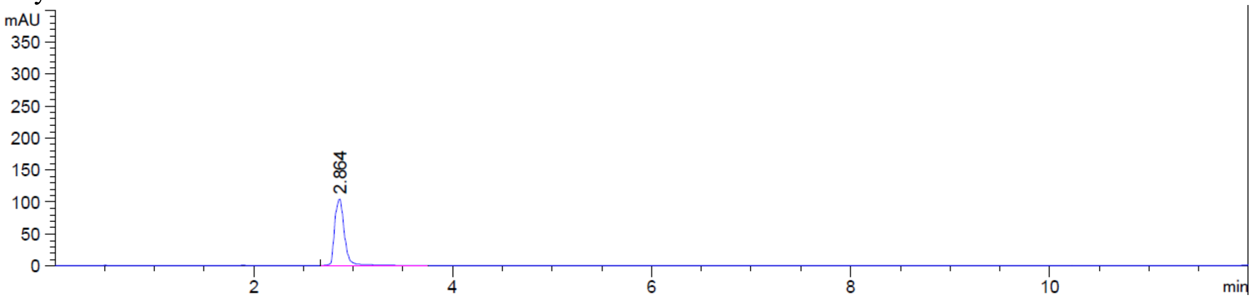

Day 2

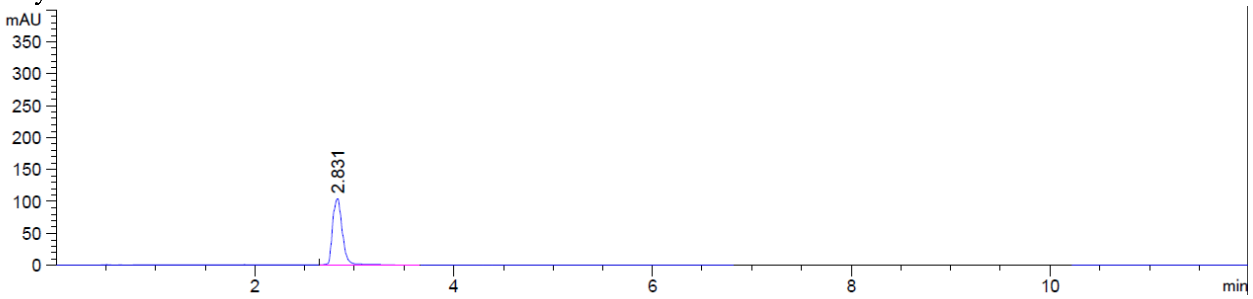

Day 3

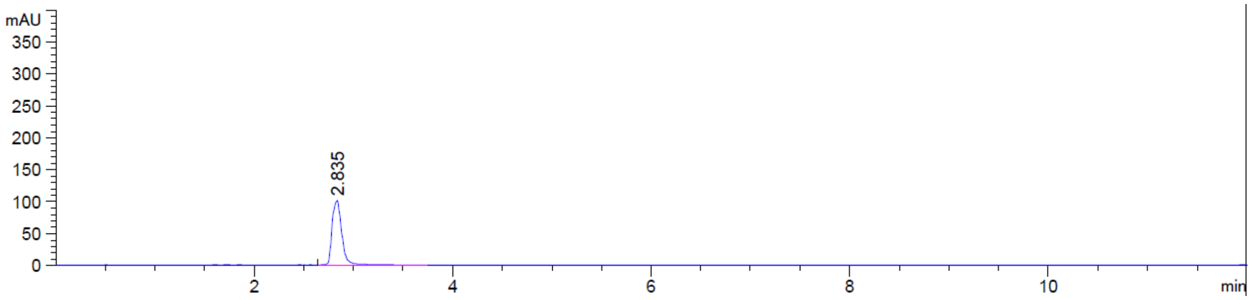

pTol-CyHQ-DA (**3d**)
Reaction monitored at 280 nm for pTol-CyHQ-DA (t_R = 5.9 and 6.2 min), DA (t_R = 0.7 min), and pTol-CyHQ-OH (t_R = 5.6 min).

Photolysis at 365 nm (1PE)
Reaction time = 0 s

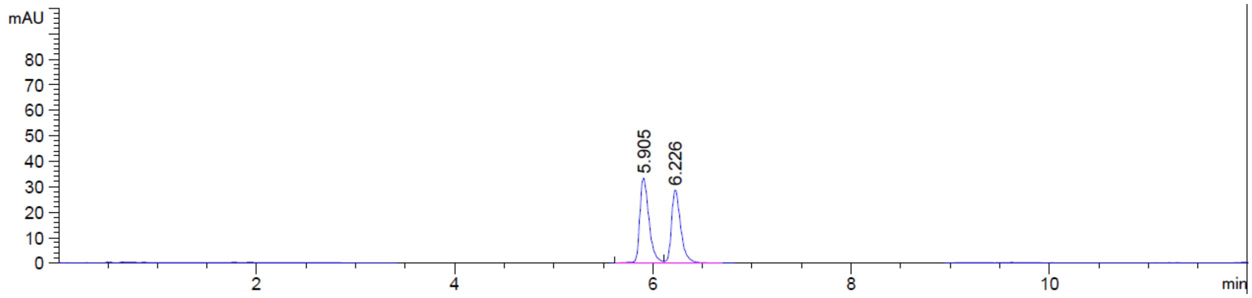

Reaction time = 30 s

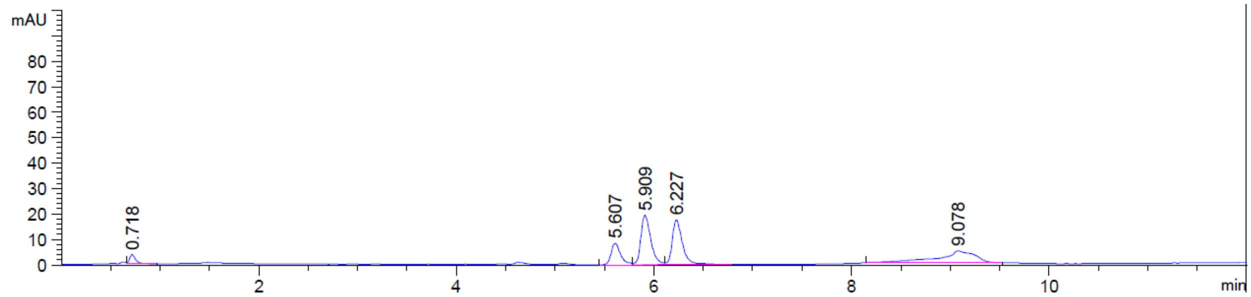

Reaction time = 60 s

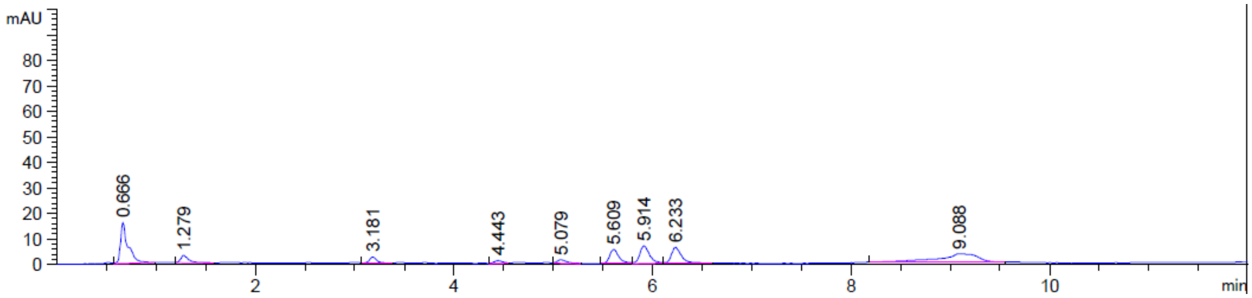

Reaction time = 180 s

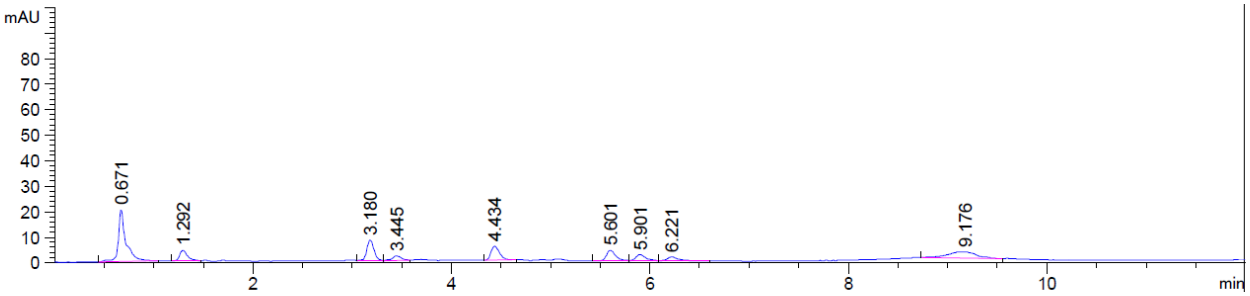

Photolysis at 740 nm (2PE)

External Standard (t_R = 1.8 min)

Reaction time = 0 min

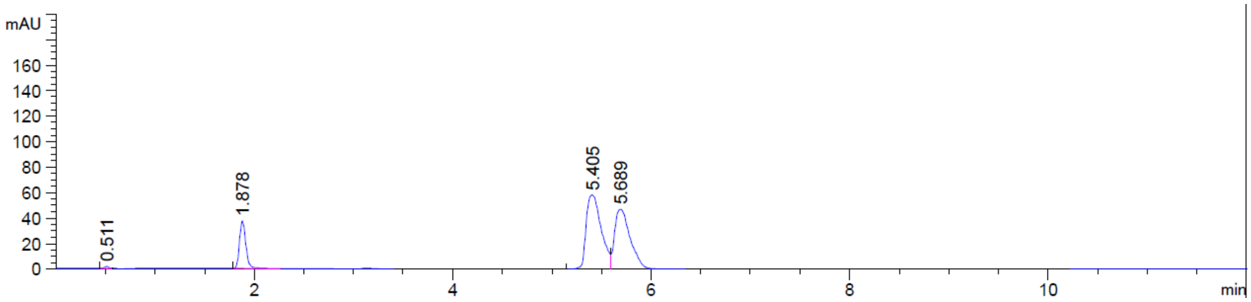

Reaction time = 10 min

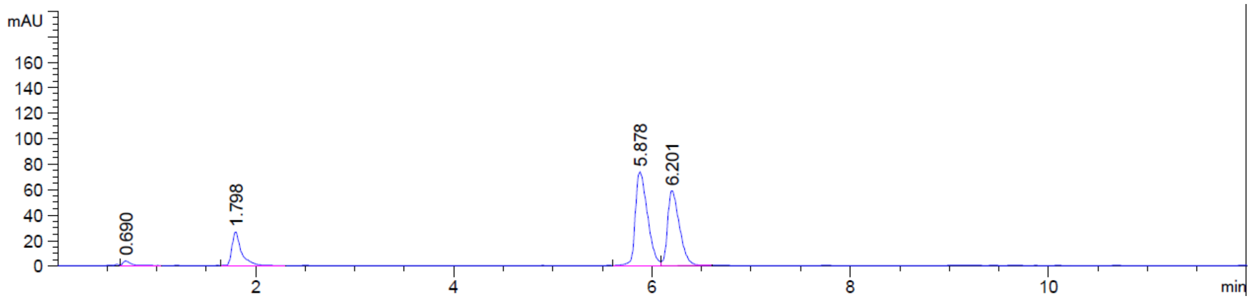

Reaction time = 30 min

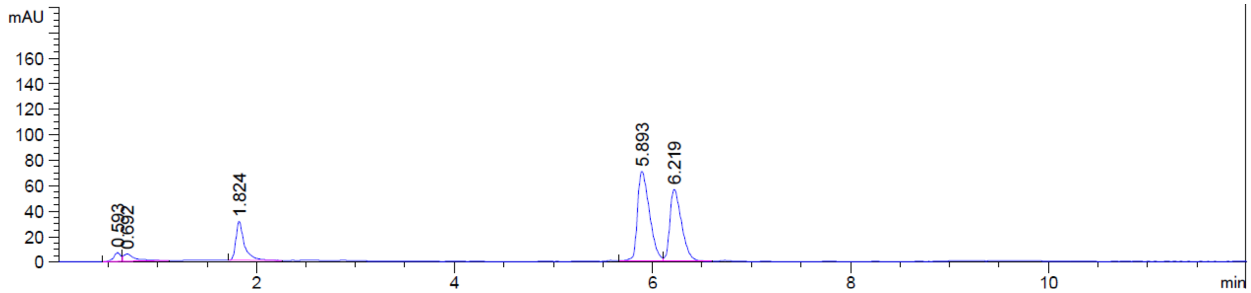

Stability toward spontaneous hydrolysis in the dark

Day 1

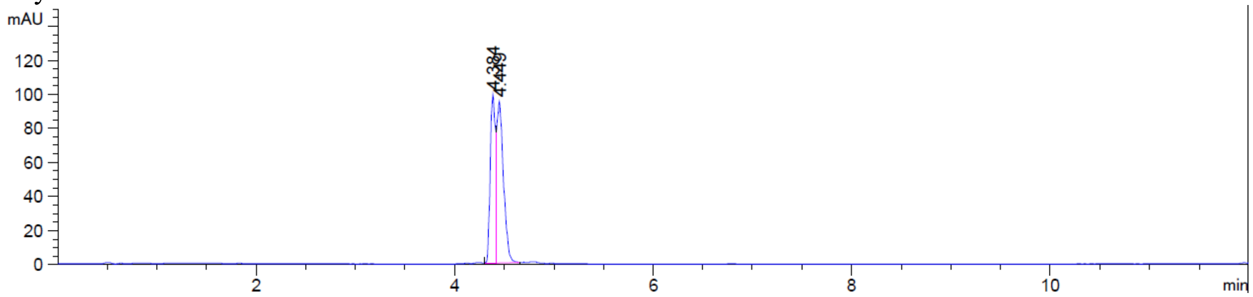

Day 2

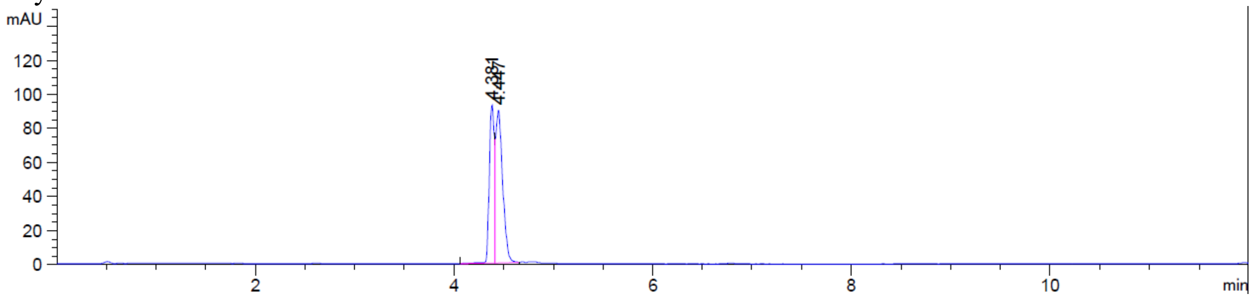

Day 3

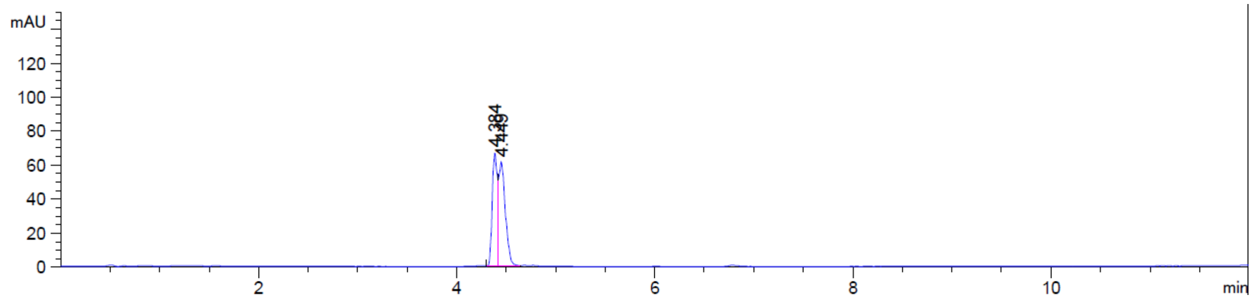

TMP-CyHQ-DA (**3e**)
Reaction monitored at 280 nm for TMP -CyHQ-DA (t_R = 6.9 and 7.0 min), DA (t_R = 1.0 min), and TMP -CyHQ-OH (t_R = 6.6 min).

Photolysis at 365 nm (1PE)
Reaction time = 0 s

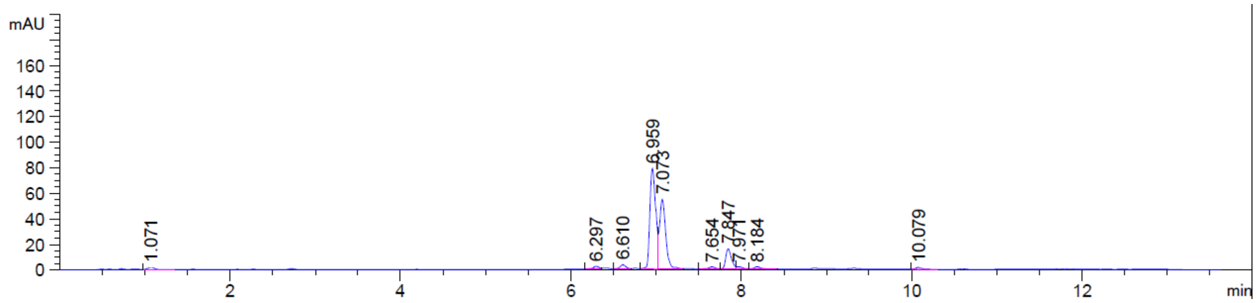

Reaction time = 40 s

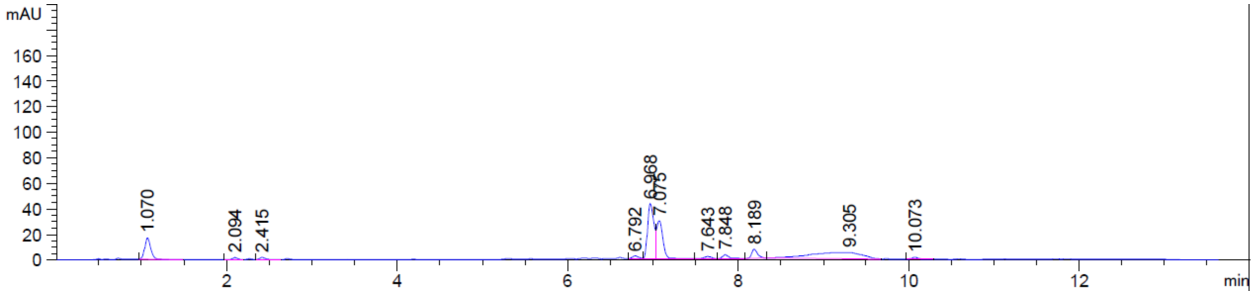

Reaction time = 90 s

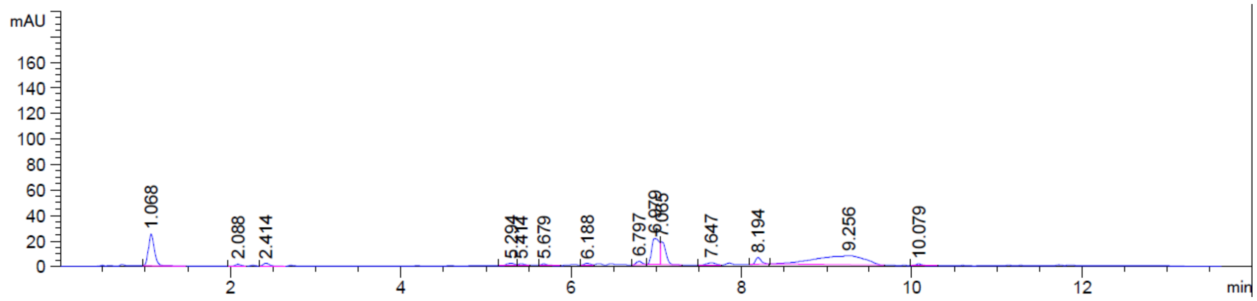

Reaction time = 180 s

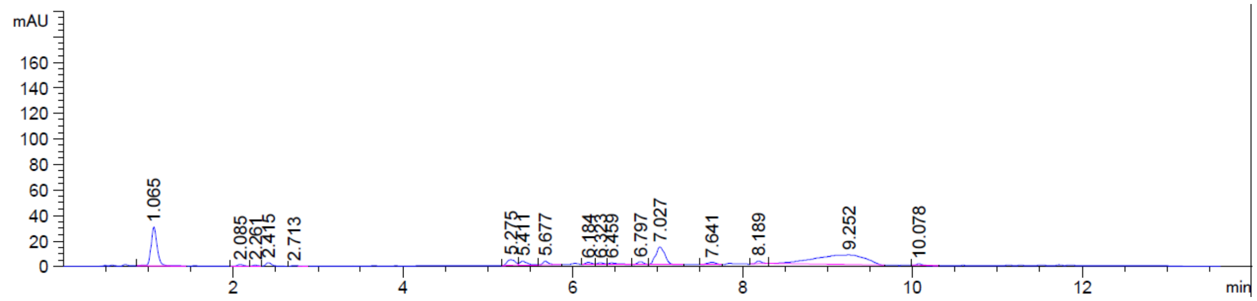

Photolysis at 740 nm (2PE)

Reaction time = 0 min

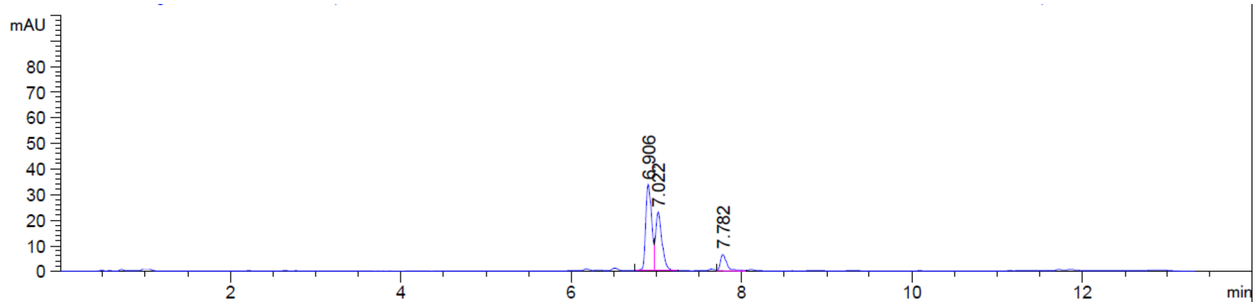

Reaction time = 10 min

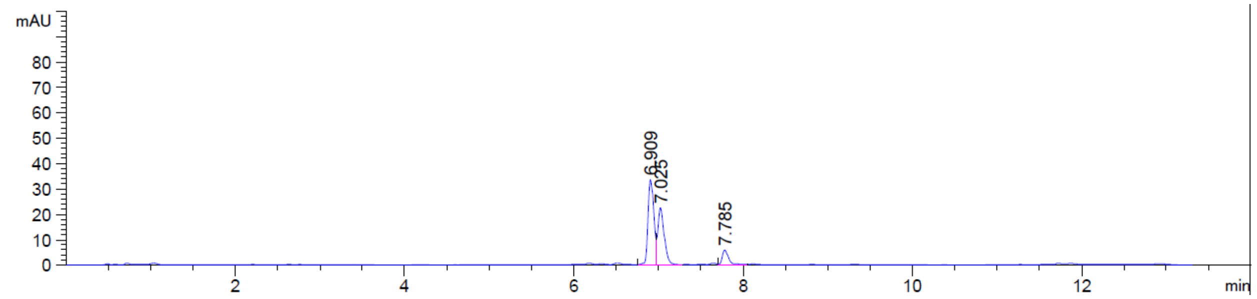

Reaction time = 30 min

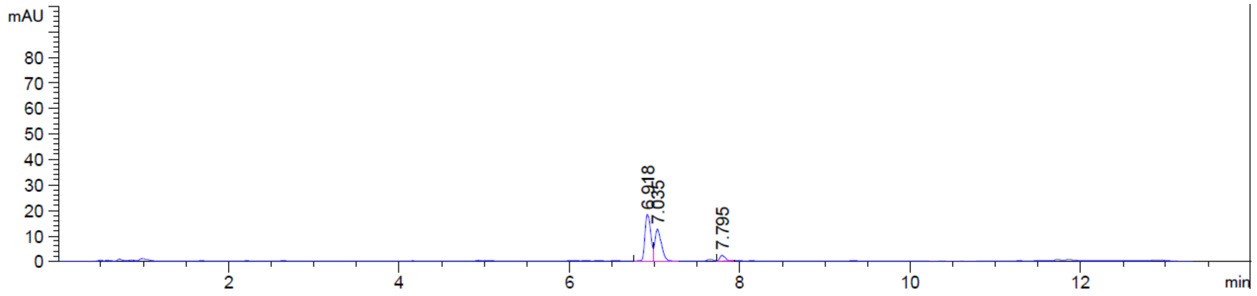

Stability toward spontaneous hydrolysis in the dark

Day 1

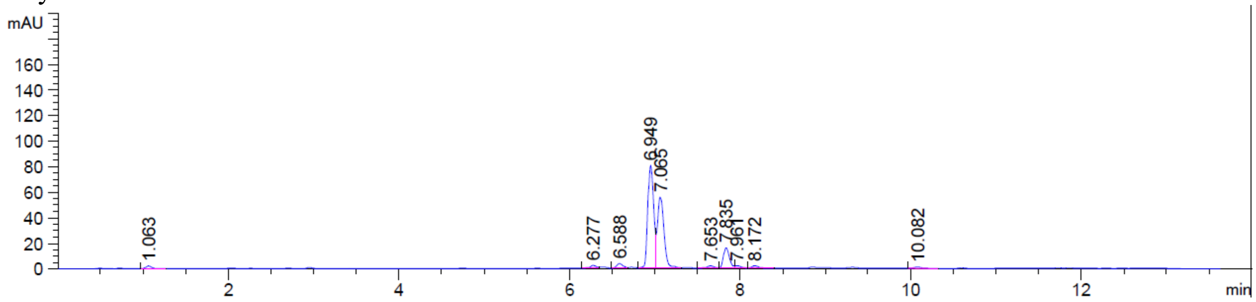

Day 2

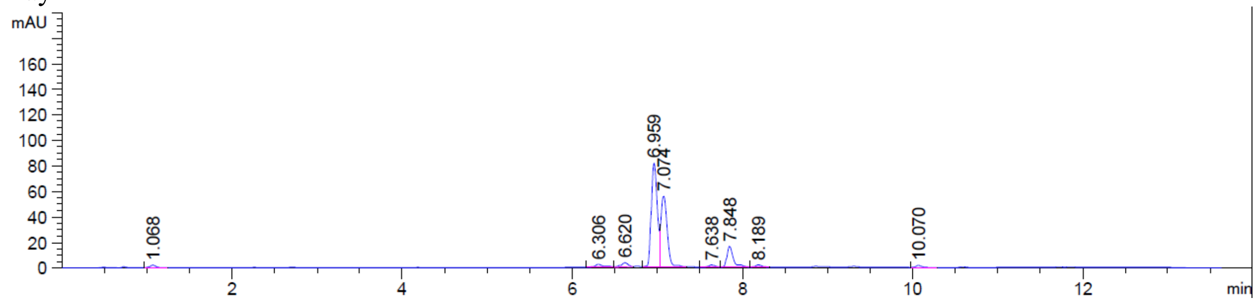

Day 3

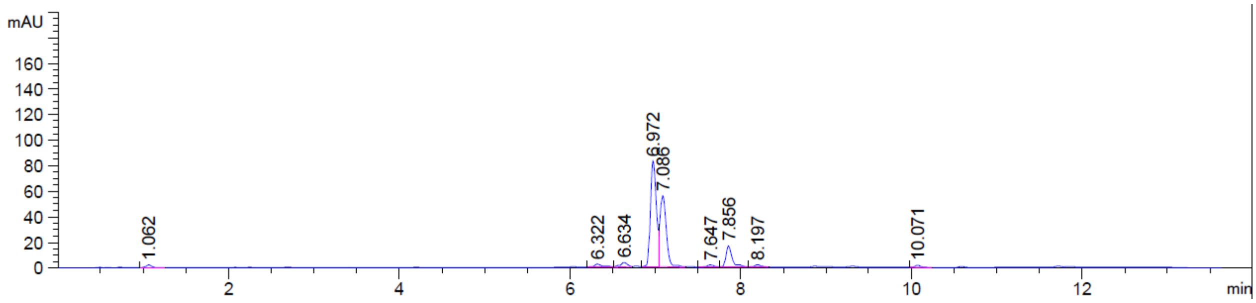

MeO-CyHQ-5HT (**4c**)
Reaction monitored at 280 nm for MeO-CyHQ-5HT (t_R = 3.5 min), 5HT (t_R = 0.8 min), and MeO-CyHQ-OH (t_R = 1.9 min).

Photolysis at 365 nm (1PE)
Reaction time = 0 s

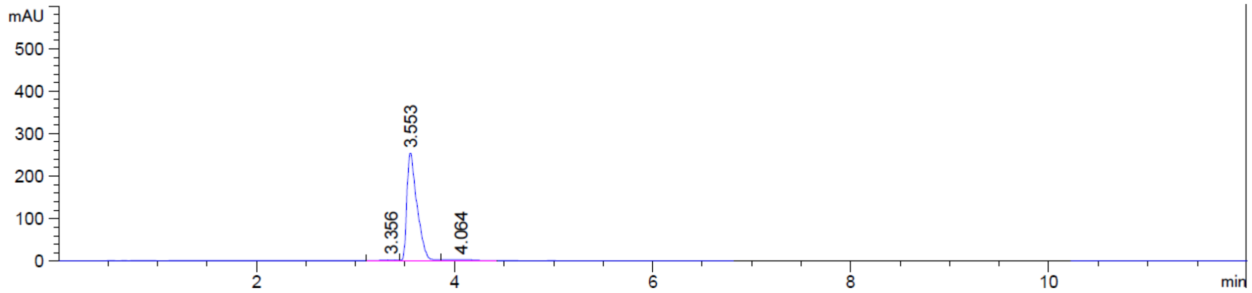

Reaction time = 05 s

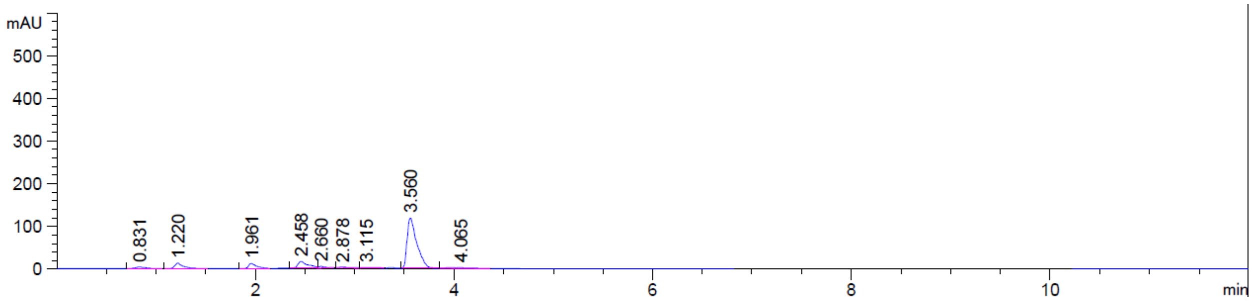

Reaction time = 10 s

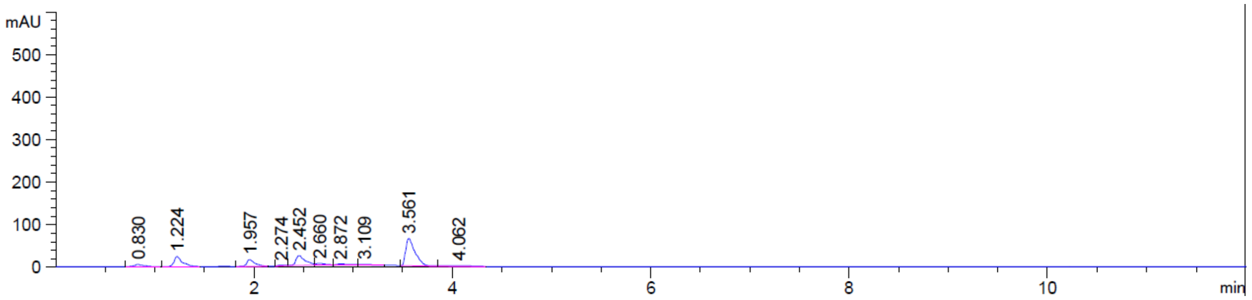

Reaction time = 30 s

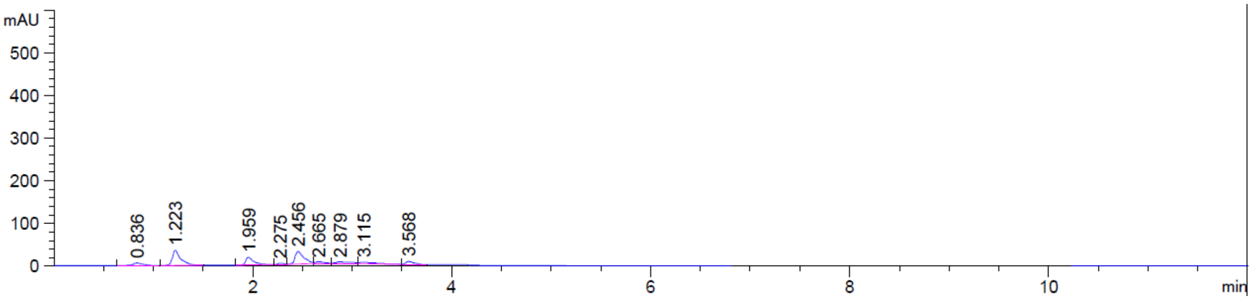

Photolysis at 720 nm (2PE)

Reaction time = 0 min

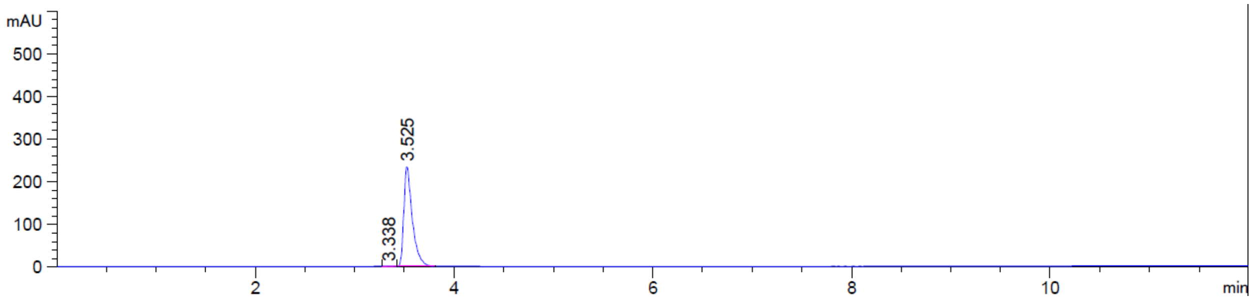

Reaction time = 10 min

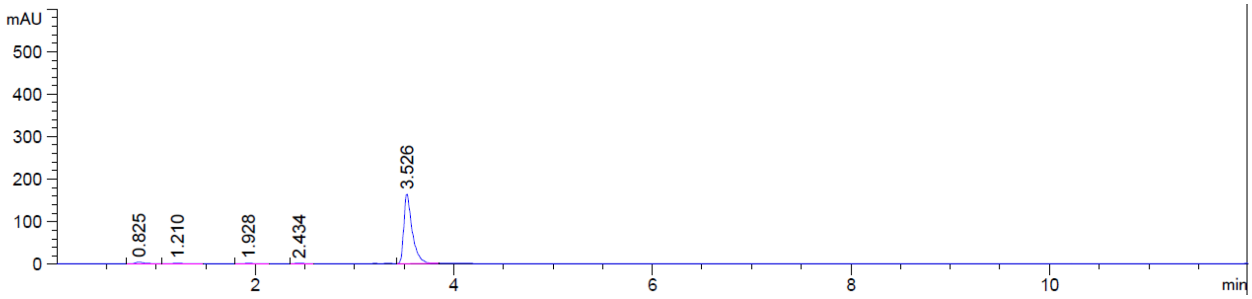

Reaction time = 30 min

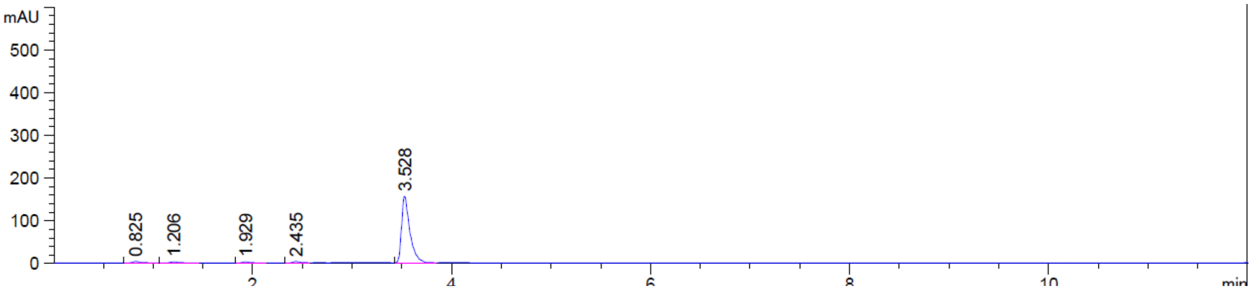

Photolysis at 740 nm (2PE)

Reaction time = 0 min

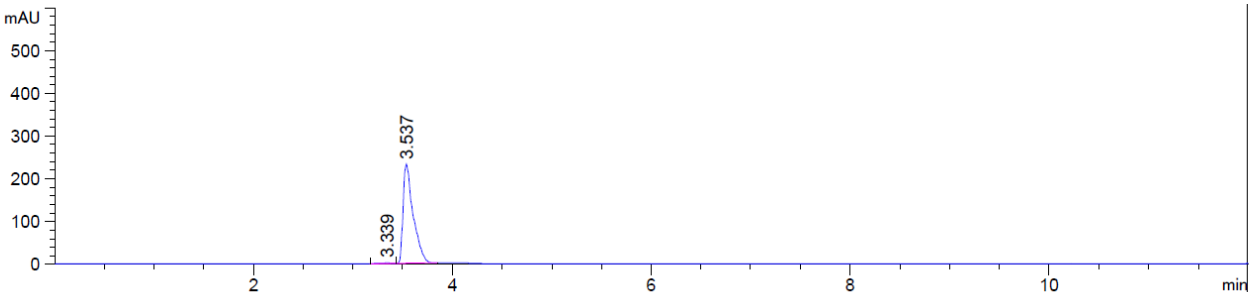

Reaction time = 10 min

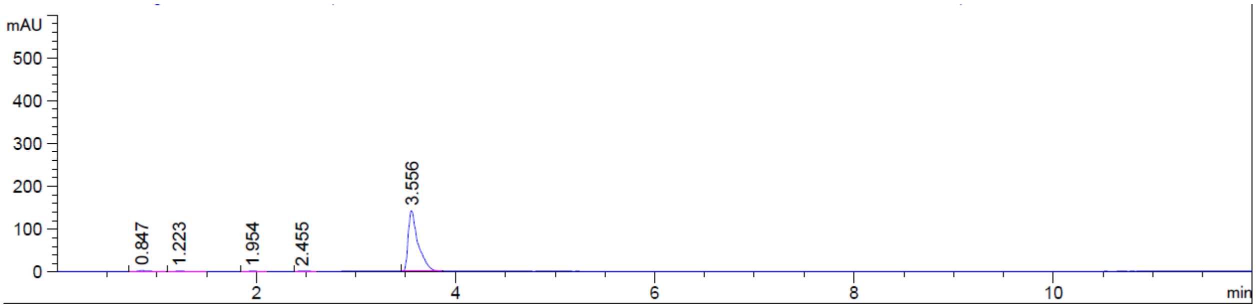

Reaction time = 30 min

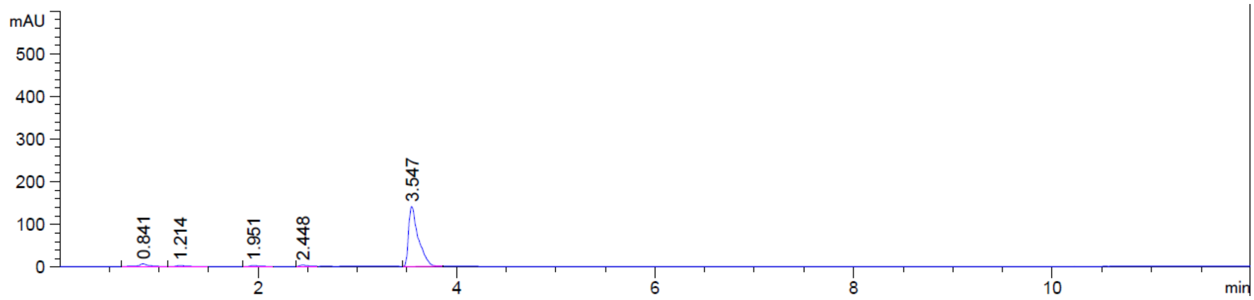

Stability toward spontaneous hydrolysis in the dark

Day 1

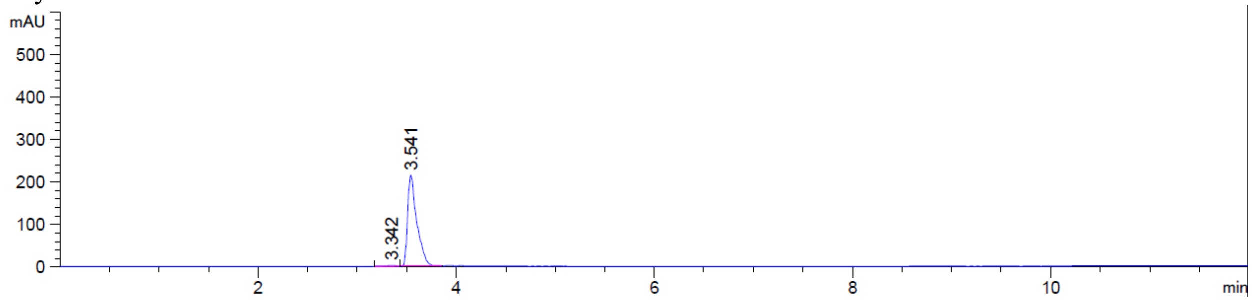

Day 2

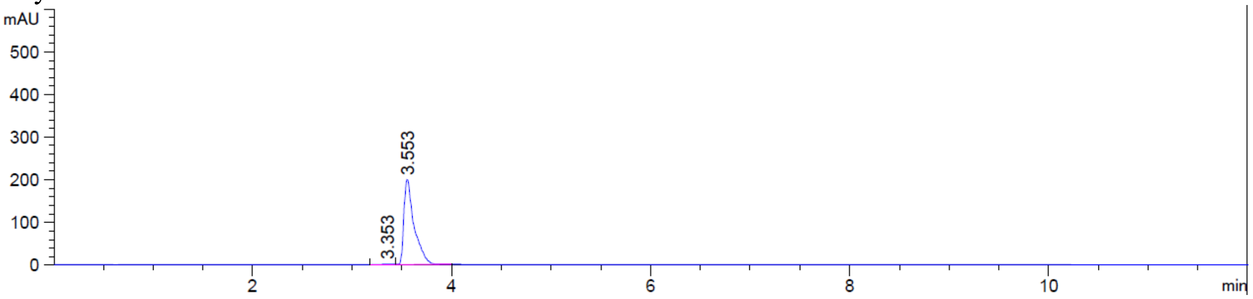

Day 3

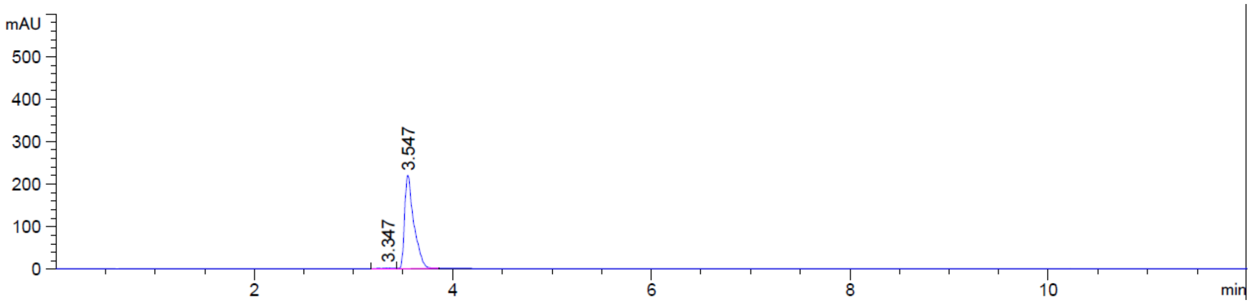

MeO-CyHQ-RTG (**5c**)
Reaction monitored at 254 nm for MeO-CyHQ- RTG (t_R = 5.1 min), RTG (t_R = 3.9 min), and MeO-CyHQ-OH (t_R = 1.9 min).

Photolysis at 365 nm (1PE)
Reaction time = 0 s

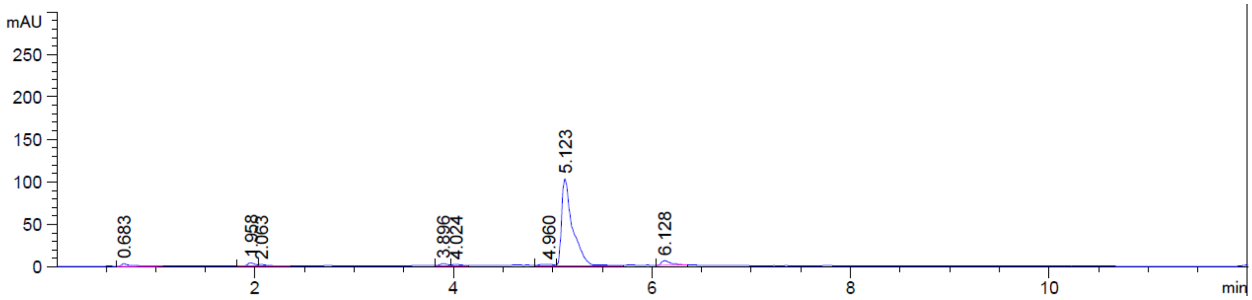

Reaction time = 20 s

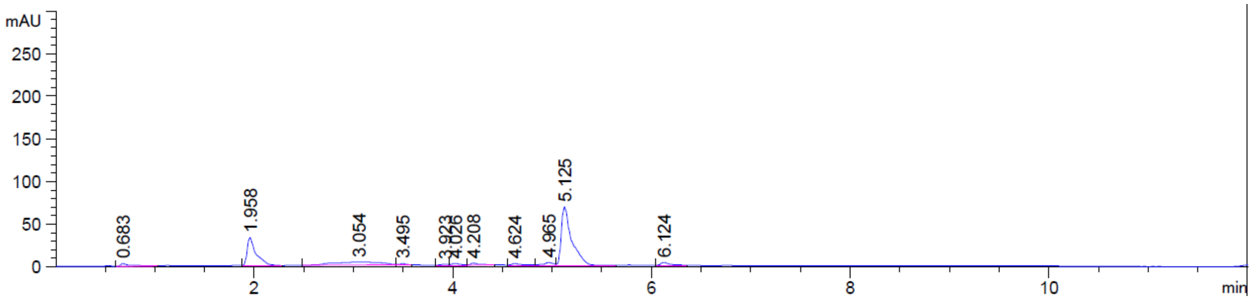

Reaction time = 40 s

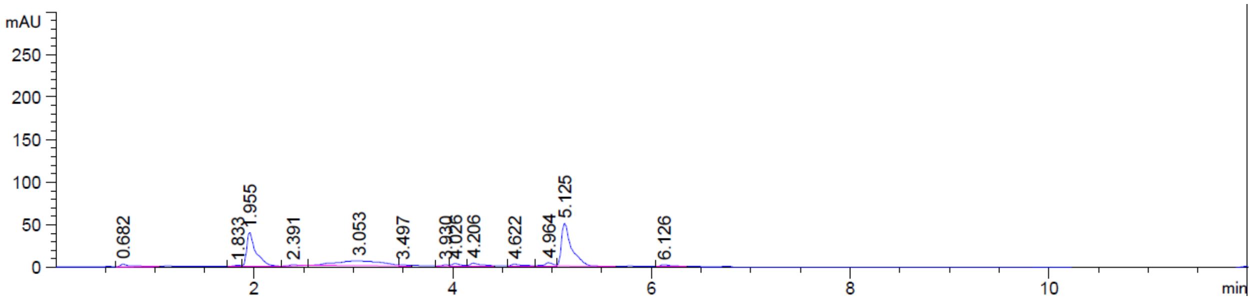

Reaction time = 180 s

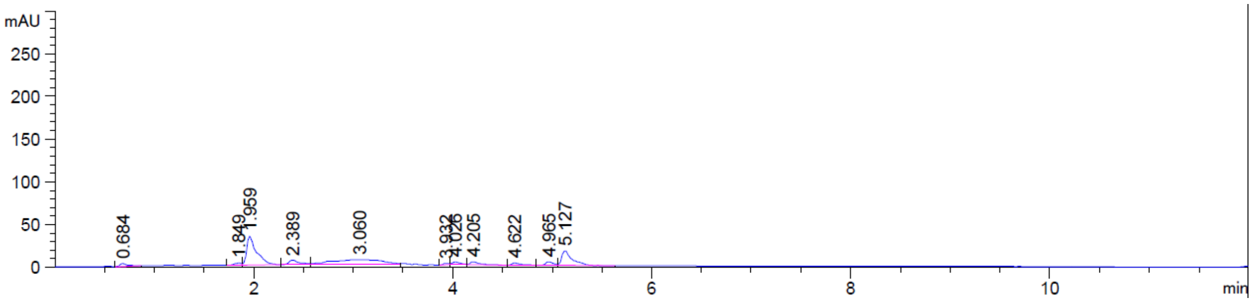

Stability toward spontaneous hydrolysis in the dark

Day 1

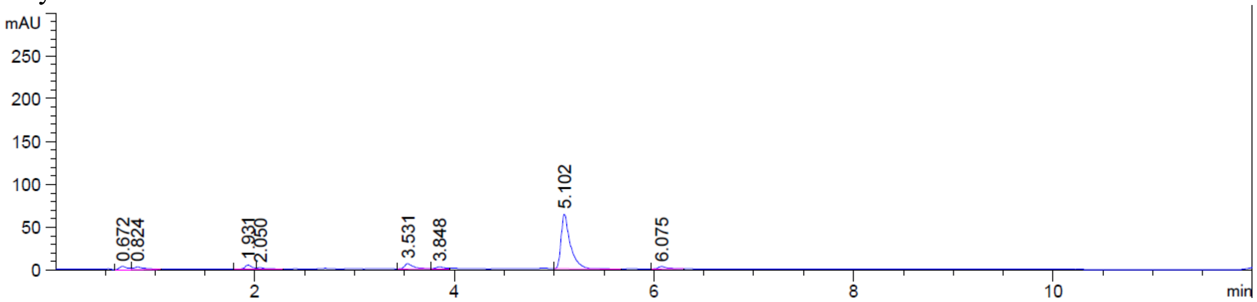

Day 2

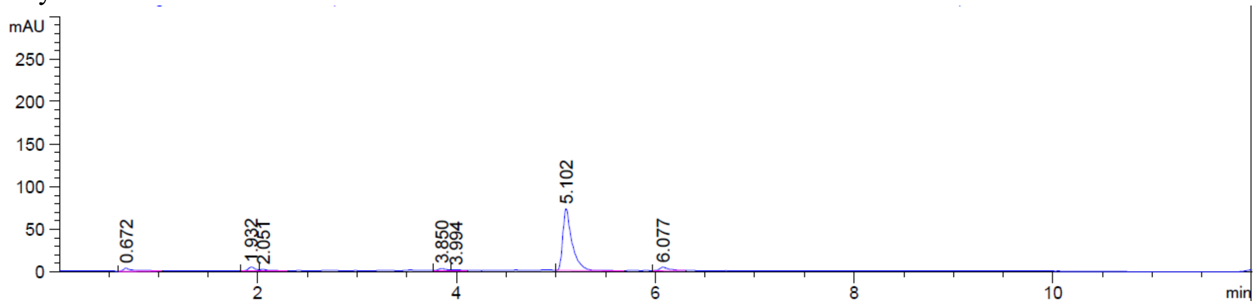

Day 3

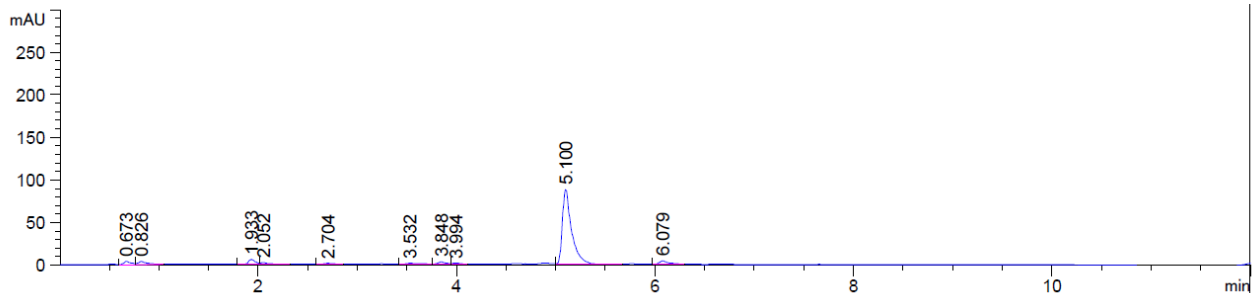

MeO-CyHQ-VNA (**6c**)
Reaction monitored at 280 nm for MeO-CyHQ-VNA (t_R = 6.1 min), VNA (t_R = 5.5 min), and MeO-CyHQ-OH (t_R = 2.1 min).

Photolysis at 365 nm (1PE)
Reaction time = 0 s

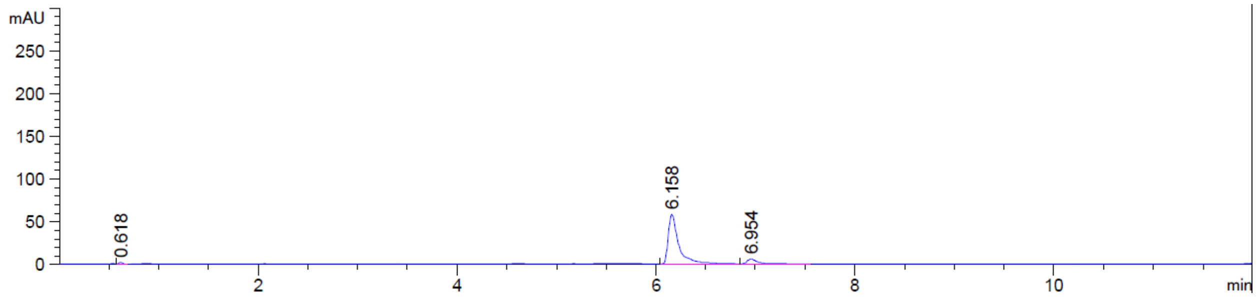

Reaction time = 10 s

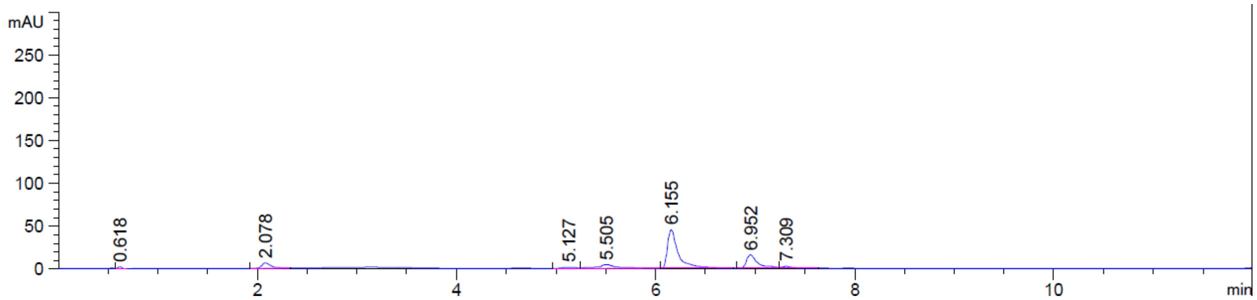

Reaction time = 30 s

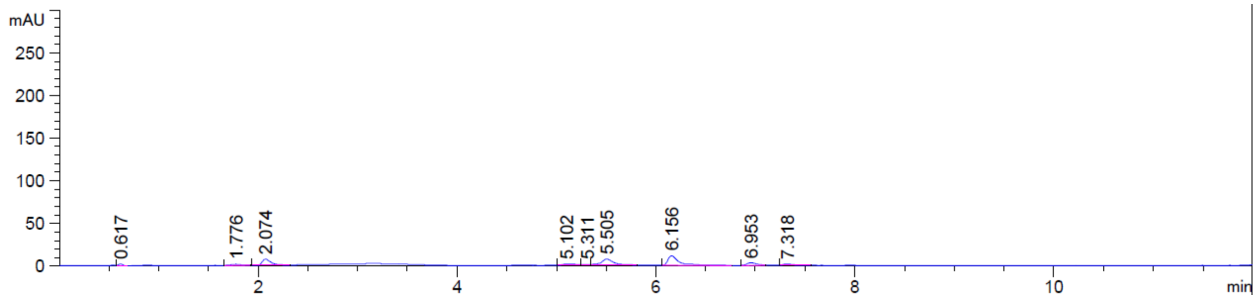

Reaction time = 60 s

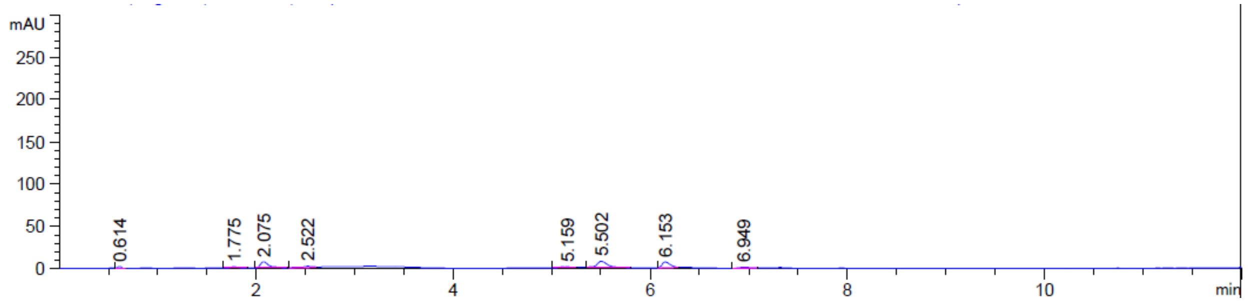

Photolysis at 720 nm (2PE)

Reaction time = 0 min

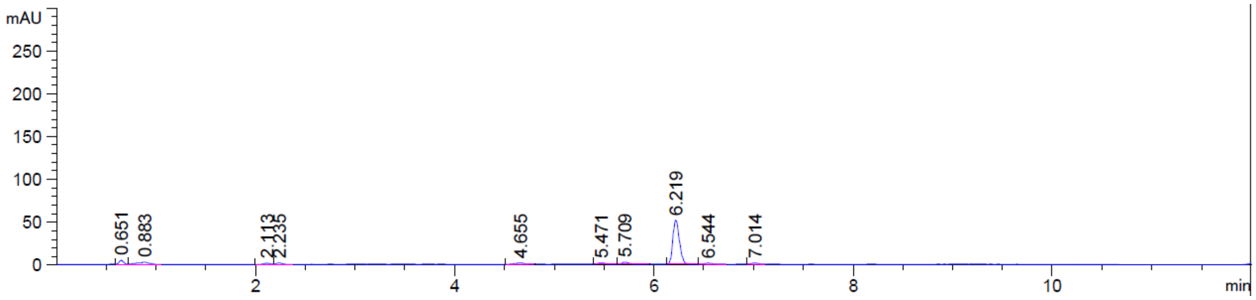

Reaction time = 10 min

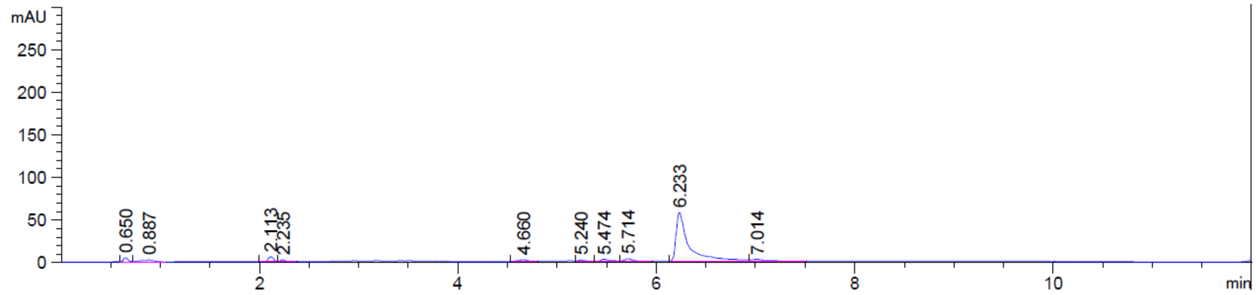

Reaction time = 30 min

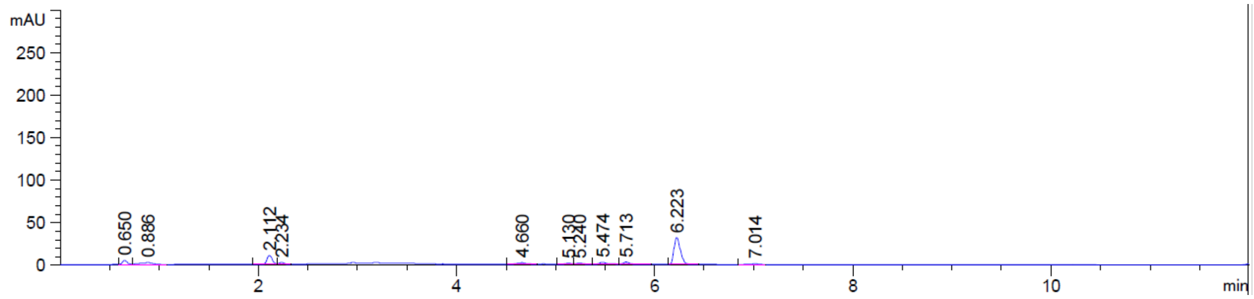

Photolysis at 740 nm (2PE)

Reaction time = 0 min

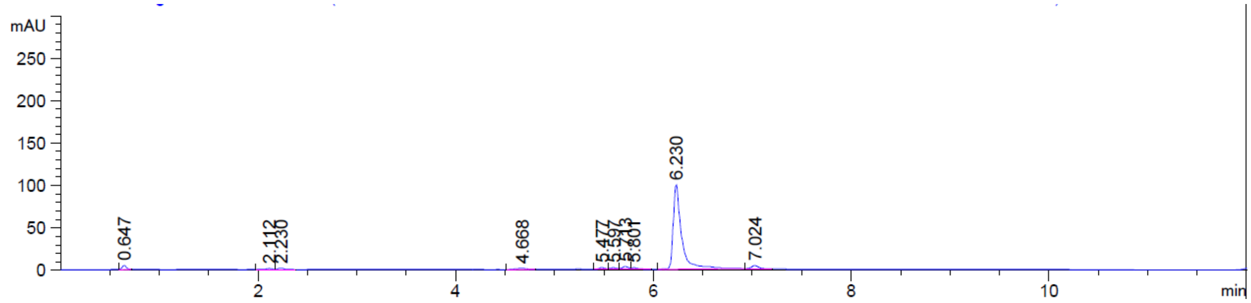

Reaction time = 10 min

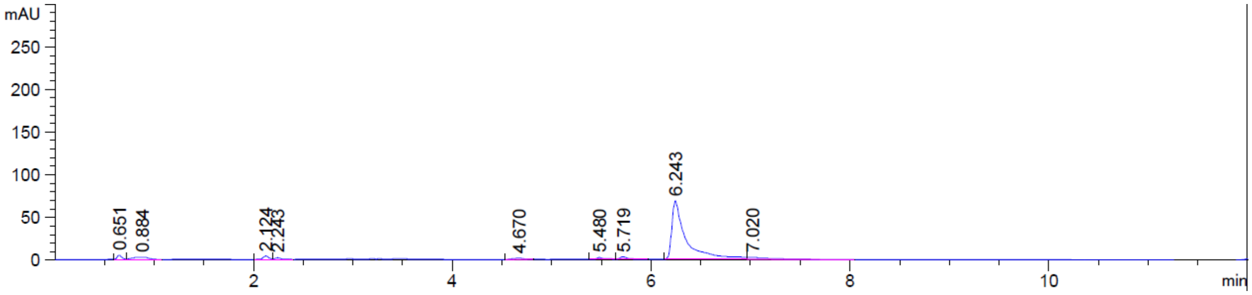

Reaction time = 30 min

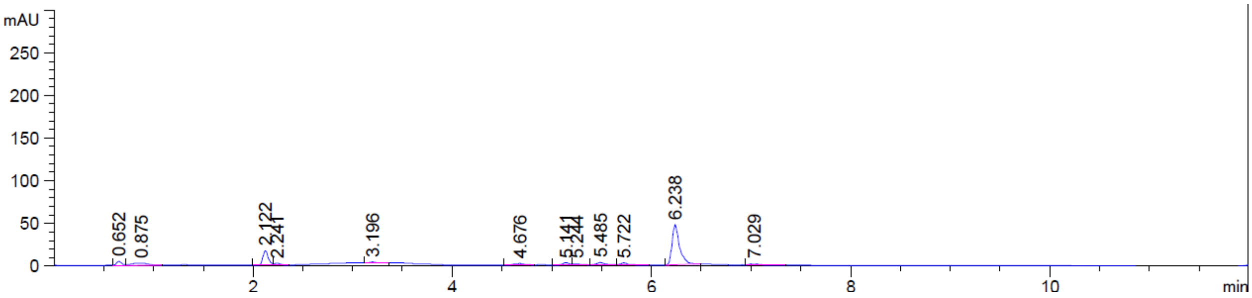

Stability toward spontaneous hydrolysis in the dark

Day 1

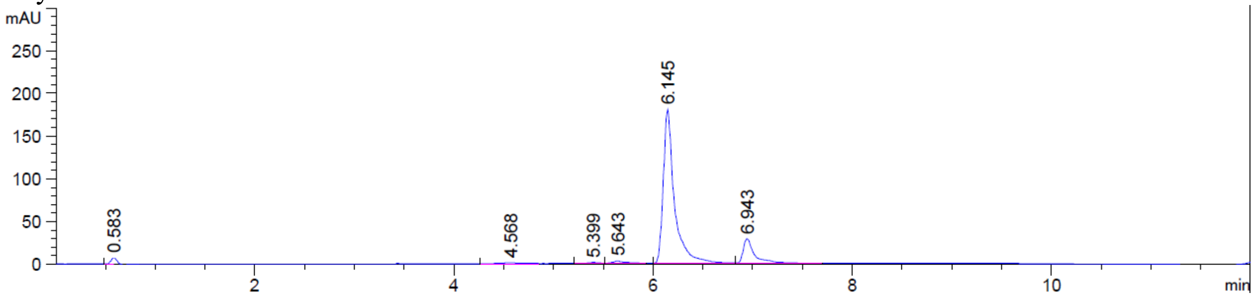

Day 2

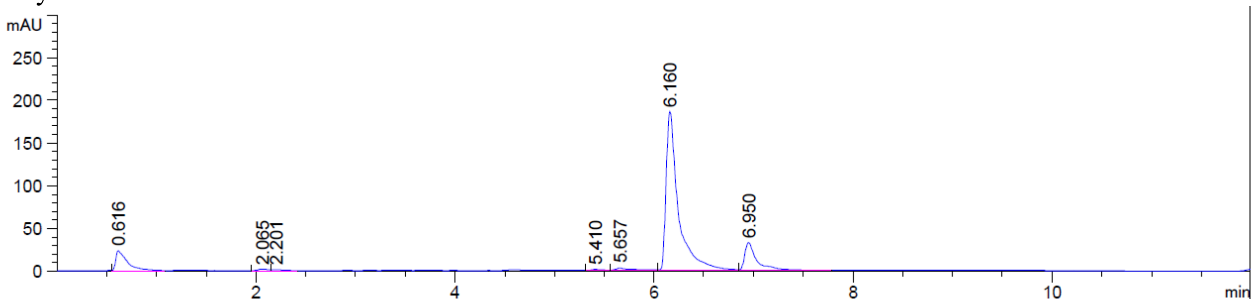

Day 3

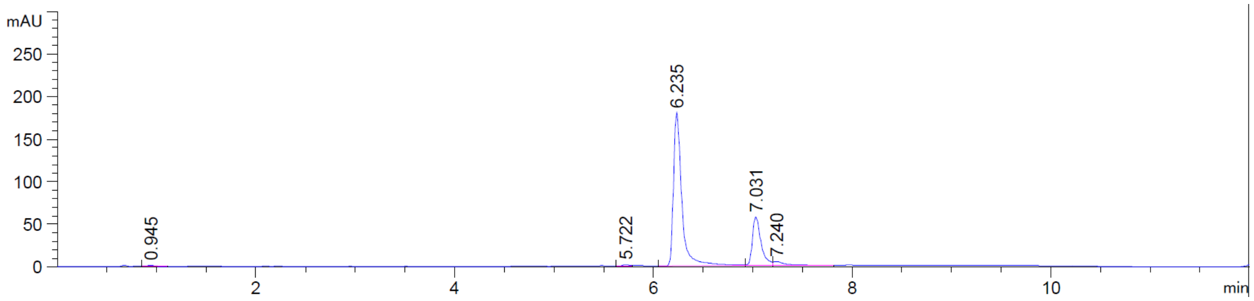

MeO-CyHQ-EG (**7c**)
Reaction monitored at 280 nm for MeO-CyHQ-EG (t_R = 5.7 min), EG (t_R = 4.5 min), and MeO-CyHQ-OH (t_R = 2.1 min).

Photolysis at 365 nm (1PE)
Reaction time = 0 s

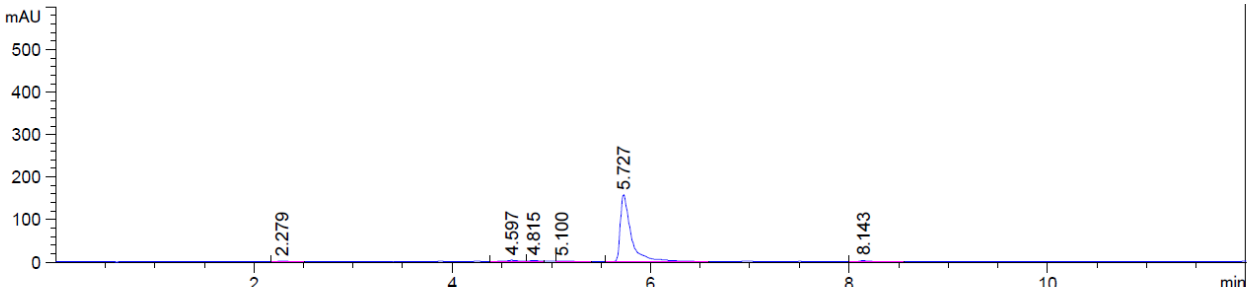

Reaction time = 10 s

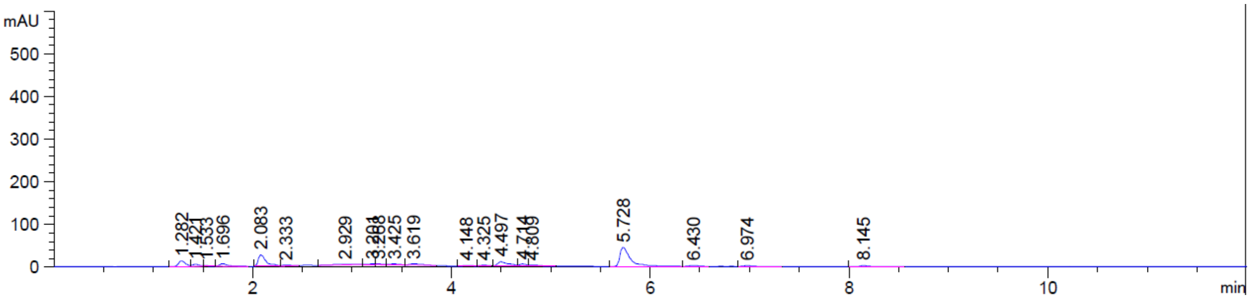

Reaction time = 40 s

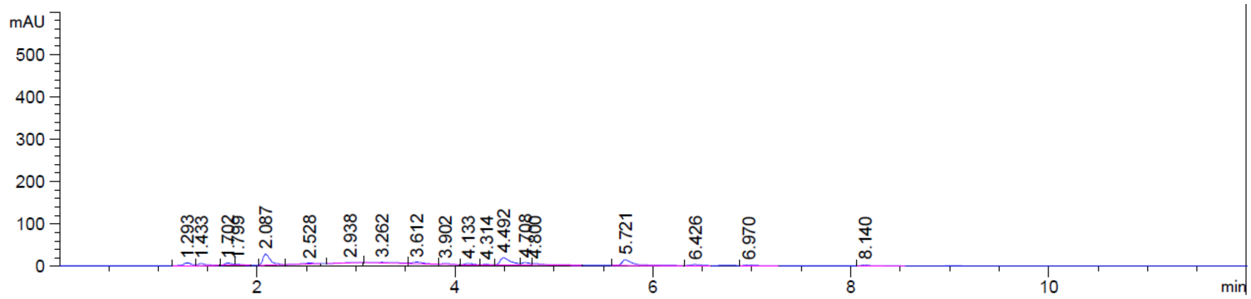

Reaction time = 60 s

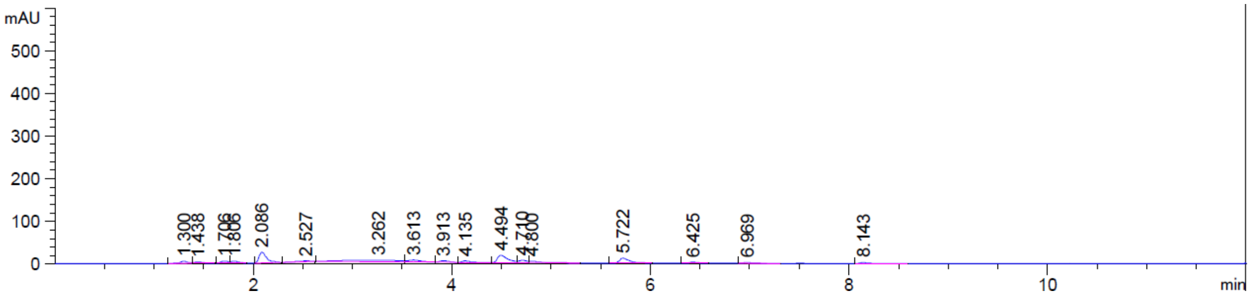

Photolysis at 720 nm (2PE)

Reaction time = 0 min

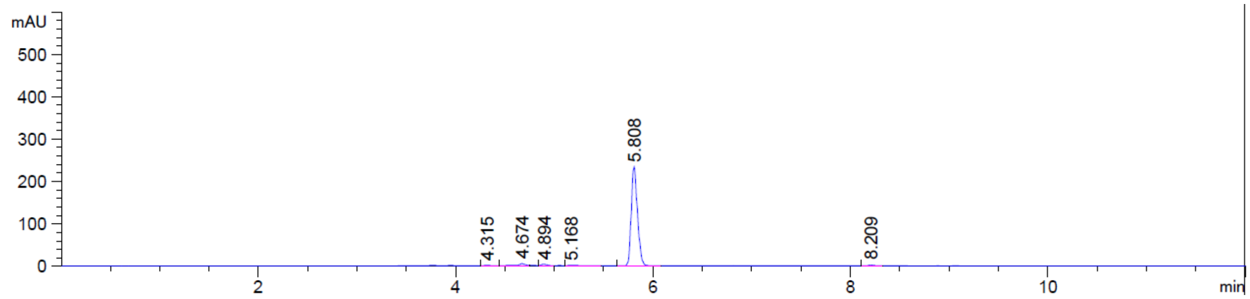

Reaction time = 10 min

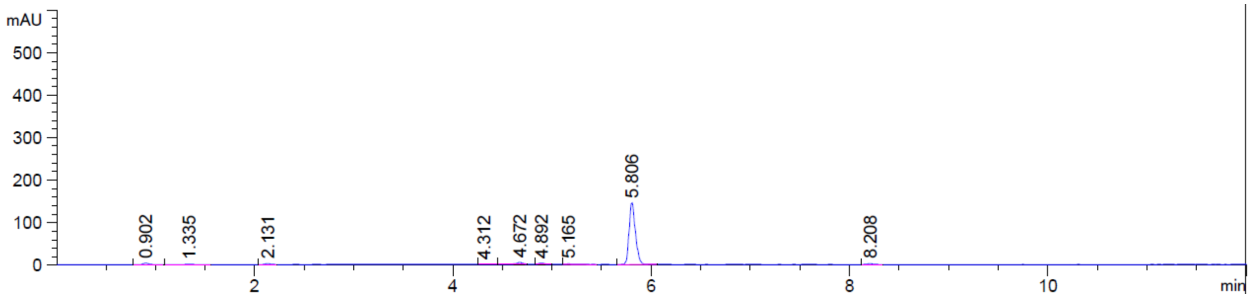

Reaction time = 30 min

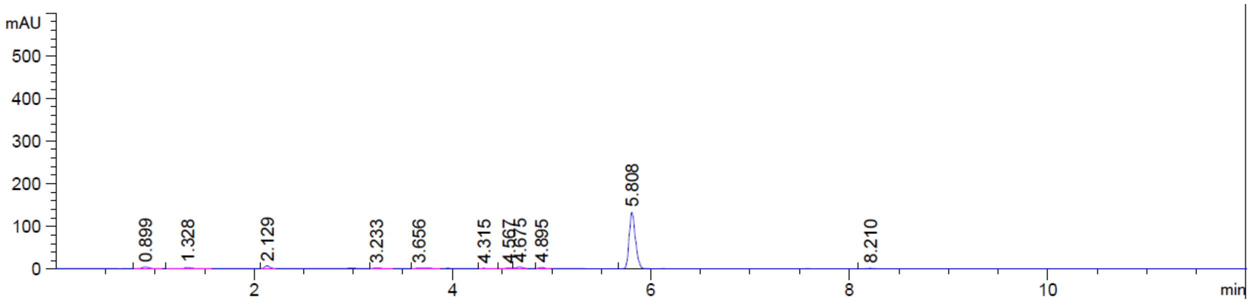

Photolysis at 740 nm (2PE)

Reaction time = 0 min

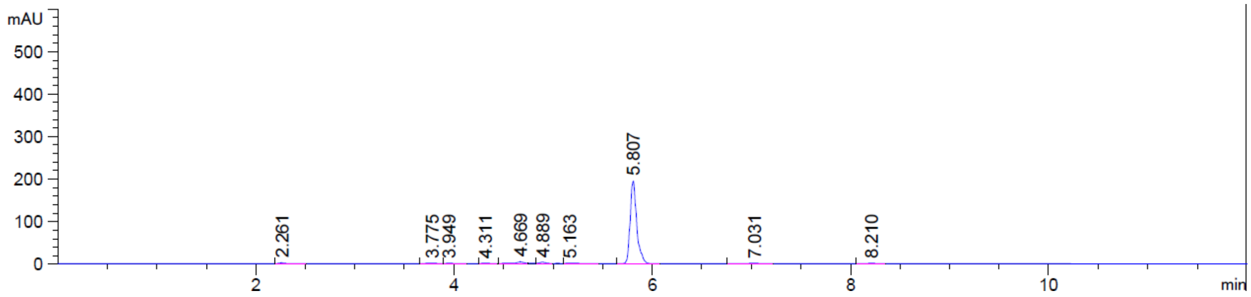

Reaction time = 10 min

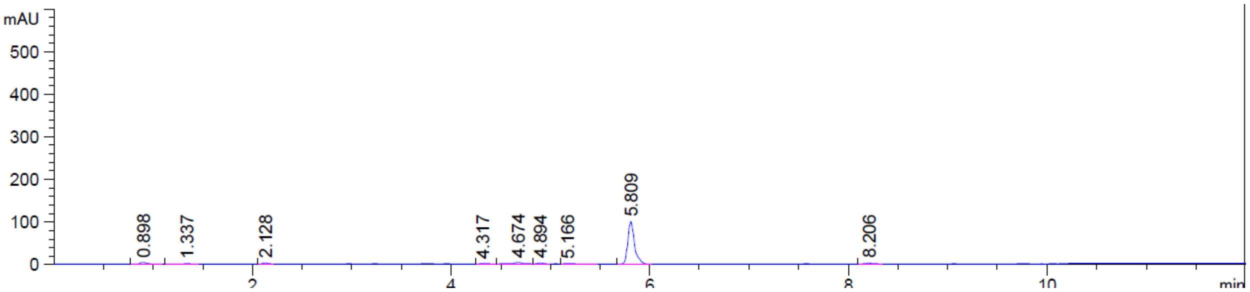

Reaction time = 30 min

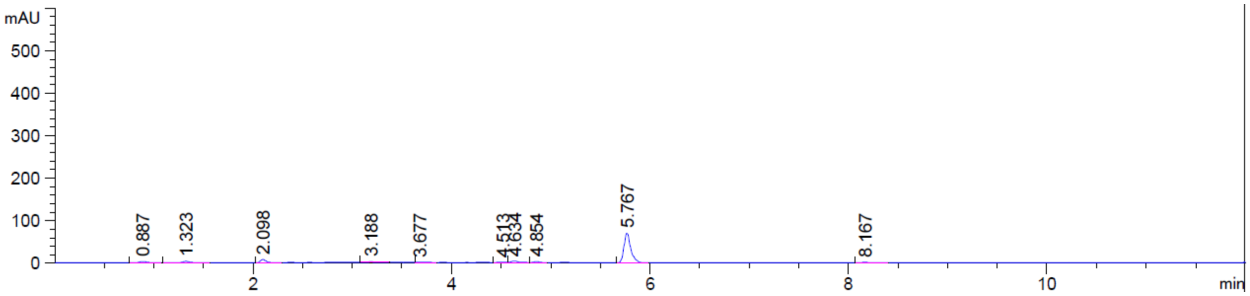

Stability toward spontaneous hydrolysis in the dark

Day 1

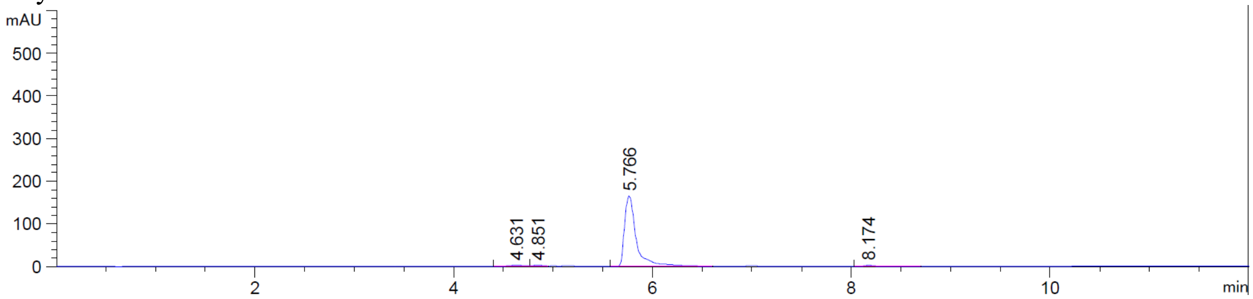

Day 2

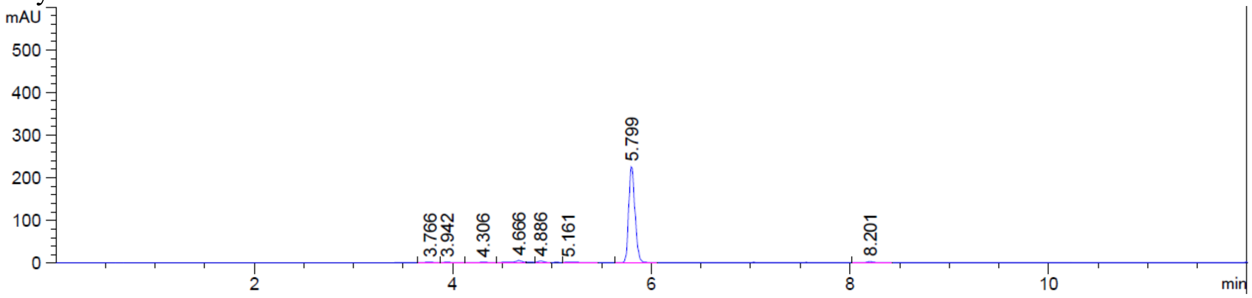

Day 3

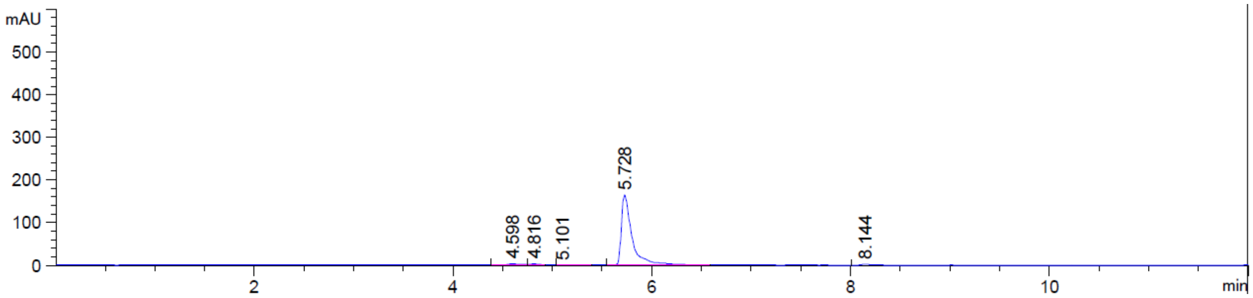

Supplement: Supplementary file 1 — cn3c00552_si_001.pdf [file cn3c00552_si_001.pdf]
